# Synthesis of novel [1,2,4]triazolo[1,5-*b*][1,2,4,5]tetrazines and investigation of their fungistatic activity

**DOI:** 10.3762/bjoc.18.29

**Published:** 2022-03-01

**Authors:** Anna V Korotina, Svetlana G Tolshchina, Rashida I Ishmetova, Natalya P Evstigneeva, Natalya A Gerasimova, Natalya V Zilberberg, Nikolay V Kungurov, Gennady L Rusinov, Oleg N Chupakhin, Valery N Charushin

**Affiliations:** 1 Laboratory of Heterocyclic Compounds, Postovsky Institute of Organic Synthesis, Russian Academy of Sciences, Ural Branch, S. Kovalevskaya str. 22/20, Yekaterinburg, 620108, Russian Federation; 2 Experimental Laboratory Department, Ural Research Institute of Dermatovenerology and Immunopathology, Shcherbakova str. 8, Yekaterinburg, 620076, Russian Federation; 3 Institute of Chemical Engineering, Ural Federal University named after the first President of Russia B. N. Yeltsin, Mira str. 19, Yekaterinburg, 620002, Russian Federation

**Keywords:** antifungal activity, nucleophilic substitution, oxidative cyclization, [1,2,4]triazolo[1,5-*b*][1,2,4,5]tetrazines

## Abstract

A series of novel [1,2,4]triazolo[1,5-*b*][1,2,4,5]tetrazines has been synthesized through oxidation reaction of the corresponding 3,6-disubstituted 1,2,4,5-tetrazines bearing amidine fragments. It is shown that the heterocyclic systems obtained can be modified easily at C(3) position in the reactions with aliphatic alcohols and amines. Also, the reactivity of [1,2,4]triazolo[1,5-*b*][1,2,4,5]tetrazines towards CH-active compounds has been studied. The obtained triazolo[1,5-*b*]annulated 1,2,4,5-tetrazines proved to be active in micromolar concentrations in vitro against filamentous anthropophilic and zooanthropophilic dermatophyte fungi (*Trichophyton*, *Microsporum* and *Epidermofiton*), which cause skin and its appendages (hair, nails) diseases.

## Introduction

Azolo-annulated azines can be regarded as purine isosteres and are of great interest for modern medicinal chemistry as potential biologically active compounds. In particular, imidazo[1,2-*a*]pyridines and imidazo[1,2-*a*]pyrimidines exhibit a wide spectrum of biological activity, including antiviral and antibacterial ones [1–2].

A promising approach for the development of new drugs is the synthesis and bioscreening of high nitrogen-containing azoloazines, including azolo-annulated tetrazines. These compounds, bearing a large number of heteroatoms in their structures, have additional opportunities for non-covalent bonding with a variety of biological targets. In addition, a high electrophilic character of the tetrazine ring can provide chemical binding to pathogenic objects, thus leading to a disturbance of their functions.

Up to date, there is a considerable data set, providing an evidence that azolo-annulated 1,2,4,5-tetrazines have a pronounced biological activity. It is worth, for instance, to mention antimicrobial activity of 1,4-dihydroimidazo[1,2-*b*][1,2,4,5]tetrazines [3], antibacterial, antifungal, and anti-infammatory activities of dihydro- and tetrahydro[1,2,4]triazolo[4,3-*b*][1,2,4,5]tetrazines [4–6], antituberculosis activity of [1,2,4]triazolo[4,3-*b*]- and imidazo[1,2-*b*][1,2,4,5]tetrazines [7–9], antibacterial and fungistatic action of thiazolo[3,2-*b*][1,2,4,5]tetrazines [10], fungistatic activity of imidazo[1,2-*b*][1,2,4,5]tetrazines [11], antibacterial and antiglycation activity of imidazo[1,2-*b*]- and [1,2,4]triazolo[4,3-*b*][1,2,4,5]tetrazines [12–14] as well as antitumor activity of [1,2,4]triazolo[4,3-*b*][1,2,4,5]tetrazines [15–16] and imidazo[1,2-*b*][1,2,4,5]tetrazines [17–18]. Thus, a search for new effective drugs in the series of azolo[1,2,4,5]tetrazines appears to be an important and promising area of research.

1,2,4,5-Tetrazine derivatives have been actively studied in recent years [19–22]. These compounds are attractive as high-energy materials [23–25], molecules with promising photophysical, electrochemical and coordination properties for use in sensors, OLEDs, semiconductor materials, etc. [26–28]. The most important biomedical application of 1,2,4,5-tetrazines are bioorthogonal reactions that allow labeling proteins and visualizing cancer due to the ability of *s*-tetrazine to fast and biocompatible ligation with alkenes via the inverse electron demand Diels–Alder reactions [29–31].

**Scheme 1:**
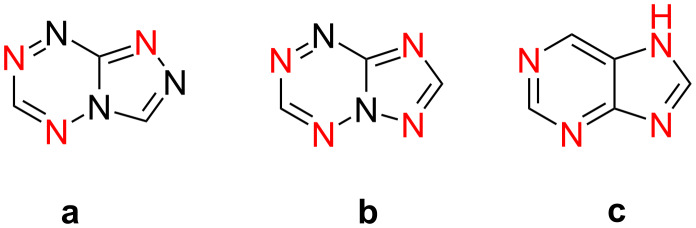
Chemical structures of [1,2,4]triazolo[4,3-*b*][1,2,4,5]tetrazine (**a**), [1,2,4]triazolo[1,5-*b*][1,2,4,5]tetrazine (**b**), 7*H*-purine (**c**).

At the same time, azolo-annulated 1,2,4,5-tetrazines remain to be a scarcely studied class of compounds, mainly due to the lack of convenient methods for their synthesis and modification. For example, synthesis and properties of some [1,2,4]triazolo[4,3-*b*][1,2,4,5]tetrazine derivatives are described in the literature [15–16,32–33]. However isomeric [1,2,4]triazolo[1,5-*b*][1,2,4,5]tetrazines are currently unknown. It is worth noting that triazolo[1,5-*b*][1,2,4,5]tetrazines are of particular interest for further research of their biological activity since the locations of nitrogen atoms in these molecules are closer to purine derivatives than those in isomeric known triazolo[4,3-*b*][1,2,4,5]tetrazines (Scheme 1).

Therefore, the development of methods for the synthesis and modification of new triazolo[1,5-*b*][1,2,4,5]tetrazines can be considered as an attractive task for heterocyclic and medicinal chemistry.

## Results and Discussion

In this work, the synthesis of [1,2,4]triazolo[1,5-*b*][1,2,4,5]tetrazines has been carried out for the first time.

**Scheme 2:**
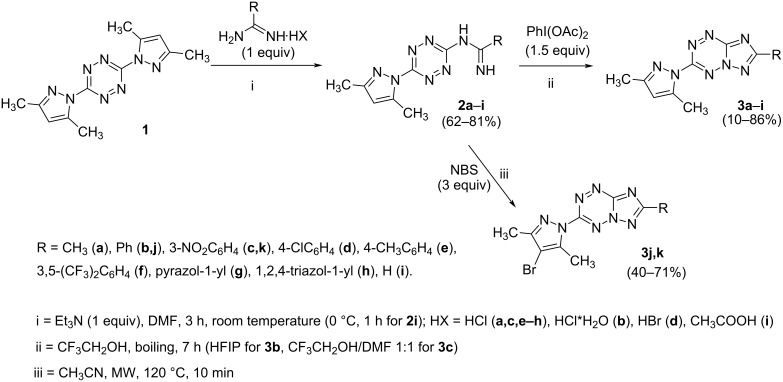
Synthesis of [1,2,4]triazolo[1,5-*b*][1,2,4,5]tetrazines **3a**–**k**.

The synthetic approach to new triazolotetrazines involves the preparation of 3,6-disubstituted 1,2,4,5-tetrazines **2a**–**i** bearing the amidine moiety through nucleophilic substitution of the 3,5-dimethylpyrazol fragment in easily available compound **1**, followed by oxidative cyclization of products **2a**–**i** (Scheme 2).

**Figure 1:**
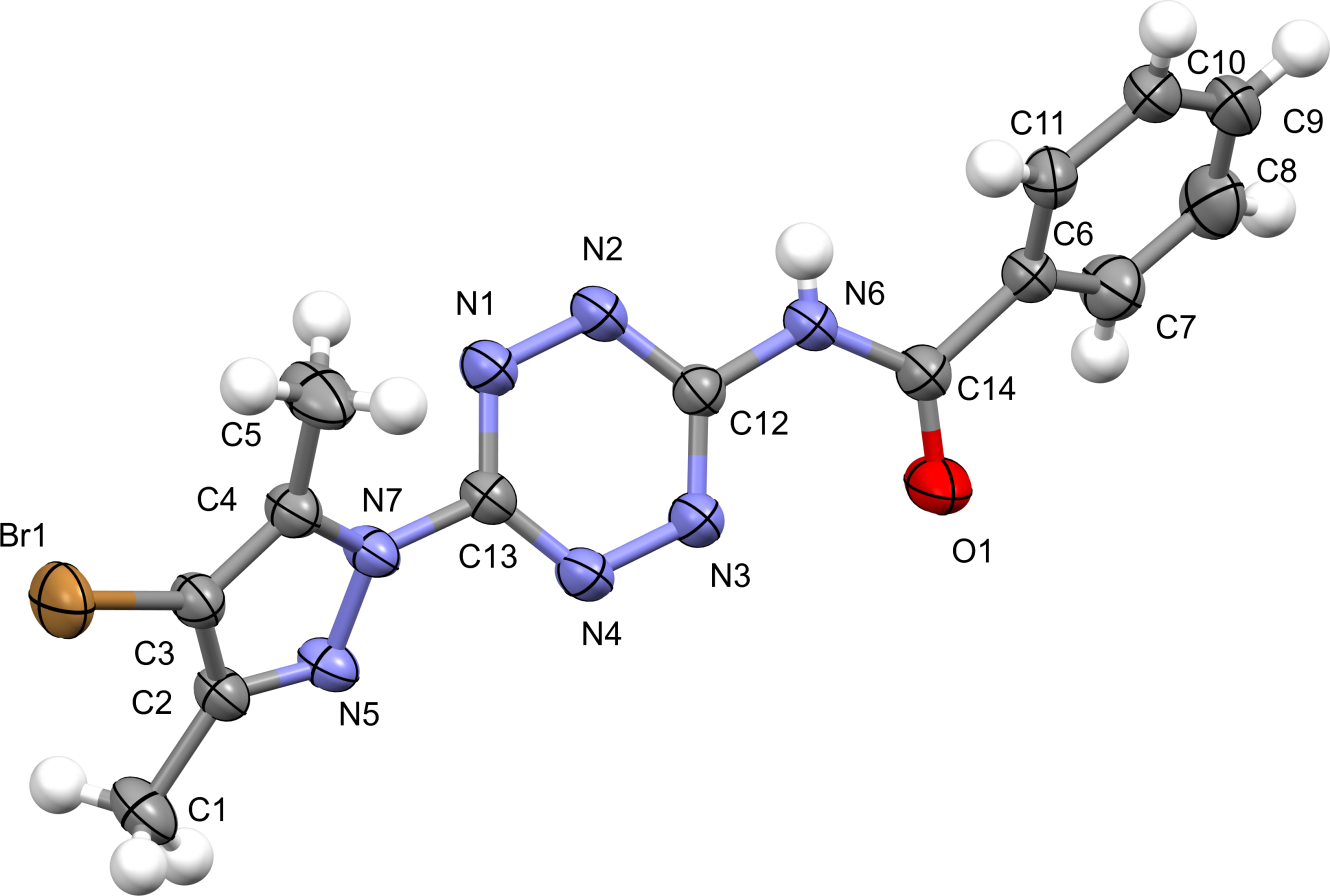
X-ray structure of *N*-(6-(4-bromo-3,5-dimethylpyrazol-1-yl)-1,2,4,5-tetrazin-3-yl)benzamide.

It has been shown that the oxidative cyclization of 1,2,4,5-tetrazines **2a**–**i** into triazolo[1,5-*b*][1,2,4,5]tetrazines **3a**–**i** takes place by action of (diacetoxyiodo)benzene on heating in trifluoroethanol. The target products were obtained in 10–86% yield. According to TLC data, the synthesis of derivatives containing 4-methylphenyl (**3e**), phenyl (**3b**) and 4-chlorophenyl (**3d**) substituents proved to be accompanied by the formation of a large number of byproducts of unknown structure, thus decreasing the yield to 15–30%. The yield of triazolotetrazine **3i** unsubstituted at C(7) was only 10%. It can be explained by a low stability of the starting tetrazine **2i** containing the formamidine moiety, since the latter on heating is easily transformed into the amino group, thus leading to 3-amino-6-(3,5-dimethylpyrazol-1-yl)-1,2,4,5-tetrazine. It has been found that *N*-bromosuccinimide can also be used as an oxidant for the oxidation of benzamidine derivatives **2b**,**c** in acetonitrile under microwave irradiation. Along with the cyclization reaction, bromination of the pyrazole ring proved to occur at position C(4) to give the products **3j**,**k**, as evidenced by disappearance of the characteristic singlet of H(4) of the pyrazolyl substituent in the region of 6.38–6.41 ppm in the ^1^H NMR spectrum. A small amount of *N*-(6-(4-bromo-3,5-dimethylpyrazol-1-yl)-1,2,4,5-tetrazin-3-yl)benzamide, as a byproduct, was isolated from the reaction mixture, the structure of the latter was confirmed by the XRD data (Figure 1).

In order to select optimal conditions for the synthesis of compounds **3**, the oxidative systems have been varied for the reactions of tetrazines **2a**,**b**,**g** bearing methyl, phenyl, and pyrazolyl substituents. Attempts to use Pb(OAc)_4_ as an oxidizing agent in CHCl_3_ resulted in the formation of difficult-to-separate reaction mixtures with a low content of the target products **3a**,**b**,**g**, which could not be isolated in a pure form. Heating of tetrazines **2** with *N*-bromosuccinimide in refluxing acetonitrile without microwave irradiation made it possible to obtain the product **3j** in a low yield (10%), but did not afford the products **3a**,**g**. The reactions of **2** with (diacetoxyiodo)benzene in acetonitrile gave the target products **3a**,**b**,**g**, however, both yields and purities of these compounds proved to be higher when trifluoroethanol was used as a solvent.

**Scheme 3:**
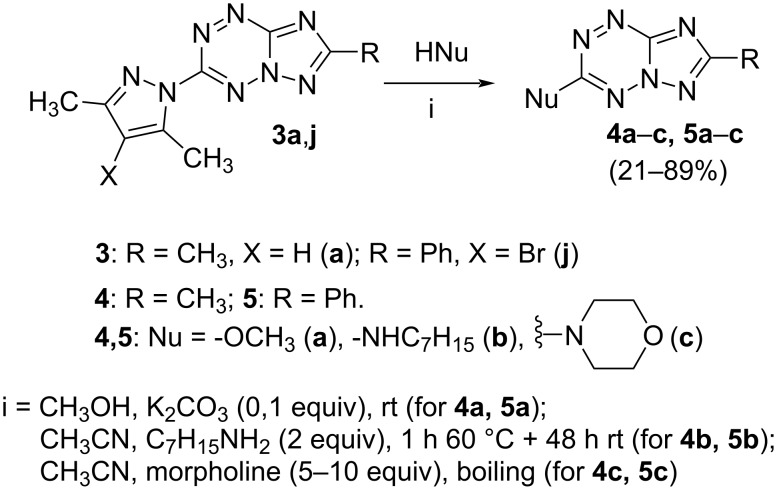
Reactions of triazolo[1,5-*b*][1,2,4,5]tetrazines **3a**,**j** with N- and O-nucleophiles.

It has been shown that new triazolo[1,5-*b*][1,2,4,5]tetrazines **3a**,**j**, as well as triazolo[4,3-*b*][1,2,4,5]tetrazines [33–35], react with N- and O-nucleophiles to give the corresponding nucleophilic substitution products **4a**–**c**, **5a**–**c**, derived from the displacement of the azolyl group in the tetrazine ring (Scheme 3). These reactions can be used to modify the triazolo[1,5-*b*][1,2,4,5]tetrazine ring system with a variety of structural fragments, thus enabling to vary their biological activity.

**Scheme 4:**
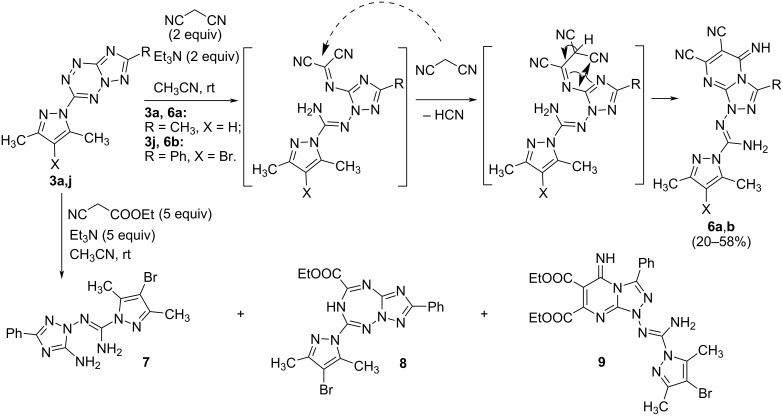
Reactions of triazolo[1,5-*b*][1,2,4,5]tetrazines **3a,j** with C-nucleophiles.

The reactions of new triazolo[1,5-*b*][1,2,4,5]tetrazines with some CH-active compounds, such as malononitrile, ethyl cyanoacetate and diethyl malonate, have been studied. Similarly to isomeric triazolo[4,3-*b*][1,2,4,5]tetrazines [36], these derivatives do not form *ipso*-substitution products with the displacement of a leaving group in the tetrazine ring by the action of CH-acids. Instead, a nucleophile attack on the nitrogen atom of the tetrazine ring does occur followed by ring-opening and ring closure (Scheme 4).

It has been found that triazolo[1,5-*b*][1,2,4,5]tetrazines **3** do not enter the characteristic for isomeric [4,3-*b*]-annulated derivative reactions with malonic ester, leading to expansion of the tetrazine ring and the formation of triazolotetrazepines [36]. At the same time, a more active malononitrile reacts easily with compounds **3a**,**j** to afford triazolopyrimidines **6a**,**b** (Scheme 4). In the reaction of ethyl cyanoacetate with triazolotetrazine **3j**, a mixture of products is formed, in which, along with the diamino compound **7** and triazolotetrazepine **8** (which is characteristic for the behavior of [4,3-*b*]-annulated systems) triazolopyrimidine **9** has been obtained (Scheme 4).

Product **9**, like products **6a**,**b**, appears to be derived from double addition of the reagent, accompanied by opening of the tetrazine ring and recyclization (Scheme 4). In the reaction of 7-methyl-substituted triazolotetrazine **3a** with ethyl cyanoacetate, a rather complicated mixture of several products has been obtained, none of the latter failed to be isolated in a pure form. Thus, it has been shown that new triazolo[1,5-*b*][1,2,4,5]tetrazines retain electrophilic centers inherent in the known triazolo[4,3-*b*][1,2,4,5]tetrazine system. However, their reactions have a low regioselectivity, thus leading to a large number of byproducts, thus making it difficult to separate the reaction mixture.

All synthesized [1,2,4]triazolo[1,5-*b*][1,2,4,5]tetrazines **3**–**5** were screened in vitro for their fungistatic activities against mycelial anthropophilic and zooanthropophilic dermatophytes (*Trichophyton*, *Microsporum* and *Epidermophyton*), which cause diseases of the skin and its appendages (hair, nails) (Table 1). In the series of compounds **3a**–**i**, in which substituents in the triazole ring were varied, the derivatives **3a,b,g**, containing methyl, phenyl and pyrazol-1-yl, proved to exhibit the highest activity. Triazolotetrazine **3a** shows a high activity (MIC ≤ 3.12 μg/mL) against the strains *Trichophyton tonsurans*, *Trichophyton violaceum* and *Epidermophyton floccosum*. Phenyl-substituted derivative **3b** proved to be a moderately active (MIC = 6.25 μg/mL) in relation to *Trichophyton violaceum* and *Epidermophyton floccosum*. In case of pyrazolyl derivative **3g**, a high fungistatic activity (MIC = 1.5 μg/mL) was detected for the *Epidermophyton floccosum* strain. Modification of active compounds **3a,b** at C(3) allowed us to obtain 5 more derivatives exhibiting a high and moderate activity (MIC from 0.38 to 6.25 μg/mL) for one (**3j**, **5b**) or several (**4a**, **5a**, **5c**) strains (Table. 1). Thus, the most promising compounds for chemical modifications aimed at the development of new fungistatics appear to be [1,2,4]triazolo[1,5-*b*][1,2,4,5]tetrazines, containing a methyl, phenyl or pyrazolyl substituent in the position C(7). It has been revealed that the introduction of methyl or methoxy groups into the structure of triazolotetrazines leads to a significant increase of activity for a wide range of strains (compounds **3a**, **4a**, **5a**). Introduction of alkylamino or 4-bromopyrazolyl substituents results in active compounds acting selectively on a particular dermatophyte strain. Indeed, compounds **3j** and **5b** proved to be effective against *Epidermophyton floccosum*, and **5c** against *Microsporum canis*.

To estimate the effect of [1,2,4]triazolo[1,5-*b*][1,2,4,5]tetrazine fragment on antifungal activity of the synthesized compounds, we have tested the activity of their analogues, unannulated 1,2,4,5-tetrazines **2c,g**, containing the amidine moiety, and isomeric [1,2,4]triazolo[4,3-*b*][1,2,4,5]tetrazines.

**Scheme 5:**
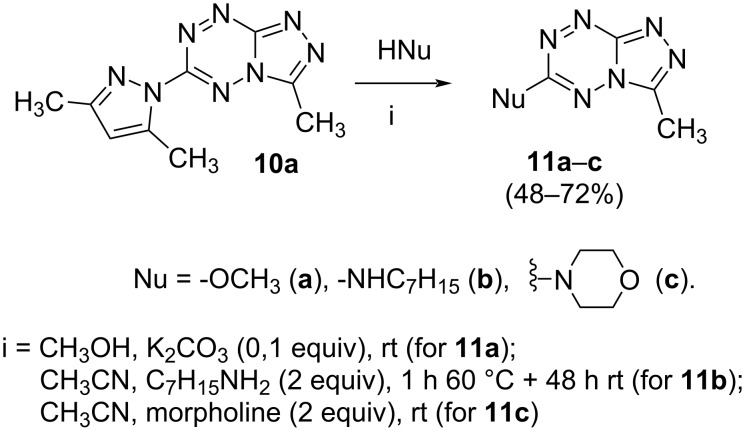
Reactions of 6-(3,5-dimethyl-1*H*-pyrazol-1-yl)-3-methyl-[1,2,4]triazolo[4,3-*b*][1,2,4,5]tetrazine (**10a**) with nucleophiles.

For a comparative analysis of antifungal activity, the previously described [37] 3-methyl- and 3-phenyltriazolo[4,3-*b*][1,2,4,5]tetrazines **10a,b** were used as structural analogues of compounds **3a,b**. Furthermore, in the reactions of compound **10a** with methanol, heptylamine and morpholine, new derivatives **11a**–**c** have been obtained, which can be regarded as isomers of compounds **4a**–**c** (Scheme 5).

Analysis of the data on fungistatic activity has revealed that in the series of compounds **2**, **10** and **11** no derivatives exhibit a pronounced activity (Table 1).

**Table 1:** In vitro fungistatic activity of [1,2,4]triazolo[1,5-*b*][1,2,4,5]tetrazines **3a**–**k**, **4a**–**c**, **5a**–**c** and their analogues.

Compd.^a^	Structure	Fungistatic activity, fungi type/ MIC, μg/mL						

		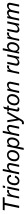	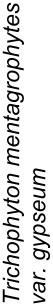	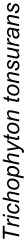	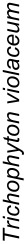	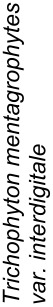	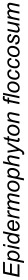	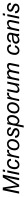

**2g**	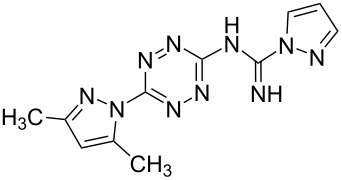	12.5	50	25	25	50	25	50
**3a**	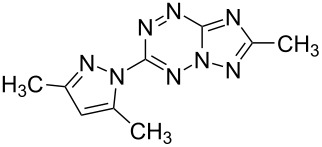	**6.25**	**6.25**	**1.5**	**1.5**	**6.25**	**3.12**	12.5
**3b**	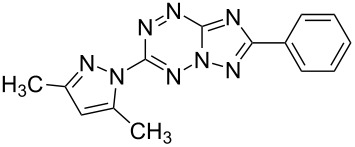	12.5	12.5	12.5	**6.25**	25	**6.25**	25
**3e**	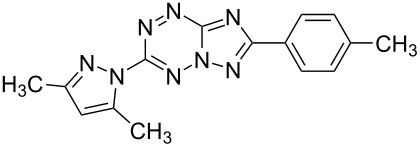	>100	>100	>100	>100	>100	>100	25
**3g**	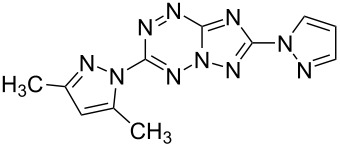	12.5	25	50	12.5	25	**1.5**	25
**3h**	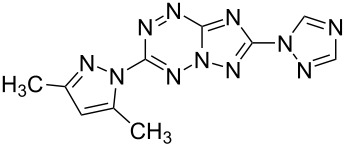	100	100	100	100	100	50	>100
**3i**	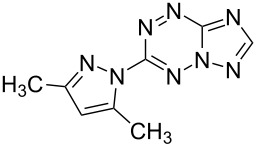	12.5	25	25	12.5	25	25	12.5
**3j**	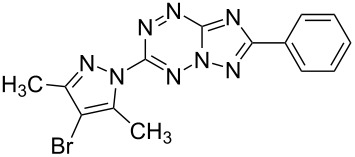	>100	>100	>100	>100	>100	**0.38**	>100
**4a**	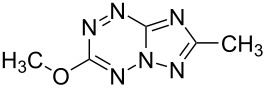	**6.25**	25	12.5	**1.5**	12.5	12.5	**6.25**
**4b**	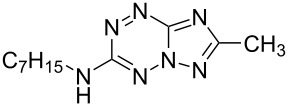	12.5	50	>100	12.5	12.5	25	12.5
**4c**	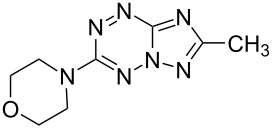	50	>100	100	100	>100	>100	50
**5a**	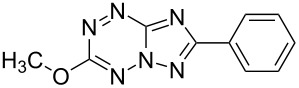	**3.12**	12.5	**6.25**	**6.25**	**3.12**	**1.5**	**3.12**
**5b**	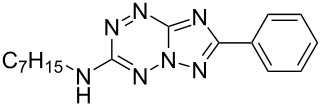	>100	>100	>100	>100	>100	**0.75**	>100
**5c**	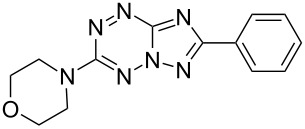	>100	>100	>100	**3.12**	>100	25	**1.5**
**10b**	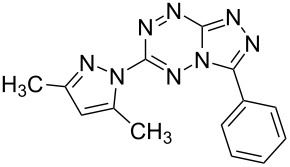	12.5	100	12.5	>100	12.5	–	–
**11a**	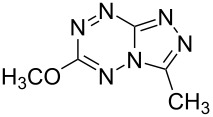	100	>100	100	50	>100	>100	>100
**11b**	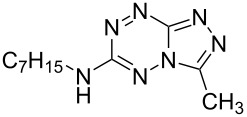	25	25	50	25	50	100	50
	fluconazole	6.25	12.5	>100	100	>100	3.12	100

^a^Compounds **2c**, **3c**, **3d**, **3f**, **3k**, **10a**, **11c** have a MIC ≥ 100 μg/mL.

## Conclusion

Thus, a new method for the synthesis of [1,2,4]triazolo[1,5-*b*][1,2,4,5]tetrazines has been developed. A comparative analysis of their reactivity and fungistatic activity relative to isomeric [1,2,4]triazolo[4,3-*b*][1,2,4,5]tetrazines has been performed. It has been shown that new derivatives retain the electrophilic character and the reactivity pattern in the reactions with nucleophiles known for annulated tetrazine systems. It has been revealed that the synthesized compounds exhibit a pronounced activity against dermatophyte fungal strains *Trichophyton*, *Epidermophyton*, *Microsporum*: Seven compounds with a high fungistatic activity (0.38 ≤ MIC ≤ 1.5 μg/mL) have been found. All the above-mentioned results demonstrate good prospects for finding new antifungal drugs in this class of compounds.

## Supporting Information

File 1 Experimental part.
