# Supplementary material for: Synthesis of novel [1,2,4]triazolo[1,5-b][1,2,4,5]tetrazines and investigation of their fungistatic activity
Source: Beilstein J Org Chem. 2022 Mar 1;18:243–50. doi: 10.3762/bjoc.18.29 (PMC8919425; doi:10.3762/bjoc.18.29)
Supplement: File 1 — Experimental part. [file Beilstein_J_Org_Chem-18-243-s001.pdf]

BEILSTEIN JOURNAL OF ORGANIC CHEMISTRY

Supporting Information

for

Synthesis of novel [1,2,4]triazolo[1,5-*b*][1,2,4,5]tetrazines and investigation of their fungistatic activity

Anna V. Korotina, Svetlana G. Tolshchina, Rashida I. Ishmetova, Natalya P. Evstigneeva, Natalya A. Gerasimova, Natalya V. Zilberberg, Nikolay V. Kungurov, Gennady L. Rusinov, Oleg N. Chupakhin and Valery N. Charushin

Beilstein J. Org. Chem. **2022,** *18,* 243–250. doi:10.3762/bjoc.18.29

Experimental part

License and Terms: This is a supporting information file under the terms of the Creative Commons Attribution License (https://creativecommons.org/licenses/by/4.0). Please note that the reuse, redistribution and reproduction in particular requires that the author(s) and source are credited and that individual graphics may be subject to special legal provisions.
The license is subject to the *Beilstein Journal of Organic Chemistry* terms and conditions: (https://www.beilstein-journals.org/bjoc/terms)

1. General

Synthesis and characteristics of compound **1** are described in the work [1], and of compound **10a**,**b** are described in the work [2].

All solvents were of analytical reagent (AR) grade and used as received. All new compounds were characterized by standard spectroscopical methods. ^{1}H and ^{13}C NMR spectra were recorded on a Bruker Avance DRX-400 and Bruker Avance-500 spectrometers at 400/500 and 100/125 MHz respectively using $CDCl_3$ and DMSO-d_6 as solvent and tetramethylsilane as internal standard. Chemical shifts are reported in ppm downfield from the standard ($\delta = 0.00$), coupling constants in Hz. Elemental analyses were performed on Perkin-Elmer PE 2400 analyser. Melting points were determined on Boetius aparatus. Flash chromatography was carried out using silica gel 60 (230–400 mesh). Thin layer chromatography was carried out on Sorbfil HPTLC-A-UV plates. Detection was done under UV light (254 nm and 365 nm). For the synthesis of compounds **3j**,**k**, the CEM Discover microwave chemical reactor (frequency 2.45 Hz, power 300 W) was used. High resolution mass spectra (HRMS) were recorded on a Bruker maXis impact HD quadrupole TOF mass spectrometer.

X-ray diffraction analysis of single crystals of *N*-(6-(4-bromo-3,5-dimethylpyrazol -1-yl)-1,2,4,5-tetrazin-3-yl)benzamide was performed on an Xcalibur S four-circle automated diffractometer with CCD detector. Analysis was carried out on standard procedure (graphite monochromated Mo K-irradiation, T = 295(2) K, ω-scanning). Solution and refinement of the structure was accomplished with using the SHELXS-97 [3] program package using the full-matrix least-squares method on F^2, in anisotropic approximation for non-hydrogen atoms. H-atoms are placed in calculated positions and refined in isotropic approximation in the “riding” model. Crystal size 0.18 × 0.11 × 0.03. On the angles $2.62 < \theta < 28.28^{o}$ 7196 reflections were collected, 3814 independent reflections (R_{int} = 0.0262), 2396 reflections with

I > 2σ(I). μ = 2.692 mm^{-1}. Crystal system is monoclinic, space group C2/*c*, *a* = 27.7149(18) Å, *b* = 7.3696(6) Å, *c* = 16.0211(10) Å, β = 109.855(6)°, *V* = 3077.8(4) $Å^3$, formula $C_{14}H_{13}BrN_7O_1{*}0.5H_2O$ (*Z* = 8). Final refinement parameters: R_1 = 0.0857, wR_2 = 0.1268 (all data), R_1 = 0.0429, wR_2 = 0.1022 (I > 2σ(I)), Goodness-of-fit 1.002. Maximum/minimum of residual electronic density $\Delta\rho_{\bar{e}}$ = 0.319/−0.460 $\bar{e}Å^{-3}$. The X-ray crystallography data for the structure have been deposited with the Cambridge Crystallography Data Centre as supplementary publication no CCDC 1937017. These data can be obtained free of charge from the Cambridge Crystallographic Data Centre via www.ccdc.cam.ac.uk/data_request/cif.

2. Antifungal activity

Studies of antifungal activity were carried out according to a procedure described earlier [4,5] using museum strains of fungi *Trichophyton rubrum*, *Trichophyton mentagrophytes var. gypseum*, *Trichophyton tonsurans*, *Trichophiton violaceum*, *Trichophyton mentagrophytes var. interdigitale*, *Epidermophyton floccosum*, *Microsporum canis* from Russian Collection of Pathogenic Fungi (P. N. Kashkin Research Institute of Medicinal Micology, St.Petersburg).

Fungi were cultivated at 27 °C on Sabouraud Dextrose Agar (SDA). The minimal inhibiting concentration (MIC) was determined using a serial dilution method in Sabouraud Dextrose Broth (SDB). The tested compounds and standard were dissolved in dimethylsulfoxide (DMSO, 1/10) (the starting concentration is 1000 μg/mL), diluted with SDB and applied in different concentrations from 100 till 0.19 μg/mL. Dimethylsulfoxide was used as a negative control and antifungal (fluconazole) as positive controls. The broth dilution test was performed in test tubes. The inoculum suspension, which gave the final concentration of 1×10^8 CFU/ml, was prepared. A growth control tube and sterility control tube were used in each test. After 14 days incubation at 27 °C, the MIC was determined visually as the lowest concentration that inhibits growth, evidenced by the absence of turbidity.

3. Chemistry

General procedure for the synthesis of *N*-(6-(3,5-dimethyl-1*H*-pyrazol-1-yl)-1,2,4,5-tetrazin-3-yl)imidamides 2a–i.

To 270 mg (1 mmol) of tetrazine **1** in 5 mL of DMF 1 mmol of amidine hydrochloride (in the case of synthesis **2b** - benzamidine hydrochloride hydrate, **2d** - 4-chlorobenzamidine hydrobromide, **2i** - formamidine acetate) and 101 mg (1 mmol) Et_3N were added. The reaction was stirred for 3 hours at room temperature (**2i** was stirred while cooling on ice for 1 hour, then kept at room temperature for a day). The reaction mixture was diluted with 10 mL of water, filtered, and washed with CH_2Cl_2.

***N*-(6-(3,5-Dimethyl-1*H*-pyrazol-1-yl)-1,2,4,5-tetrazin-3-yl)acetimidamide (2a).** Red solid, 81%, m.p. 133-134 °C (decomp.); ^{1}H NMR (DMSO-d6, 400MHz): δ/ppm 2.19, 2.24, 2.50 (all s, 9H, 3CH_3), 6.25 (s, 1H, CH, 3,5-dimethylpyrazolyl), 8.21, 8.29 (both br s, 2H, 2NH); ^{13}C NMR (DMSO-d6, 125MHz): δ/ppm 12.9, 13.4, 21.2, 109.4, 141.9, 150.9, 156.6, 165.7, 166.2. Anal. Calcd for $C_9H_{12}N_8$: C, 46.54; H, 5.21; N, 48.25; Found C, 46.49; H, 5.08; N, 48.18. LC/MS (ESI): Found *m/z* = 233.1261 (M+H). $C_9H_{13}N_8^+$. Calculated 233.1258 (M+H).

***N*-(6-(3,5-Dimethyl-1*H*-pyrazol-1-yl)-1,2,4,5-tetrazin-3-yl)benzimidamide (2b).** Red solid, 77%, m.p. 238-239 °C; ^{1}H NMR (DMSO-d6, 500MHz): δ/ppm 2.27, 2.53 (both s, 6H, 2CH_3), 6.28 (s, 1H, CH, 3,5-dimethylpyrazolyl), 7.53-7.59, 8.10-8.14 (both m, 4H, Ph), 7.59-7.65 (m, 1H, Ph), 8.70, 8.88 (both br s, 2H, 2NH); ^{13}C NMR (DMSO-d6, 125MHz): δ/ppm 13.1, 13.4, 109.7, 127.9, 128.5, 131.7, 134.4, 142.1, 151.2, 156.4, 161.6, 166.9. Anal. Calcd for $C_{14}H_{14}N_8$: C, 57.13; H, 4.79; N, 38.07; Found C, 57.17; H, 4.76; N, 38.19.

***N*-(6-(3,5-Dimethyl-1*H*-pyrazol-1-yl)-1,2,4,5-tetrazin-3-yl)-3-nitrobenzimidamide (2c).** Red solid, 81%, m.p. > 295 °C (decomp.); ^{1}H NMR (DMSO-d6, 500MHz): δ/ppm 2.27, 2.55 (both s, 6H, 2CH_3), 6.30 (s, 1H, CH, 3,5-dimethylpyrazolyl), 7.88 (dd, J_1=J_2=7.9Hz, 1H, Ar), 8.47, 8.58 (both d, J=7.9Hz,

2H, Ar) 8.85, 9.19 (both br s, 2H, 2NH), 8.98 (s, 1H, Ar); ^{13}C NMR (DMSO-d6, 125MHz): *δ*/ppm 13.2, 13.4, 109.9, 122.7, 126.3, 130.3, 134.1, 136.0, 142.3, 147.9, 151.4, 156.6, 159.2, 166.7. Anal. Calcd for $C_{14}H_{13}N_9O_2$: C, 49.56; H, 3.86; N, 37.15; Found C, 49.30; H, 3.78; N, 37.26.

***N*-(6-(3,5-Dimethyl-1*H*-pyrazol-1-yl)-1,2,4,5-tetrazin-3-yl)-4-chlorobenzimidamide (2d).**

Red solid, 67%, m.p. 283-284 °C; ^{1}H NMR (DMSO-d6, 400MHz): *δ*/ppm 2.27, 2.54 (both s, 6H, $2CH_3$), 6.29 (s, 1H, CH, 3,5-dimethylpyrazolyl), 7.64, 8.15 (both d, *J*=8.6Hz, 4H, Ar), 8.73, 8.92 (both br s, 2H, 2NH); ^{13}C NMR (DMSO-d6, 100MHz): *δ*/ppm 13.1, 13.4, 109.8, 128.6, 129.8, 133.2, 136.6, 139.7, 142.2, 151.2, 160.4, 166.8. Anal. Calcd for $C_{14}H_{13}ClN_8$: C, 51.15; H, 3.99; N, 34.08; Found C, 51.22; H, 4.03; N, 34.05.

***N*-(6-(3,5-Dimethyl-1*H*-pyrazol-1-yl)-1,2,4,5-tetrazin-3-yl)-4-methylbenzimidamide (2e).**

Red solid, 81%, m.p. 257-259 °C; ^{1}H NMR (DMSO-d6, 500MHz): *δ*/ppm 2.26, 2.41, 2.53 (all s, 9H, $3CH_3$), 6.28 (s, 1H, CH, 3,5-dimethylpyrazolyl), 7.37, 8.03 (both d, *J*=8.1Hz, 4H, Ar), 8.69, 8.84 (both br s, 2H, 2NH); ^{13}C NMR (DMSO-d6, 100MHz): *δ*/ppm 13.0, 13.4, 21.0, 109.7, 127.9, 129.0, 131.5, 141.9, 142.1, 151.1, 156.3, 161.5, 166.8. Anal. Calcd for $C_{15}H_{16}N_8$: C, 58.43; H, 5.23; N, 36.34; Found C, 58.63; H, 5.16; N, 36.54.

***N*-(6-(3,5-Dimethyl-1*H*-pyrazol-1-yl)-1,2,4,5-tetrazin-3-yl)-3,5-bis(trifluoromethyl)benzimidamide (2f).**

Red solid, 65%, m.p. 233-235 °C; ^{1}H NMR (DMSO-d6, 500MHz): *δ*/ppm 2.28, 2.56 (both s, 6H, $2CH_3$), 6.31 (s, 1H, CH, 3,5-dimethylpyrazolyl), 8.42 (s, 1H, Ar), 8.81 (s, 2H, 2CH), 8.95, 9.32 (both br s, 2H, 2NH); ^{13}C NMR (DMSO-d6, 125MHz): *δ*/ppm 13.2, 13.4, 110.0, 123.1 (q, *J*=273Гц), 125.3, 128.6, 130.6 (q, *J*=33Гц), 136.8,

142.3, 151.5, 156.6, 158.1, 166.6. Anal. Calcd for $C_{16}H_{12}F_6N_8$: C, 44.66; H, 2.81; N, 26.04; Found C, 44.70; H, 3.10; N, 26.22.

***N*-(6-(3,5-Dimethyl-1*H*-pyrazol-1-yl)-1,2,4,5-tetrazin-3-yl)-1*H*-pyrazole-1-carboximidamide (2g).**

Red solid, 81%, m.p. 198-200 °C; ^{1}H NMR (DMSO-d6, 500MHz): δ/ppm 2.28, 2.54 (both s, 6H, $2CH_3$), 6.30 (s, 1H, CH, 3,5-dimethylpyrazolyl), 6.66-6.70, 7.95-7.99 (both m, 2H, Ar), 8.68 (d, *J*=2.7Hz, 1H, Ar) 8.68, 8.98 (both br s, 2H, 2NH); ^{13}C NMR (DMSO-d6, 125MHz): δ/ppm 13.1, 13.4, 109.7, 109.9, 129.4, 142.3, 143.6, 149.8, 151.4, 156.6, 165.9. Anal. Calcd for $C_{11}H_{12}N_{10}$: C, 46.47; H, 4.25; N, 49.27; Found C, 46.36; H, 4.15; N, 49.37. LC/MS (ESI): Found *m/z* = 285.1324 (M+H). $C_{11}H_{13}N_{10}^+$. Calculated 285.1319 (M+H).

***N*-(6-(3,5-Dimethyl-1*H*-pyrazol-1-yl)-1,2,4,5-tetrazin-3-yl)-1*H*-1,2,4-triazole-1-carboximidamide (2h).**

Red solid, 67%, m.p. 220-222 °C; ^{1}H NMR (DMSO-d6, 500MHz): δ/ppm 2.27, 2.56 (both s, 6H, $2CH_3$), 6.31 (s, 1H, CH, 3,5-dimethylpyrazolyl), 8.43, 9.49 (both s, 2H, 2CH, Ar), 8.78, 9.11 (both br s, 2H, 2NH); ^{13}C NMR (DMSO-d6, 125MHz): δ/ppm 13.2, 13.4, 110.1, 142.4, 144.5, 148.1, 151.6, 153.2, 156.9, 165.6. Anal. Calcd for $C_{10}H_{11}N_{11}$: C, 42.10; H, 3.89; N, 54.01; Found C, 41.95; H, 4.09; N, 54.13.

***N*-(6-(3,5-Dimethyl-1*H*-pyrazol-1-yl)-1,2,4,5-tetrazin-3-yl)formimidamide (2i).**

Red solid, 62%, m.p. 245-247 °C; ^{1}H NMR (DMSO-d6, 400MHz): δ/ppm 2.25, 2.49 (both s, 6H, $2CH_3$), 6.24 (s, 1H, CH, 3,5-dimethylpyrazolyl), 8.20, 8.63 (both br s, 2H, 2NH), 8.54 (s, 1H, CH); ^{13}C NMR (DMSO-d6, 100MHz): δ/ppm 12.7, 13.3, 109.3, 141.9, 150.8, 158.3, 158.8, 166.8. Anal. Calcd for $C_8H_{10}N_8$: C, 44.03; H, 4.62; N, 51.35; Found C, 44.05; H, 4.92; N, 51.27.

General procedure for the synthesis of 3-(3,5-dimethyl-1*H*-pyrazol-1-yl)-7-R-[1,2,4]triazolo[1,5-*b*][1,2,4,5]tetrazines 3a–i.

To 1 mmol of *N*-(6-(3,5-dimethyl-1*H*-pyrazol-1-yl)-1,2,4,5-tetrazin-3-yl) imidamide **2** in 4 mL of trifluoroethanol by heating (74 ° C) (for the compound **3b**, hexafluoroisopropanol 4 mL at 55 °C was used as a solvent, **3c** – trifluoroethanol + DMF (2 mL + 2 mL)) 1.5 mmol (483 mg) $PhI(OAc)_2$ was added in portions, stirred for 7 hours, then the solvent was evaporated, the residue was washed with EtOH and CH_2Cl_2 (compounds **3e**, **3i** were purified by column chromatography, eluent EtOAc/hexane, 1: 1 (**3e**) and benzene/CH_3CN, 1:1 (**3i**); **3b**: The residue was dissolved in DMF and precipitated with water), the precipitate was filtered.

3-(3,5-Dimethyl-1*H*-pyrazol-1-yl)-7-methyl-[1,2,4]triazolo[1,5-*b*][1,2,4,5]tetrazine (3a).

Yellow solid, 74%, m.p. 180-182 °C; ^{1}H NMR (DMSO-d6, 500MHz): δ/ppm 2.28, 2.58, 2.76 (all s, 9H, $3CH_3$), 6.35 (s, 1H, CH, 3,5-dimethylpyrazolyl); ^{13}C NMR (DMSO-d6, 125MHz): δ/ppm 13.3, 13.4, 15.4, 110.6, 143.1, 151.5, 152.2, 152.3, 167.7. Anal. Calcd for $C_9H_{10}N_8$: C, 46.95; H, 4.38; N, 48.67; Found C, 46.90; H, 4.38; N, 48.46. LC/MS (ESI): Found m/z = 231.1104 (M+H). $C_9H_{11}N_8^+$. Calculated 231.1101 (M+H).

3-(3,5-Dimethyl-1*H*-pyrazol-1-yl)-7-phenyl-[1,2,4]triazolo[1,5-*b*][1,2,4,5]tetrazine (3b).

Yellow solid, 33%, m.p. 193-194 °C; ^{1}H NMR (DMSO-d6, 500MHz): δ/ppm 2.30, 2.62 (both s, 6H, $2CH_3$), 6.38 (s, 1H, CH, 3,5-dimethylpyrazolyl), 7.64-7.74 (m, 3H, Ph), 8.30-8.47 (m, 2H, Ph); ^{13}C NMR (DMSO-d6, 125MHz): δ/ppm 13.4, 110.8, 127.6, 128.5, 129.5, 132.5, 143.1, 152.4, 165.5. Anal. Calcd for $C_{14}H_{12}N_8$: C, 57.53; H, 4.14; N, 38.34; Found C, 57.48; H, 3.97; N, 38.05.

3-(3,5-Dimethyl-1*H*-pyrazol-1-yl)-7-(3-nitrophenyl)-[1,2,4]triazolo[1,5-*b*][1,2,4,5]tetrazine (3c).

Yellow solid, 68%, m.p. 238-239 °C; ^{1}H NMR (DMSO-d6, 500MHz): δ/ppm 2.31, 2.64 (both s, 6H, $2CH_3$), 6.41 (s, 1H, CH, 3,5-dimethylpyrazolyl), 7.99 (dd, J_1=J_2=8.0Hz, 1H, Ar), 8.53, 8.80 (both d, J=8.0Hz, 2H, Ar) 9.05 (s, 1H, Ar); ^{13}C NMR (DMSO-d6, 125MHz): δ/ppm 13.4, 13.5, 111.1, 121.8, 126.8, 130.0, 131.5, 133.5, 143.3, 148.6, 152.1, 152.7, 152.8, 163.2. Anal. Calcd for $C_{14}H_{11}N_9O_2$: C, 49.85; H, 3.29; N, 37.37; Found C, 49.52; H, 3.44; N, 37.13.

7-(4-Chlorophenyl)-3-(3,5-dimethyl-1*H*-pyrazol-1-yl)-[1,2,4]triazolo[1,5-*b*][1,2,4,5]tetrazine (3d).

Yellow solid, 35%, m.p. 252-253 °C; ^{1}H NMR (DMSO-d6, 500MHz): δ/ppm 2.30, 2.62 (both s, 6H, $2CH_3$), 6.38 (s, 1H, CH, 3,5-dimethylpyrazolyl), 7.75, 8.39 (both d, J=8.1Hz, 4H, Ar); ^{13}C NMR (DMSO-d6, 125MHz): δ/ppm 13.4, 110.8, 127.4, 129.3, 129.7, 137.3, 143.1, 152.1, 152.5, 164.4. Anal. Calcd for $C_{14}H_{11}ClN_8$: C, 51.46; H, 3.39; N, 34.29; Found C, 51.16; H, 3.51; N, 34.11.

3-(3,5-Dimethyl-1*H*-pyrazol-1-yl)-7-*p*-tolyl-[1,2,4]triazolo[1,5-*b*][1,2,4,5]tetrazine (3e).

Yellow solid, 15%, m.p. 200-202 °C; ^{1}H NMR (DMSO-d6, 400MHz): δ/ppm 2.31, 2.45, 2.63 (all s, 9H, $3CH_3$), 6.37 (s, 1H, CH, 3,5-dimethylpyrazolyl), 7.48, 8.28 (both d, J=8.1Hz, 4H, Ar); ^{13}C NMR (DMSO-d6, 100MHz): δ/ppm 13.4, 21.2, 110.7, 125.8, 127.6, 130.1, 142.8, 143.1, 152.2, 152.3, 152.5, 165.7. Anal. Calcd for $C_{15}H_{14}N_8$: C, 58.81; H, 4.61; N, 36.58; Found C, 58.70; H, 4.54; N, 36.45.

7-(3,5-Bis(trifluoromethyl)phenyl)-3-(3,5-dimethyl-1*H*-pyrazol-1-yl)-[1,2,4]triazolo[1,5-*b*][1,2,4,5]tetrazine (3f).

Yellow solid, 53%, m.p. 139-140 °C; ^{1}H NMR (DMSO-d6, 500MHz): δ/ppm 2.31(s, 3H, CH_3), 2.65 (br s, 3H, CH_3), 6.41 (s, 1H, CH, 3,5-dimethylpyrazolyl), 8.81 (br s, 3H, Ar); ^{13}C NMR (DMSO-d6, 125MHz): δ/ppm 13.9, 111.6, 123.4 (q, J=272Гц),

126.3, 128.1, 132.1, 143.8, 153.4. Anal. Calcd for $C_{16}H_{10}F_6N_8$: C, 44.87; H, 2.35; N, 26.61; Found C, 44.64; H, 2.32; N, 26.45.

3-(3,5-Dimethyl-1*H*-pyrazol-1-yl)-7-(1*H*-pyrazol-1-yl)-[1,2,4]triazolo[1,5-*b*][1,2,4,5]tetrazine (3g).

Yellow solid, 67%, m.p. 234-236 °C; ^{1}H NMR (DMSO-d6, 500MHz): δ/ppm 2.30, 2.62 (both s, 6H, $2CH_3$), 6.39 (s, 1H, CH, 3,5-dimethylpyrazolyl), 6.81, 8.10, 8.82 (all s, 3H, Ar); ^{13}C NMR (DMSO-d6, 125MHz): δ/ppm 13.4, 110.7, 110.9, 130.9, 143.1, 145.5, 151.3, 152.6, 152.9, 159.3. Anal. Calcd for $C_{11}H_{10}N_{10}$: C, 46.81; H, 3.57; N, 49.62; Found C, 47.07; H, 3.42; N, 49.38. LC/MS (ESI): Found *m/z* = 283.1168 (M+H). $C_{11}H_{11}N_{10}^+$. Calculated 283.1163 (M+H).

3-(3,5-Dimethyl-1*H*-pyrazol-1-yl)-7-(1*H*-1,2,4-triazol-1-yl)-[1,2,4]triazolo[1,5-*b*][1,2,4,5]tetrazine (3h).

Yellow solid, 86%, m.p. 245-247 °C; ^{1}H NMR (DMSO-d6, 500MHz): δ/ppm 2.31, 2.63 (both s, 6H, $2CH_3$), 6.42 (s, 1H, CH, 3,5-dimethylpyrazolyl), 8.57 (s, 1H, Ar), 9.78 (br s, 1H, Ar); ^{13}C NMR (DMSO-d6, 125MHz): δ/ppm 13.4, 13.4, 111.2, 143.3, 145.7, 150.9, 152.9, 153.1, 154.6, 157.0. Anal. Calcd for $C_{10}H_9N_{11}$: C, 42.40; H, 3.20; N, 54.39; Found C, 42.40; H, 3.14; N, 54.25.

3-(3,5-Dimethyl-1*H*-pyrazol-1-yl)-[1,2,4]triazolo[1,5-*b*][1,2,4,5]tetrazine (3i).

Yellow solid, 10%, m.p. 185-190 °C; ^{1}H NMR (DMSO-d6, 500MHz): δ/ppm 2.29, 2.61 (both s, 6H, $2CH_3$), 6.38 (s, 1H, CH, 3,5-dimethylpyrazolyl), 9.27 (s, 1H, C(7)H); ^{13}C NMR (DMSO-d6, 100MHz): δ/ppm 13.4, 110.9, 143.3, 150.9, 152.5, 152.6, 156.9. Anal. Calcd for $C_8H_8N_8$: C, 44.44; H, 3.73; N, 51.83; Found C, 44.33; H, 3.54; N, 51.61.

General procedure for the synthesis of 3-(4-bromo-3,5-dimethyl-1*H*-pyrazol-1-yl)-7-R-[1,2,4]triazolo[1,5-*b*][1,2,4,5]tetrazines 3j,k.

To 0.35 mmol of *N*-(6-(3,5-dimethyl-1*H*-pyrazol-1-yl)-1,2,4,5-tetrazin-3-yl) imidamide (**2b,c**) in 5 mL of acetonitrile with stirring was added 1.05 mmol (187 mg) of *N*-bromosuccinimide. The reaction was carried out under microwave irradiation at a temperature of 120 °C for 10 minutes. Then, during the synthesis of **3j**, the solvent was evaporated, the residue was recrystallized from ethanol, **3k** – the solvent was evaporated, washed with chloroform, filtered, the filtrate was evaporated, the residue was recrystallized from acetonitrile. The precipitate was filtered.

3-(4-Bromo-3,5-dimethyl-1*H*-pyrazol-1-yl)-7-phenyl-[1,2,4]triazolo[1,5-*b*][1,2,4,5]tetrazine (3j).

Yellow solid, 71%, m.p. 209-210 °C; ^{1}H NMR (DMSO-d6, 500MHz): δ/ppm 2.33, 2.63 (both s, 6H, 2CH_3), 7.65-7.72 (m, 3H, Ph), 8.37-8.43 (m, 2H, Ph); ^{13}C NMR (DMSO-d6, 125MHz): δ/ppm 12.4, 12.6, 99.9, 127.7, 128.4, 129.6, 132.7, 140.7, 150.9, 152.0, 152.5, 166.0. Anal. Calcd for $C_{14}H_{11}BrN_8$: C, 45.30; H, 2.99; N, 30.19; Found C, 45.29; H, 3.15; N, 30.24.

3-(4-Bromo-3,5-dimethyl-1*H*-pyrazol-1-yl)-7-(3-nitrophenyl)-[1,2,4]triazolo[1,5-*b*][1,2,4,5]tetrazine (3k).

Yellow solid, 40%, m.p. 239-240 °C; ^{1}H NMR (DMSO-d6, 400MHz): δ/ppm 2.35, 2.67 (both s, 6H, 2CH_3), 8.00 (dd, J_1=J_2=8.0Hz, 1H, Ar), 8.53 (dd, J_1=8.0Hz J_2=1.4Hz, 1H, Ar), 8.80 (d, J=8.0Hz, 1H, Ar) 9.06 (s, 1H, Ar); ^{13}C NMR (DMSO-d6, 100MHz): δ/ppm 12.8, 13.2, 100.7, 122.4, 127.3, 130.4, 132.0, 134.1, 141.4, 149.1, 151.8, 152.8, 152.8, 164.3. Anal. Calcd for $C_{14}H_{10}BrN_9O_2$: C, 40.40; H, 2.42; N, 30.29; Found C, 40.11; H, 2.47; N, 30.01.

General procedure for the synthesis of 3-methoxy-7-R-[1,2,4]triazolo[1,5-*b*][1,2,4,5]tetrazines 4a, 5a and 3-methoxy-6-methyl-[1,2,4]triazolo[4,3-*b*][1,2,4,5]tetrazine (11a).

To 1 mmol of 3-Het-7-R-[1,2,4]triazolo[1,5-*b*][1,2,4,5]tetrazine (**3a, 3j**) or 3-(3,5-dimethyl-1*H*-pyrazol-1-yl)-6-methyl-[1,2,4]triazolo[4,3-*b*][1,2,4,5]tetrazine (**10a**) in 5 mL of methanol 0.1 mmol (14 mg) of K_2CO_3 was added. The reaction mixture was stirred at room temperature.

3-Methoxy-7-methyl-[1,2,4]triazolo[1,5-*b*][1,2,4,5]tetrazine (4a).

Reaction time 1.5 hour. The substance was isolated by flash-chromatography (eluent benzene - acetonitrile, 1:1), the solvent was evaporated and the residue was washed with hexane and was filtered off. Yellow solid, 89%, m.p. 130-133 °C; ^{1}H NMR (DMSO-d6, 400MHz): δ/ppm 2.66 (s, 3H, CH_3), 4.16 (s, 3H, OCH_3). Anal. Calcd for $C_5H_6N_6O$: C, 36.15; H, 3.64; N, 50.58; Found C, 36.07; H, 3.63; N, 50.28. LC/MS (ESI): Found *m/z* = 167.0676 (M+H). $C_5H_7N_6O^+$. Calculated 167.0676 (M+H).

3-Methoxy-7-phenyl-[1,2,4]triazolo[1,5-*b*][1,2,4,5]tetrazine (5a). Reaction time 4 hour. The precipitate was filtered and recrystallized from acetonitrile. Yellow solid, 79%, m.p. 222-223 °C; ^{1}H NMR (DMSO-d6, 500MHz): δ/ppm 4.21 (s, 3H, OCH_3), 7.61-7.67 (m, 3H, Ph), 8.28-8.35 (m, 2H, Ph). Anal. Calcd for $C_{10}H_8N_6O$: C, 52.63; H, 3.53; N, 36.83; Found C, 52.56; H, 3.42; N, 36.74.

3-Methoxy-6-methyl-[1,2,4]triazolo[4,3-*b*][1,2,4,5]tetrazine (11a).

Reaction time 15 minutes. The substance was isolated by flash-chromatography (eluent benzene - acetonitrile, 1:1), the solvent was evaporated, the residue was washed with diethyl ether and was filtered off. Yellow solid, 66%, m.p. 120-122 °C; ^{1}H NMR (DMSO-d6, 400MHz): δ/ppm 2.71 (s, 3H, CH_3), 4.13 (s, 3H, OCH_3). Anal. Calcd for $C_5H_6N_6O$: C, 36.15; H, 3.64; N, 50.58; Found C, 36.26; H, 3.65; N, 50.61.

General procedure for the synthesis of 3-heptylamino-7-R-[1,2,4]triazolo[1,5-*b*][1,2,4,5]tetrazines 4b, 5b and 3-heptylamino-6-methyl-[1,2,4]triazolo[4,3-*b*][1,2,4,5]tetrazine (11b).

To 1 mmol of 3-((4-bromo-)3,5-dimethyl-1*H*-pyrazol-1-yl)-7-R-[1,2,4]triazolo[1,5-*b*][1,2,4,5]tetrazine (**3a, 3j**) or 3-(3,5-dimethyl-1*H*-pyrazol-1-yl)-6-methyl-[1,2,4]triazolo[4,3-*b*][1,2,4,5]tetrazine (**10a**) in 5 mL of acetonitrile 2 mmol (230 mg) of heptylamine was added, reaction mixture was stirred for 1 hour at 60 °C and two days at room temperature. The solvent was evaporated.

3-Heptylamino-7-methyl-[1,2,4]triazolo[1,5-*b*][1,2,4,5]tetrazine (4b).

The residue was dissolved in acetonitrile, the product was precipitated with water and was filtered off. Yellow solid, 48%, m.p. 83-84 °C; ^{1}H NMR (DMSO-d6, 400MHz): δ/ppm 0.86 (t, J=6.9 Hz, 3H, CH_3, $NH(CH_2)_6\underline{CH_3}$), 1.23-1.41 (m, 8H, $4CH_2$), 1.58-1.66 (m, 2H, CH_2), 2.52 (s, 3H, C(7)CH_3), 3.22-3.27 (m, 2H, CH_2), 8.70 (br s, 1H, NH). Anal. Calcd for $C_{11}H_{19}N_7$: C, 52.99; H, 7.68; N, 39.33; Found C, 53.13; H, 7.81; N, 39.37. LC/MS (ESI): Found *m/z* = 250.1777 (M+H). $C_{11}H_{20}N_7^+$. Calculated 250.1775 (M+H).

3-Heptylamino-7-phenyl-[1,2,4]triazolo[1,5-*b*][1,2,4,5]tetrazine (5b).

The substance was isolated by flash-chromatography (eluent benzene - acetonitrile, 1: 1), the solvent was evaporated, the residue was recrystallized from ethanol and was filtered off. Orange solid, 78%, m.p. 179-180 °C; ^{1}H NMR ($CDCl_3$, 500MHz): δ/ppm 0.90 (t, J=6.9 Hz, 3H, CH_3), 1.29-1.50 (m, 8H, $4CH_2$), 1.72-1.80 (m, 2H, CH_2), 3.47-3.56 (m, 2H, CH_2), 5.95 (br s, 1H, NH), 7.49-7.56 (m, 3H, Ph), 8.32-8.40 (m, 2H, Ph). Anal. Calcd for $C_{16}H_{21}N_7$: C, 61.72; H, 6.80; N, 31.49; Found C, 61.79; H, 6.85; N, 31.51.

3-Heptylamino-6-methyl-[1,2,4]triazolo[4,3-*b*][1,2,4,5]tetrazine (11b).

The residue was washed with diethyl ester and was filtered off. Yellow solid, 48%, m.p. 132-133 °C; ^{1}H NMR (DMSO-d6, 500MHz): δ/ppm 0.87 (t, J=6.9 Hz, 3H,

CH_3, NH$(CH_2)_6$<u>CH_3</u>), 1.22-1.41 (m, 8H, $4CH_2$), 1.61-1.68 (m, 2H, CH_2), 2.57 (s, 3H, C(6)CH_3), 3.15-3.29 (m, 2H, CH_2), 8.90 (br s, 1H, NH). Anal. Calcd for $C_{11}H_{19}N_7$: C, 52.99; H, 7.68; N, 39.33; Found C, 53.08; H, 7.57; N, 39.26.

7-Methyl-3-morpholino-[1,2,4]triazolo[1,5-*b*][1,2,4,5]tetrazine (4c).

To 1 mmol (230 mg) of 3-(3,5-dimethyl-1*H*-pyrazol-1-yl)-7-methyl-[1,2,4]triazolo[1,5-*b*][1,2,4,5]tetrazine (**3a**) in 5 mL of acetonitrile 5 mmol (435 mg) of morpholine were added, and reaction mixture was boiled for 12 hours. The solvent was evaporated. The substance was isolated by flash-chromatography (eluent benzene - acetonitrile, 1:1), the solvent was evaporated and the residue was washed with hexane and was filtered off. Orange solid, 21%, m.p. 118-120 °C; ^{1}H NMR ($CDCl_3$, 400MHz): δ/ppm 2.69 (s, 3H, CH_3), 3.87-3.90, 3.94-3.99 (both m, 8H, $4CH_2$). Anal. Calcd for $C_8H_{11}N_7O$: C, 43.43; H, 5.01; N, 44.32; Found C, 43.49; H, 5.10; N, 44.25. LC/MS (ESI): Found *m/z* = 222.1101 (M+H). $C_8H_{12}N_7O^+$. Calculated 222.1098 (M+H).

3-Morpholino-7-phenyl-[1,2,4]triazolo[1,5-*b*][1,2,4,5]tetrazine (5c).

To 1 mmol (371 mg) of 3-(4-bromo-3,5-dimethyl-1*H*-pyrazol-1-yl)-7-phenyl-[1,2,4]triazolo[1,5-*b*][1,2,4,5]tetrazine (**3j**) in 5 mL of acetonitrile 10 mmol (870 mg) of morpholine were added, reaction mixture was stirred for 5 hours at 80 °C and one day at room temperature. The precipitate was filtered and recrystallized from acetonitrile. Red solid, 83%, m.p. 243-244 °C; ^{1}H NMR ($CDCl_3$, 500MHz): δ/ppm 3.90, 4.01 (both t, *J*=4.6 Hz, 8H, $4CH_2$), 7.51-7.57 (m, 3H, Ph), 8.34-8.41 (m, 2H, Ph). Anal. Calcd for $C_{13}H_{13}N_7O$: C, 55.12; H, 4.63; N, 34.61; Found C, 55.10; H, 4.76; N, 34.49. LC/MS (ESI): Found *m/z* = 284.1257 (M+H). $C_{13}H_{14}N_7O^+$. Calculated 284.1254 (M+H).

6-Methyl-3-morpholino-[1,2,4]triazolo[4,3-*b*][1,2,4,5]tetrazine (11c).

To 1 mmol (230 mg) of 3-(3,5-dimethyl-1H-pyrazol-1-yl)-6-methyl-[1,2,4]triazolo[4,3-*b*][1,2,4,5]tetrazine (**10a**) in 5 mL of acetonitrile 2 mmol (174

mg) of morpholine were added, and reaction mixture was stirred for one day. The solvent was evaporated. The residue was washed with isopropanol and was filtered off. Orange solid, 72%, m.p. 165-166 °C; ^{1}H NMR (DMSO-d6, 400MHz): *δ*/ppm 2.61 (s, 3H, CH_3), 3.77-3.81, 3.82-3.90 (both m, 8H, 4CH_2). Anal. Calcd for $C_8H_{11}N_7O$: C, 43.43; H, 5.01; N, 44.32; Found C, 43.40; H, 4.84; N, 44.20. LC/MS (ESI): Found *m/z* = 222.1101 (M+H). $C_8H_{12}N_7O^+$. Calculated 222.1098 (M+H).

General procedure for the synthesis of *N'*-(6,7-dicyano-5-imino-3-R-[1,2,4]triazolo[4,3-*a*]pyrimidin-1(5*H*)-yl)-Het-1-carboximidamides 6a,b.

To 1 mmol of 3-(3,5-dimethyl-1*H*-pyrazol-1-yl)-7-methyl-[1,2,4]triazolo[1,5-*b*][1,2,4,5]tetrazine (**3a**) or 3-(4-bromo-3,5-dimethyl-1*H*-pyrazol-1-yl)-7-phenyl-[1,2,4]triazolo[1,5-*b*][1,2,4,5]tetrazine (**3j**) in 5 mL of acetonitrile 2 mmol (132 mg) of malononitrile and 2 mmol (202 mg) of triethylamine were added. Reaction was stirred at room temperature.

***N'*-(6,7-Dicyano-5-imino-3-methyl-[1,2,4]triazolo[4,3-*a*]pyrimidin-1(5*H*)-yl)-3,5-dimethyl-1*H*-pyrazole-1-carboximidamide (6a).**

Reaction time 1 hour, then the precipitate was filtered. White solid, 58%, m.p. > 350 °C; ^{1}H NMR (DMSO-d6, 400MHz): *δ*/ppm 2.33, 2.44, 2.56 (all s, 9H, 3CH_3), 6.25 (s, 1H, CH), 7.98 (s, 1H, NH), 8.00 (br s, 2H, NH_2); ^{13}C NMR (DMSO-d6, 100MHz): *δ*/ppm 10.1, 13.3, 14.8, 93.3, 110.9, 114.4, 114.8, 138.3, 142.9, 145.3, 146.7, 150.5, 151.1, 157.3. Anal. Calcd for $C_{14}H_{13}N_{11}$: C, 50.15; H, 3.91; N, 45.95; Found C, 50.16; H, 3.77; N, 46.16.

4-Bromo-*N'*-(6,7-dicyano-5-imino-3-phenyl-[1,2,4]triazolo[4,3-*a*]pyrimidin-1(5*H*)-yl)-3,5-dimethyl-1*H*-pyrazole-1-carboximidamide (6b).

Reaction time 2 days, then the solvent was evaporated from the reaction mixture. The substance was isolated by flash-chromatography (eluent benzene - acetonitrile, 1:1), the solvent was evaporated and the residue was washed with acetonitrile and was filtered off. Yellow solid, 20%, decomp. > 260 °C; ^{1}H NMR (DMSO-d6,

400MHz): *δ*/ppm 2.24, 2.58 (both s, 6H, 2CH_3), 6.30 (s, 2H, NH_2), 7.35-7.39 (m, 1H, Ph), 7.41-7.45, 7.94-7.97 (both m, 4H, Ph), 7.84 (br s, 1H, NH); ^{13}C NMR (DMSO-d6, 100MHz): *δ*/ppm 10.1, 13.3, 14.8, 93.3, 110.9, 114.4, 114.8, 138.3, 142.9, 145.3, 146.7, 150.5, 151.1, 157.3. Anal. Calcd for $C_{19}H_{14}BrN_{11}$: C, 47.91; H, 2.96; N, 32.35; Found C, 47.75; H, 2.80; N, 32.25. LC/MS (ESI): Found *m/z* = 476.0689 (M+H). $C_{19}H_{15}BrN_{11}^{+}$. Calculated 476.0690 (M+H).

Synthesis of *N'*-(5-amino-3-phenyl-1*H*-1,2,4-triazol-1-yl)-4-bromo-3,5-dimethyl-1*H*-pyrazole-1-carboximidamide (7), 6-(4-bromo-3,5-dimethyl-1*H*-pyrazol-1-yl)-8-ethoxycarbonyl-2-phenyl-7*H*-[1,2,4]triazolo[1,5-*b*][1,2,4,6]tetrazepine (8), 1-(amino(4-bromo-3,5-dimethyl-1*H*-pyrazol-1-yl)methyleneamino)-6,7-diethoxycarbonyl-5-imino-3-phenyl-1,5-dihydro-[1,2,4]triazolo[4,3-*a*]pyrimidine (9).

To 1 mmol (371 mg) of 3-(4-bromo-3,5-dimethyl-1*H*-pyrazol-1-yl)-7-phenyl-[1,2,4]triazolo[1,5-*b*][1,2,4,5]tetrazine (**3j**) in 5 mL of acetonitrile 5 mmol (565 mg) of ethyl cyanoacetate, and 5 mmol (505 mg) of triethylamine were added. Reaction mixture was stirred at room temperature for 1 day. The precipitate **7** was filtered off, the solvent was evaporated from the filtrate, the residue was washed with acetonitrile and filtered to give a mixture of **8** and **9**.

***N'*-(5-Amino-3-phenyl-1*H*-1,2,4-triazol-1-yl)-4-bromo-3,5-dimethyl-1*H*-pyrazole-1-carboximidamide (7).** White solid, 20%, m.p. 230-231 °C; ^{1}H NMR (DMSO-d6, 500MHz): *δ*/ppm 2.24, 2.58 (both s, 6H, 2CH_3), 6.30 (s, 2H, NH_2), 7.35-7.39 (m, 1H, Ph), 7.41-7.46, 7.93-7.97 (both m, 4H, Ph), 7.86 (br s, 2H, NH_2); ^{13}C NMR (DMSO-d6, 125MHz): *δ*/ppm 12.3, 13.4, 98.1, 125.4, 128.5, 128.6, 131.3, 139.5, 146.4, 147.4, 153.0, 153.8. Anal. Calcd for $C_{14}H_{15}BrN_8$: C, 44.81; H, 4.03; N, 29.86; Found C, 44.71; H, 4.20; N, 29.56.

6-(4-Bromo-3,5-dimethyl-1*H*-pyrazol-1-yl)-8-ethoxycarbonyl-2-phenyl-7*H*-[1,2,4]triazolo[1,5-*b*][1,2,4,6]tetrazepine (8). ^{1}H NMR (DMSO-d6, 500MHz): *δ*/ppm 1.17 (t, *J*= 7.1 Hz, 3H, $COOCH_2\underline{CH_3}$), 2.25, 2.61 (both s, 6H, 2CH_3), 4.25 (q,

J = 7.1 Hz, 4H, COO$\underline{CH_2}CH_3$), 7.37-7.45 (m, 3H, Ph), 7.92 (br s, 1H, NH) 7.93-7.97 (m, 2H, Ph). LC/MS (APCI): Found *m/z* = 455.0583 (M-H). $C_{18}H_{16}BrN_8O_2^-$. Calculated 455.0585 (M-H).

1-(Amino(4-bromo-3,5-dimethyl-1*H*-pyrazol-1-yl)methyleneamino)-6,7-diethoxycarbonyl-5-imino-3-phenyl-1,5-dihydro-[1,2,4]triazolo[4,3-*a*]pyrimidine (9). ^{1}H NMR (DMSO-d6, 500MHz): δ/ppm 1.28, 1.32 (both t, *J* = 7.1 Hz, 6H, 2COOCH$_2\underline{CH_3}$), 2.26, 2.64 (both s, 6H, 2CH_3), 4.31, 4.52 (both q, *J* = 7.1 Hz, 4H, 2COO$\underline{CH_2}CH_3$), 7.45-7.53 (m, 3H, Ph), 7.97-8.01 (m, 2H, Ph), 8.26 (br s, 2H, NH_2), 11.61 (s, 1H, NH). LC/MS (APCI): Found *m/z* = 568.1067 (M-H). $C_{23}H_{23}BrN_9O_4^-$. Calculated 568.1062 (M-H).

References

1. Coburn, M. D.; Buntain, G. A.; Harris, B. W.; Hiskey, M. A.; Lee, K.-Y.; Ott, D. G. *J. Heterocycl. Chem.*, **1991,** *28,* 2049-2050. DOI: 10.1002/jhet.5570280844
2. Rusinov, G. L.; Ganebnykh, I. N.; Chupakhin, O. N. *Russ. J. Org. Chem. (Translation of Zhurnal Organicheskoi Khimii),* **1999,** *35,* 1350-1354.
3. Sheldrick, G. M. *Acta Cryst.,* **2008,** *A64,* 112-122. DOI: 10.1107/S0108767307043930
4. Shcherbakov, K. V.; Artemyeva M. A.; Burgart, Y. V.; Evstigneeva, N. P.; Gerasimova, N. A.; Zilberberg, N. V.; Kungurov, N. V.; Saloutin, V. I.; Chupakhin, O. N. *J. Fluorine Chem.,* **2019,** *226,* No. 109354. DOI: 10.1016/j.jfluchem.2019.109354
5. Methodological guidelines for the study of antifungal activity of medicines. In Guidelines for conducting preclinical studies of medicines. Part One; Mironov, A. N. Ed.; Grif and K, Moscow, 2012, pp 578-586.

NMR spectra of synthesized compounds.

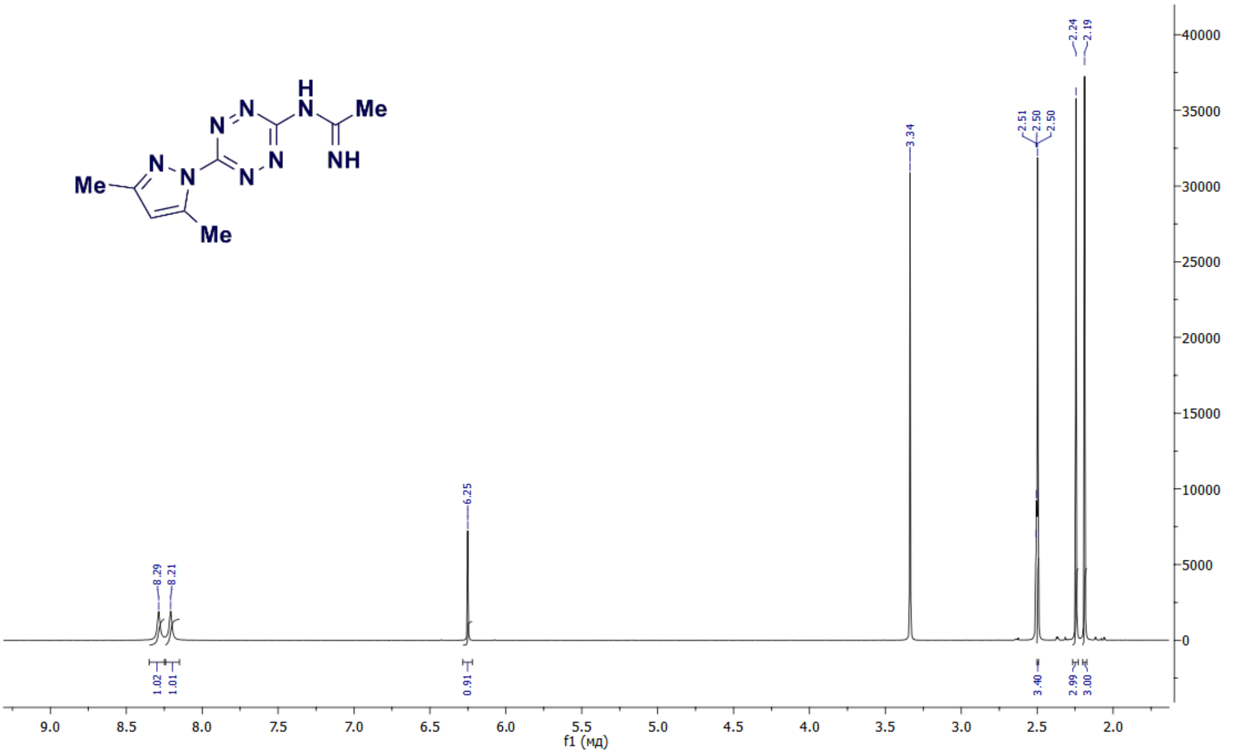

Figure S1. ^{1}H NMR spectrum of the *N*-(6-(3,5-dimethyl-1*H*-pyrazol-1-yl)-1,2,4,5-tetrazin-3-yl)acetimidamide (**2a**).

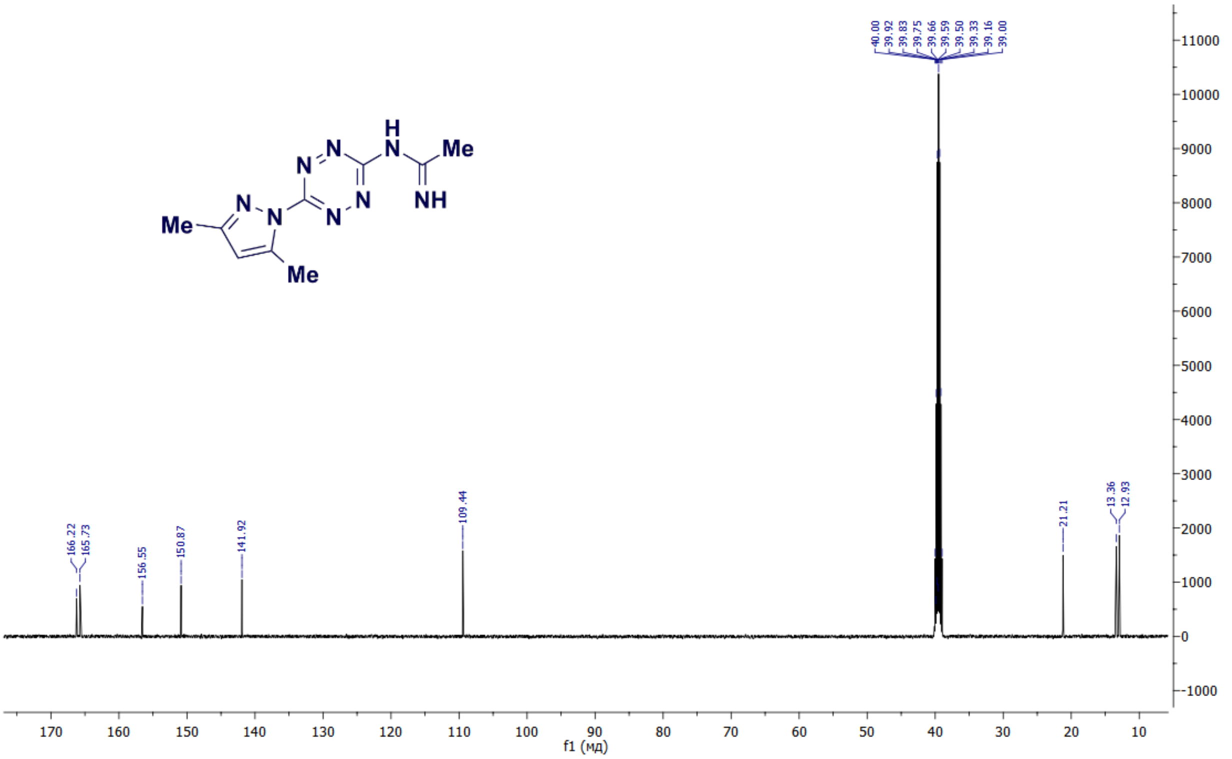

Figure S2. ^{13}C NMR spectrum of the *N*-(6-(3,5-dimethyl-1*H*-pyrazol-1-yl)-1,2,4,5-tetrazin-3-yl)acetimidamide (**2a**).

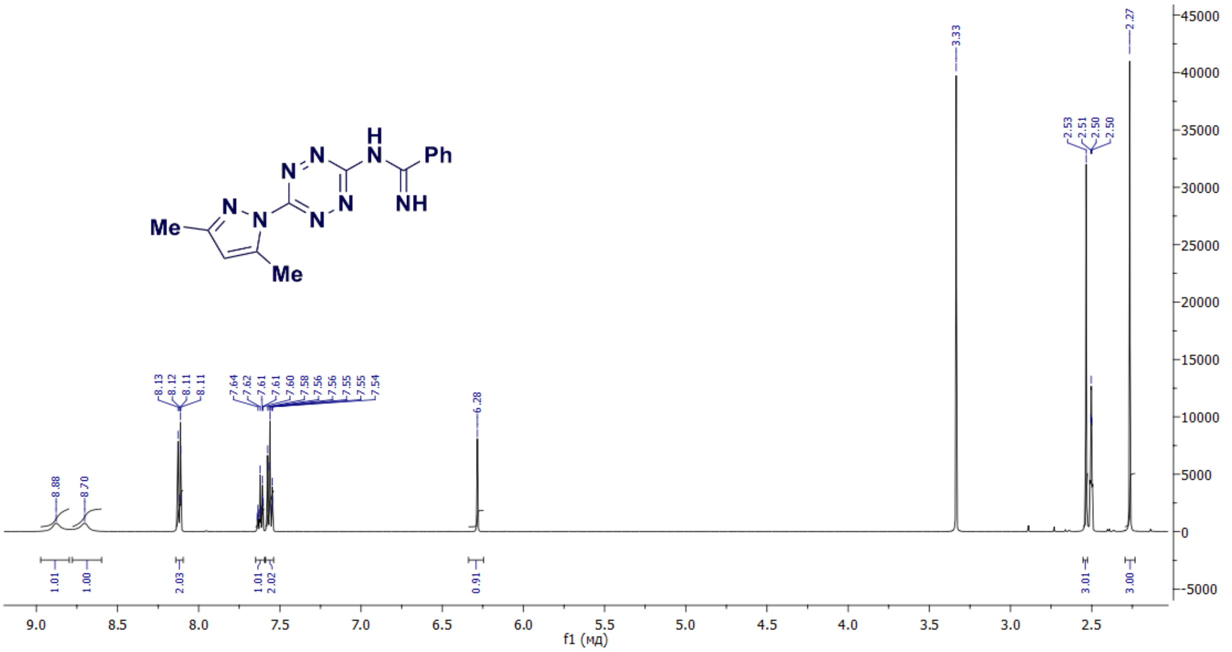

Figure S3. ^{1}H NMR spectrum of the *N*-(6-(3,5-dimethyl-1*H*-pyrazol-1-yl)-1,2,4,5-tetrazin-3-yl)benzimidamide (**2b**).

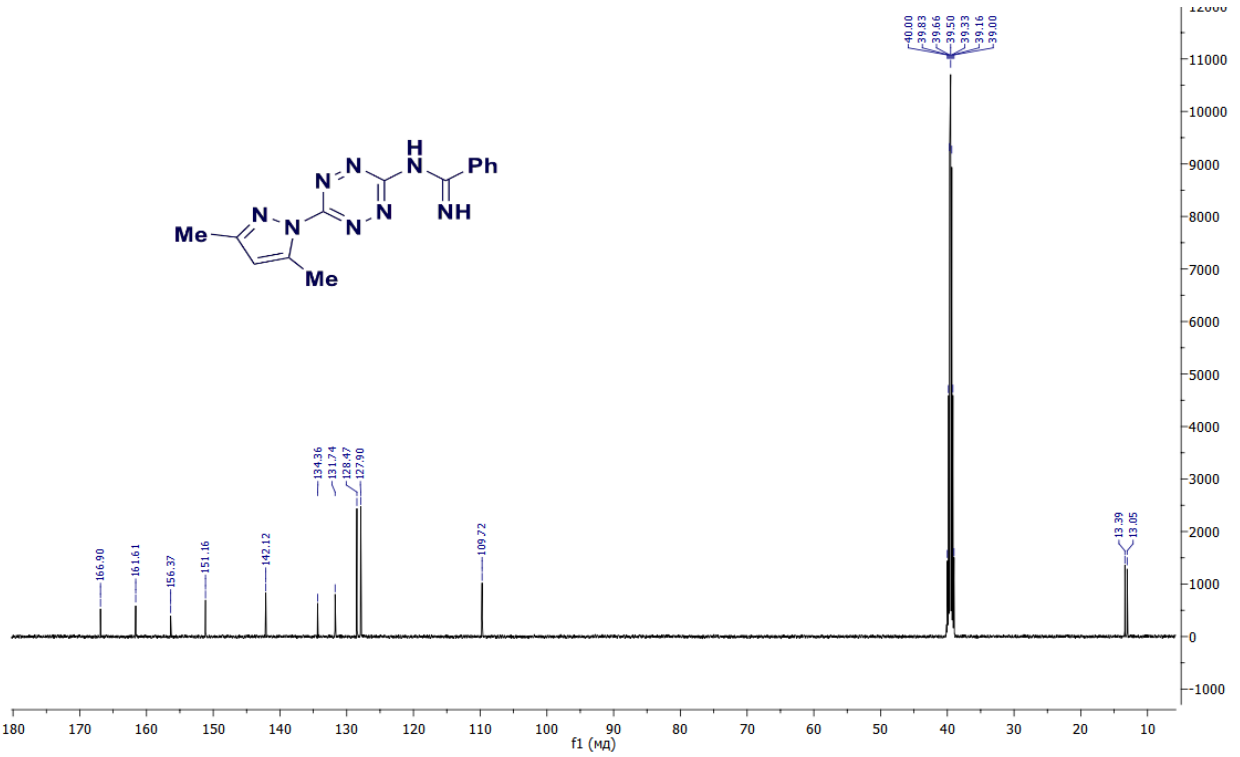

Figure S4. ^{13}C NMR spectrum of the *N*-(6-(3,5-dimethyl-1*H*-pyrazol-1-yl)-1,2,4,5-tetrazin-3-yl)benzimidamide (**2b**).

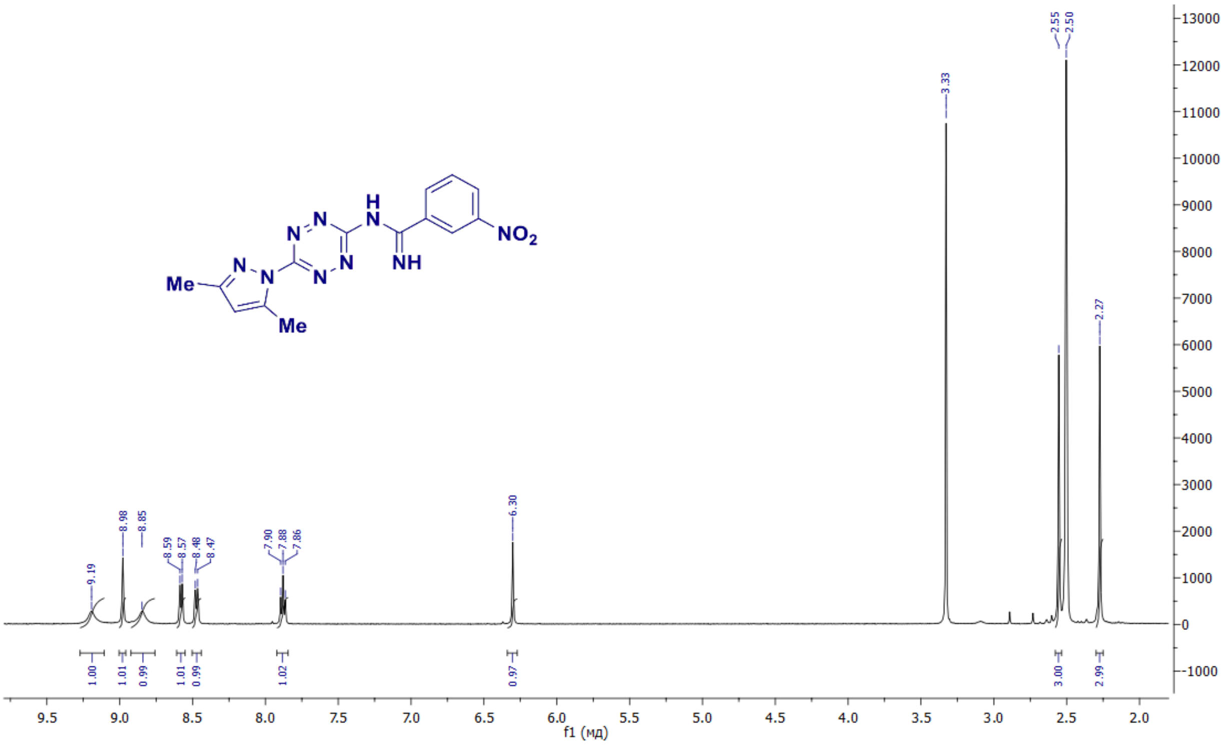

Figure S5. ^{1}H NMR spectrum of the *N*-(6-(3,5-dimethyl-1*H*-pyrazol-1-yl)-1,2,4,5-tetrazin-3-yl)-3-nitrobenzimidamide (**2c**).

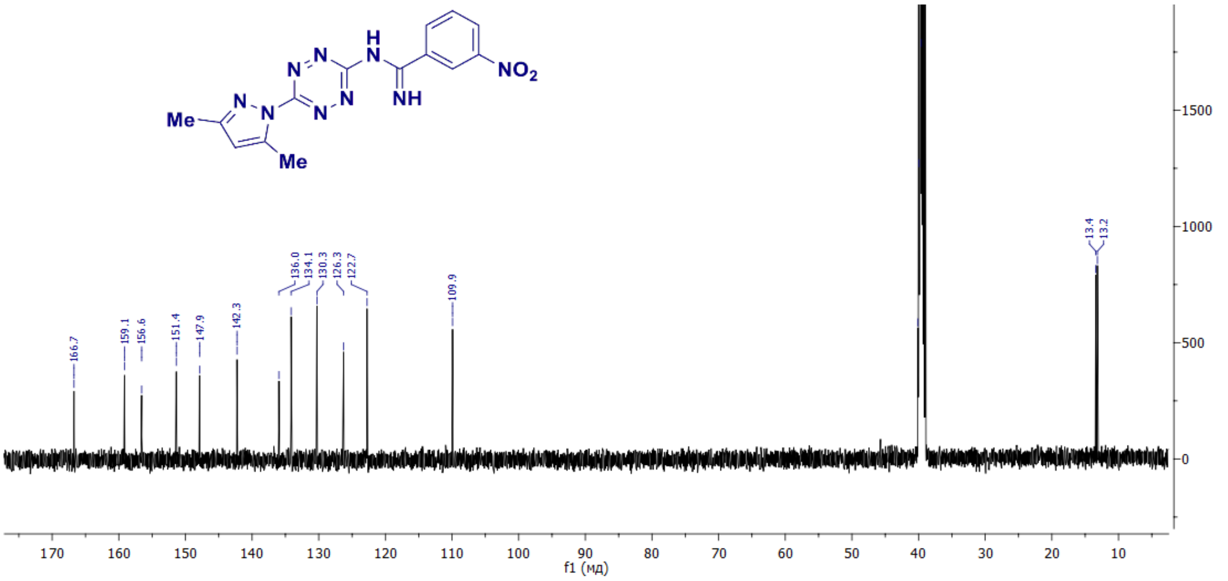

Figure S6. ^{13}C NMR spectrum of the *N*-(6-(3,5-dimethyl-1*H*-pyrazol-1-yl)-1,2,4,5-tetrazin-3-yl)-3-nitrobenzimidamide (**2c**).

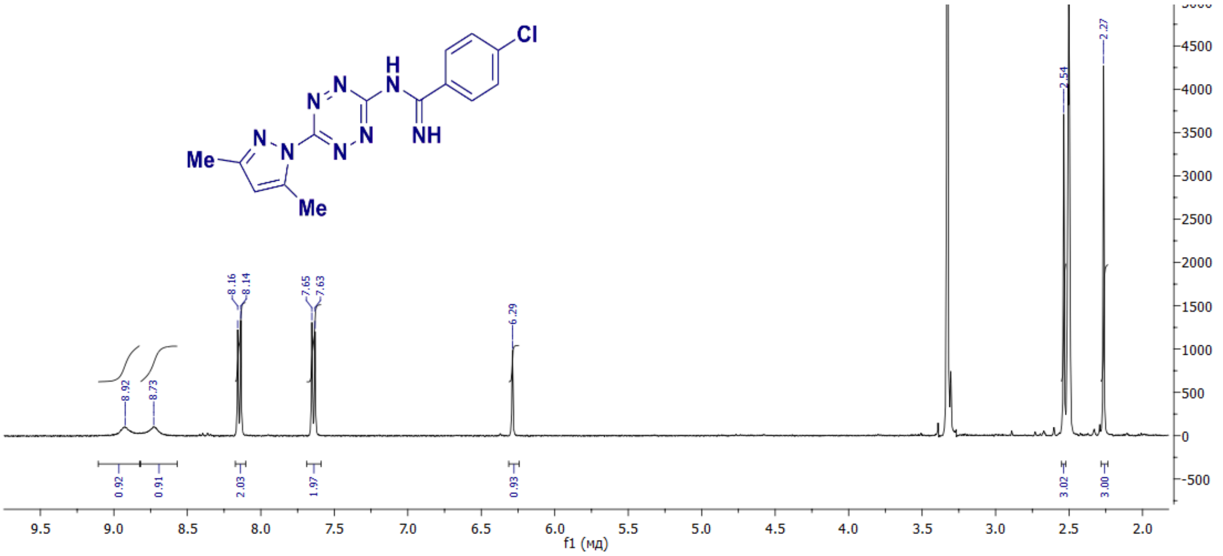

Figure S7. ^{1}H NMR spectrum of the *N*-(6-(3,5-dimethyl-1H-pyrazol-1-yl)-1,2,4,5-tetrazin-3-yl)-4-chlorobenzimidamide (**2d**).

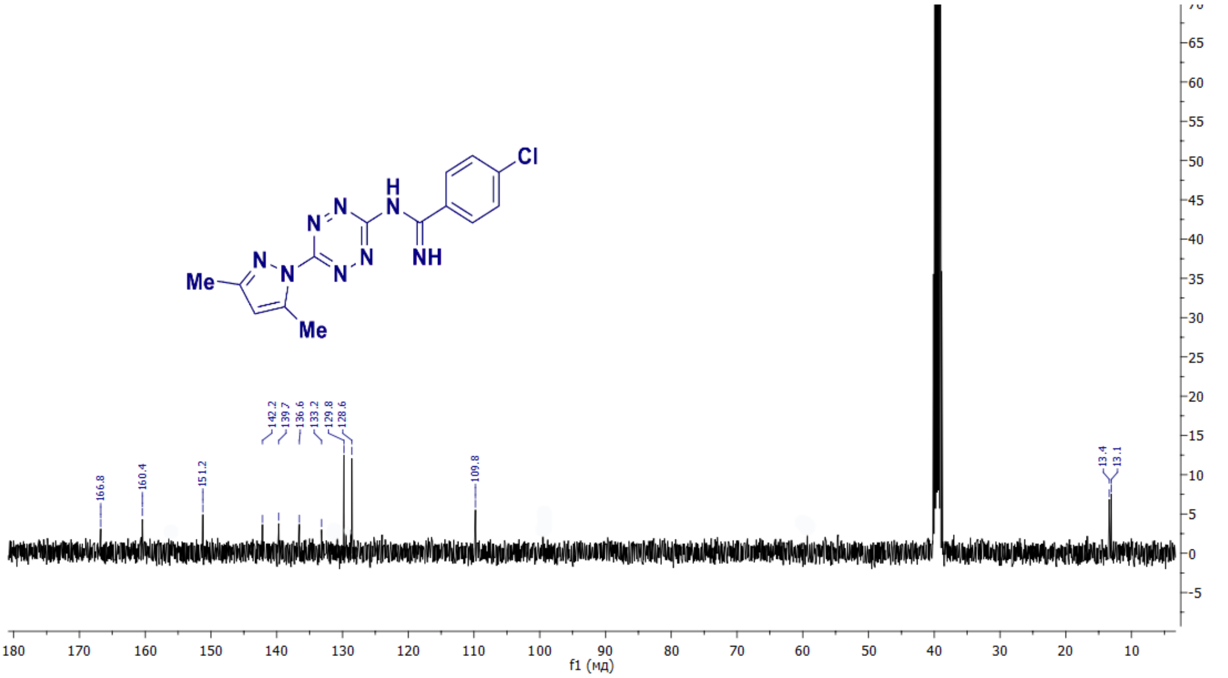

Figure S8. ^{13}C NMR spectrum of the *N*-(6-(3,5-dimethyl-1H-pyrazol-1-yl)-1,2,4,5-tetrazin-3-yl)-4-chlorobenzimidamide (**2d**).

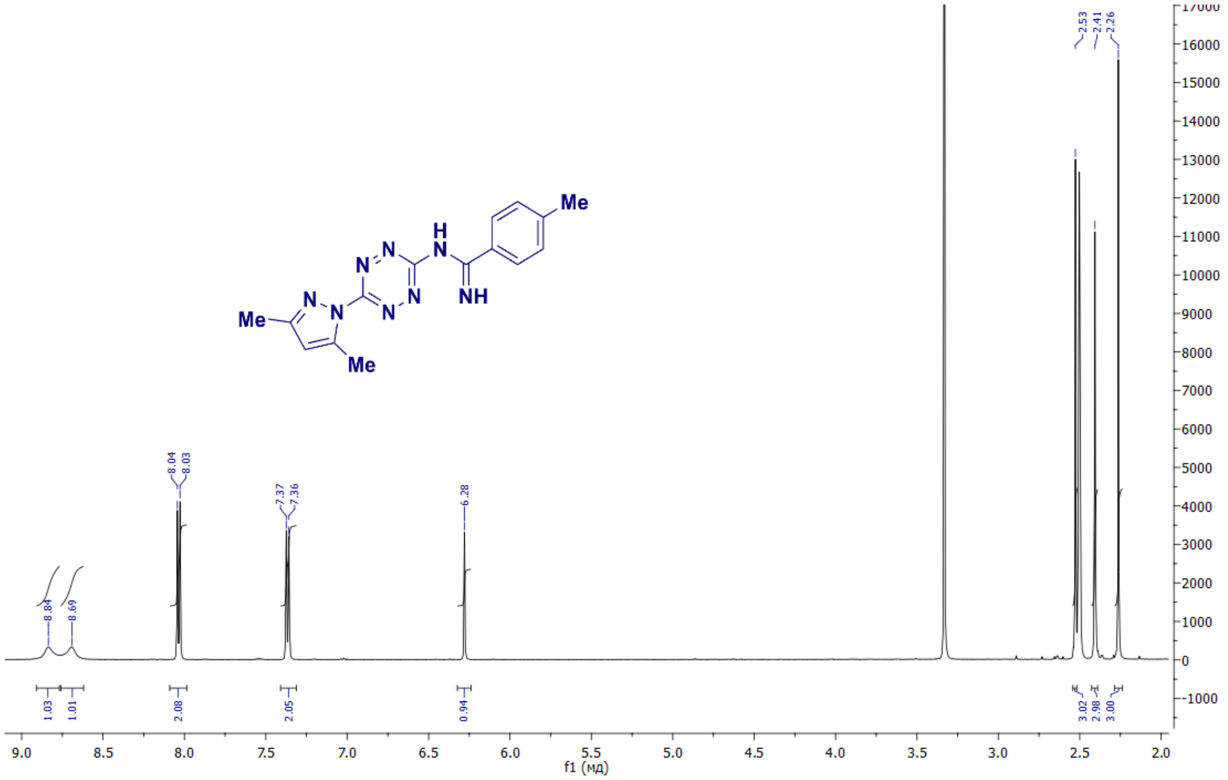

Figure S9. ^{1}H NMR spectrum of the *N*-(6-(3,5-dimethyl-1H-pyrazol-1-yl)-1,2,4,5-tetrazin-3-yl)-4-methylbenzimidamide (**2e**).

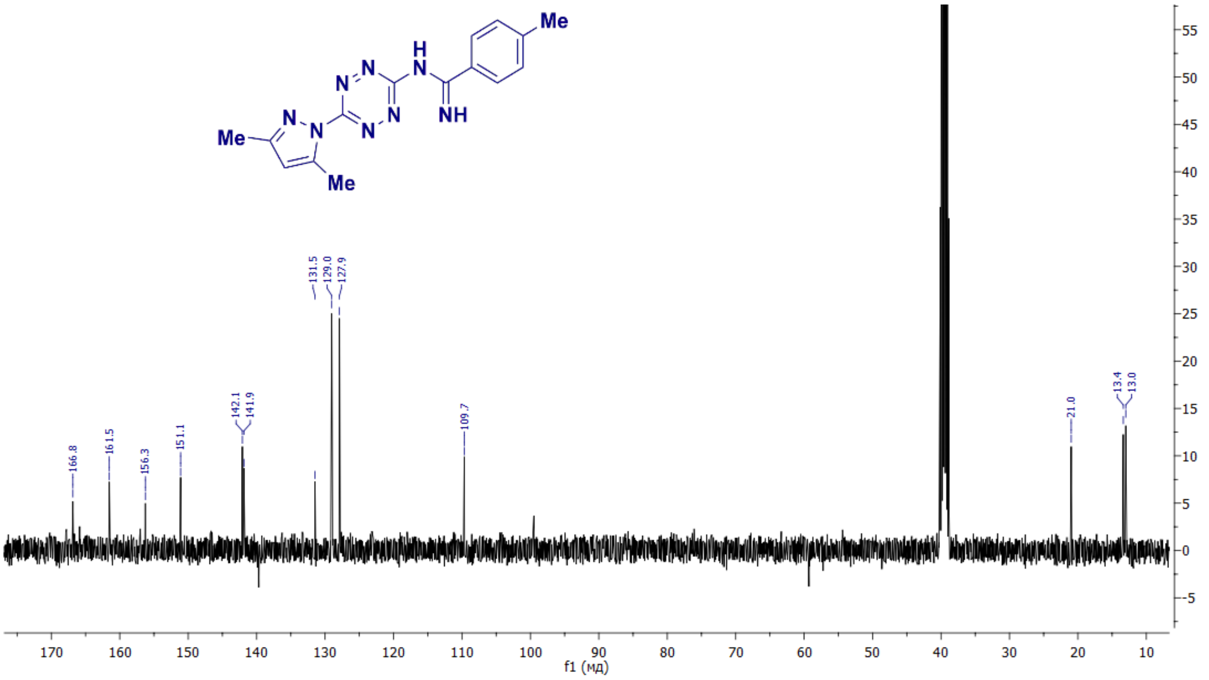

Figure S10. ^{13}C NMR spectrum of the *N*-(6-(3,5-dimethyl-1H-pyrazol-1-yl)-1,2,4,5-tetrazin-3-yl)-4-methylbenzimidamide (**2e**).

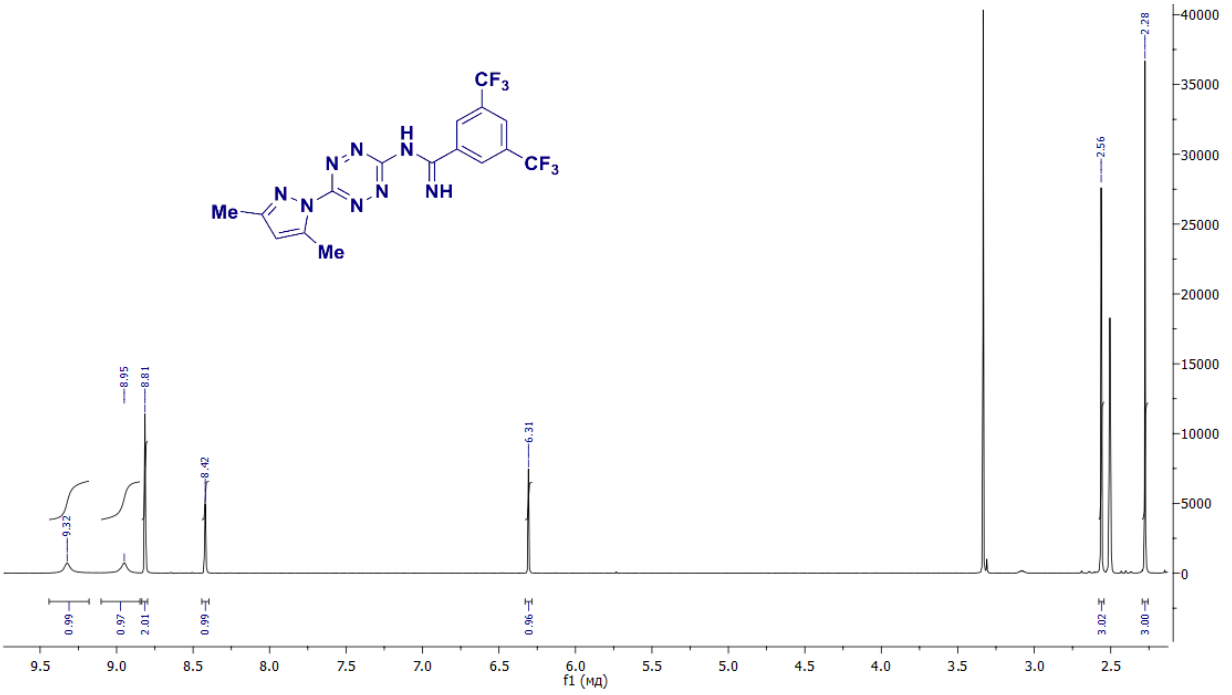

Figure S11. ^{1}H NMR spectrum of the *N*-(6-(3,5-dimethyl-1*H*-pyrazol-1-yl)-1,2,4,5-tetrazin-3-yl)-3,5-bis(trifluoromethyl)benzimidamide (**2f**).

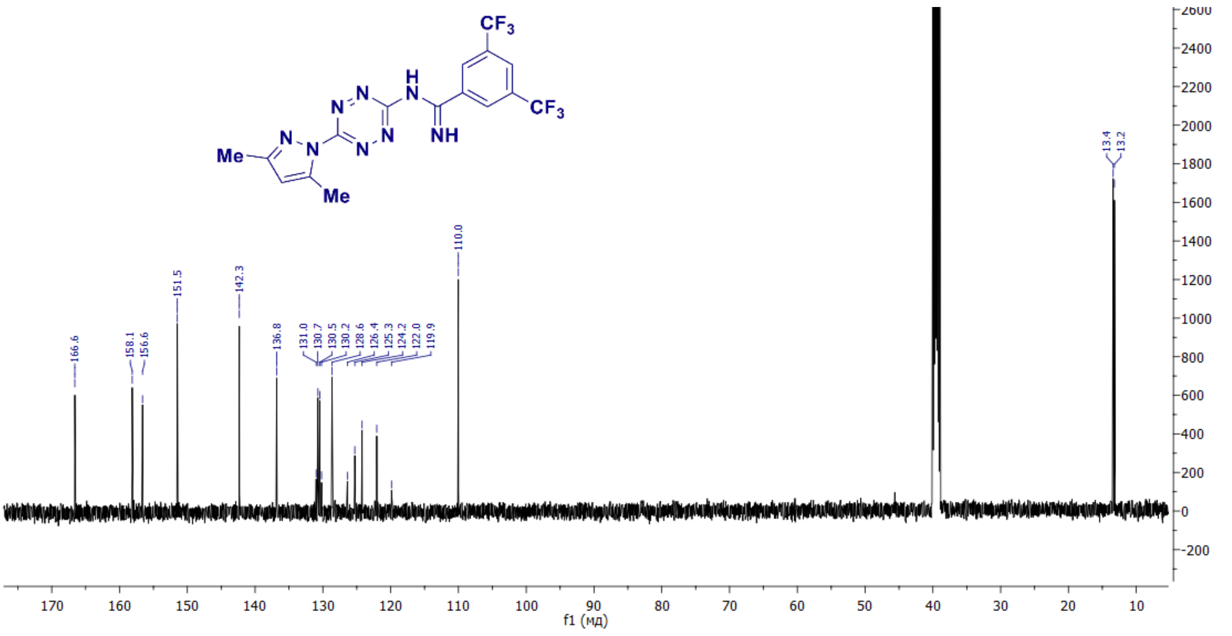

Figure S12. ^{13}C NMR spectrum of the *N*-(6-(3,5-dimethyl-1*H*-pyrazol-1-yl)-1,2,4,5-tetrazin-3-yl)-3,5-bis(trifluoromethyl)benzimidamide (**2f**).

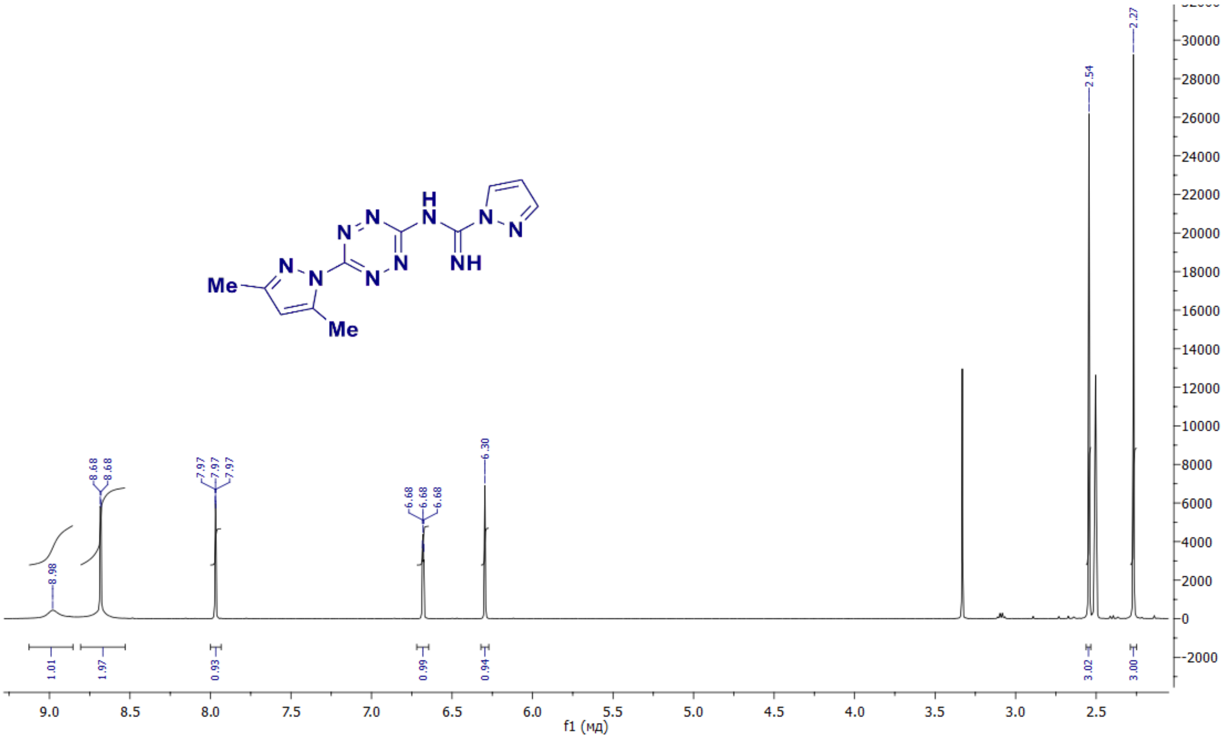

Figure S13. ^{1}H NMR spectrum of the *N*-(6-(3,5-dimethyl-1*H*-pyrazol-1-yl)-1,2,4,5-tetrazin-3-yl)-1*H*-pyrazole-1-carboximidamide (**2g**).

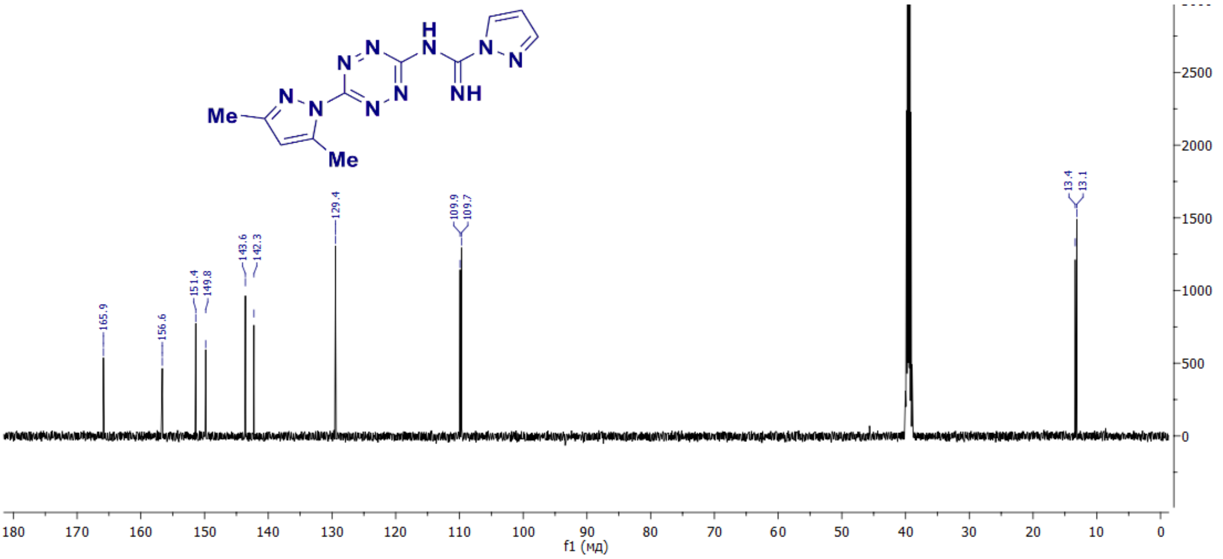

Figure S14. ^{13}C NMR spectrum of the *N*-(6-(3,5-dimethyl-1*H*-pyrazol-1-yl)-1,2,4,5-tetrazin-3-yl)-1*H*-pyrazole-1-carboximidamide (**2g**).

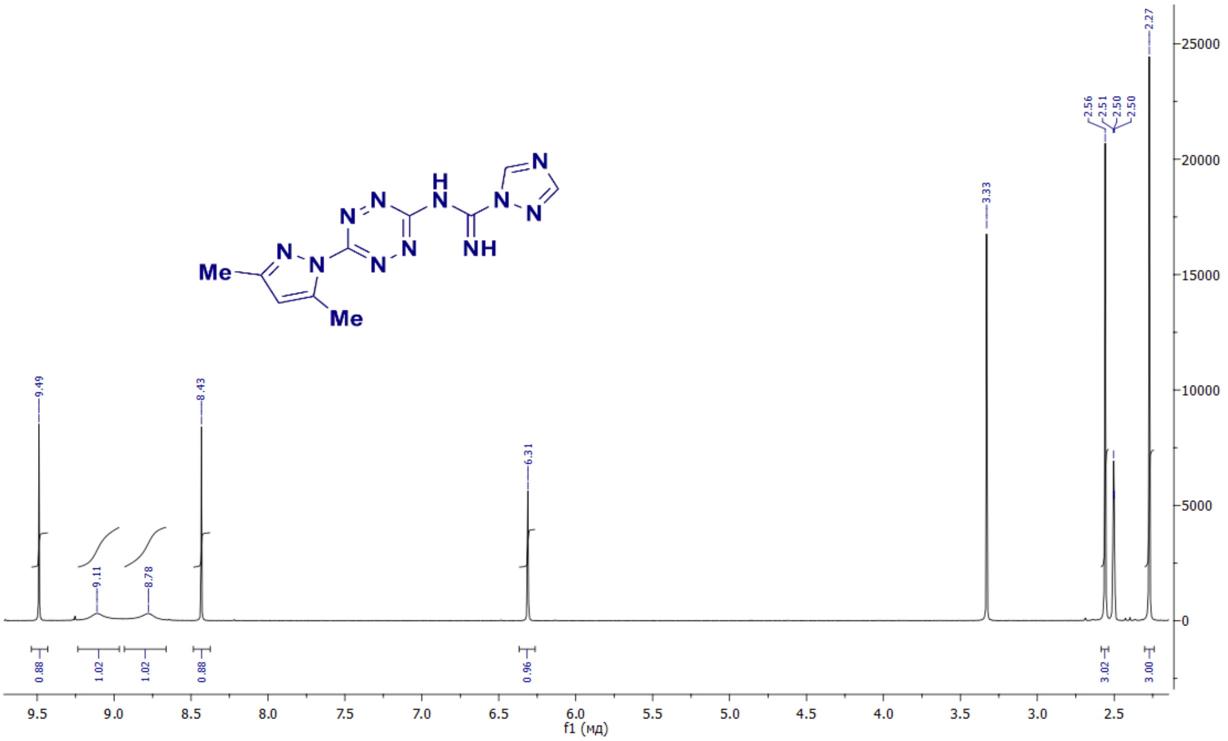

Figure S15. ^{1}H NMR spectrum of the *N*-(6-(3,5-dimethyl-1*H*-pyrazol-1-yl)-1,2,4,5-tetrazin-3-yl)-1*H*-1,2,4-triazole-1-carboximidamide (**2h**).

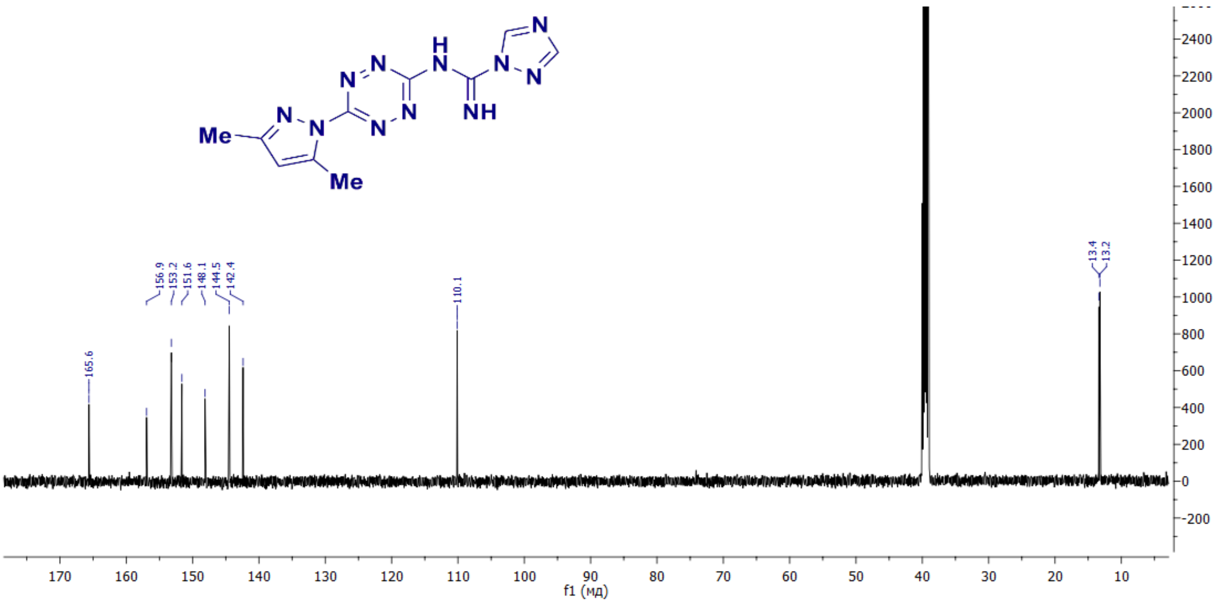

Figure S16. ^{13}C NMR spectrum of the *N*-(6-(3,5-dimethyl-1*H*-pyrazol-1-yl)-1,2,4,5-tetrazin-3-yl)-1*H*-1,2,4-triazole-1-carboximidamide (**2h**).

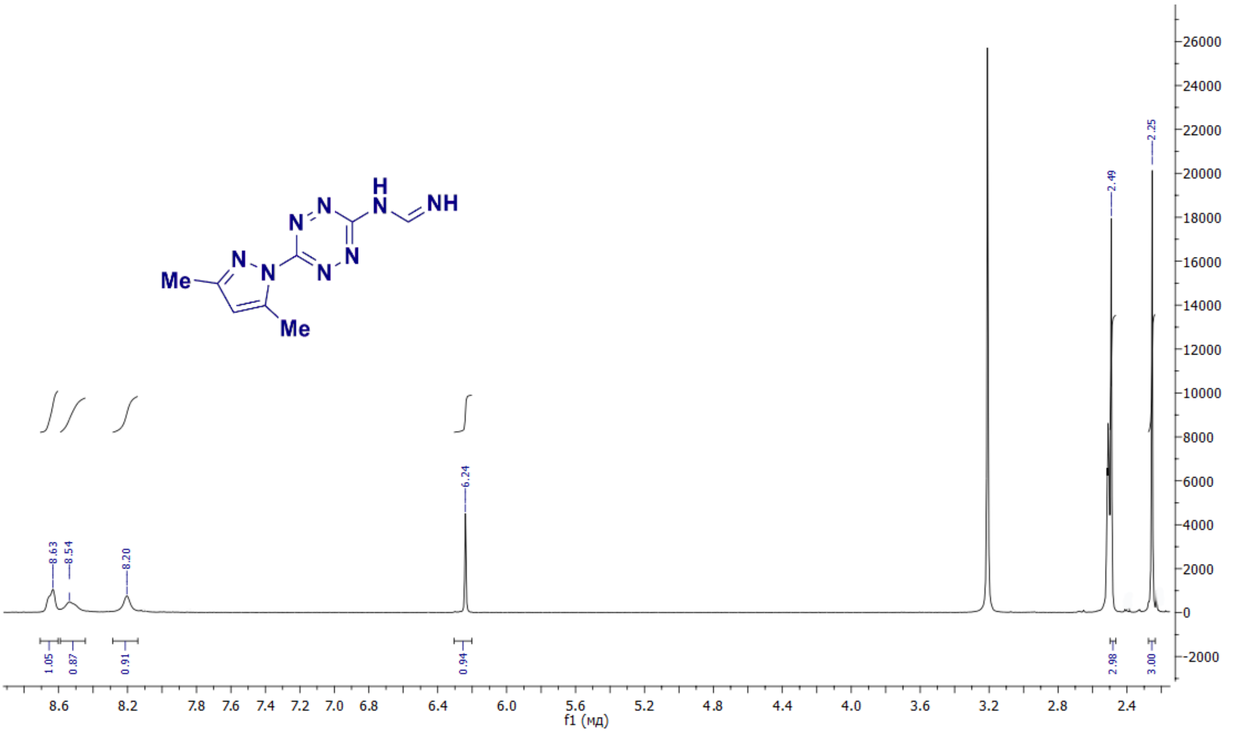

Figure S17. ^{1}H NMR spectrum of the *N*-(6-(3,5-dimethyl-1*H*-pyrazol-1-yl)-1,2,4,5-tetrazin-3-yl)formimidamide (**2i**).

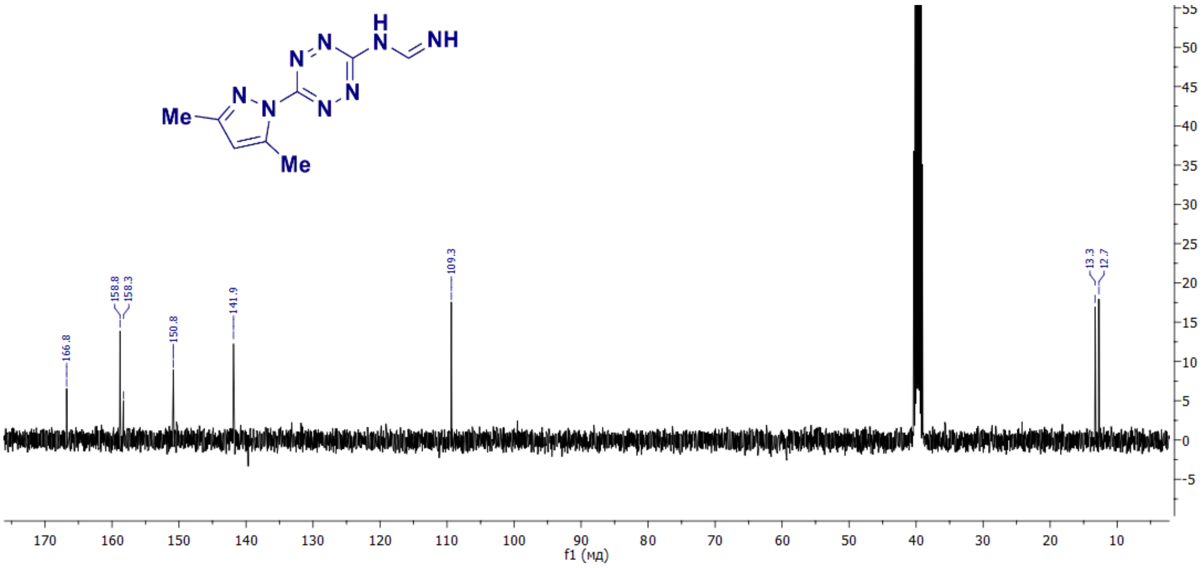

Figure S18. ^{13}C NMR spectrum of the *N*-(6-(3,5-dimethyl-1*H*-pyrazol-1-yl)-1,2,4,5-tetrazin-3-yl)formimidamide (**2i**).

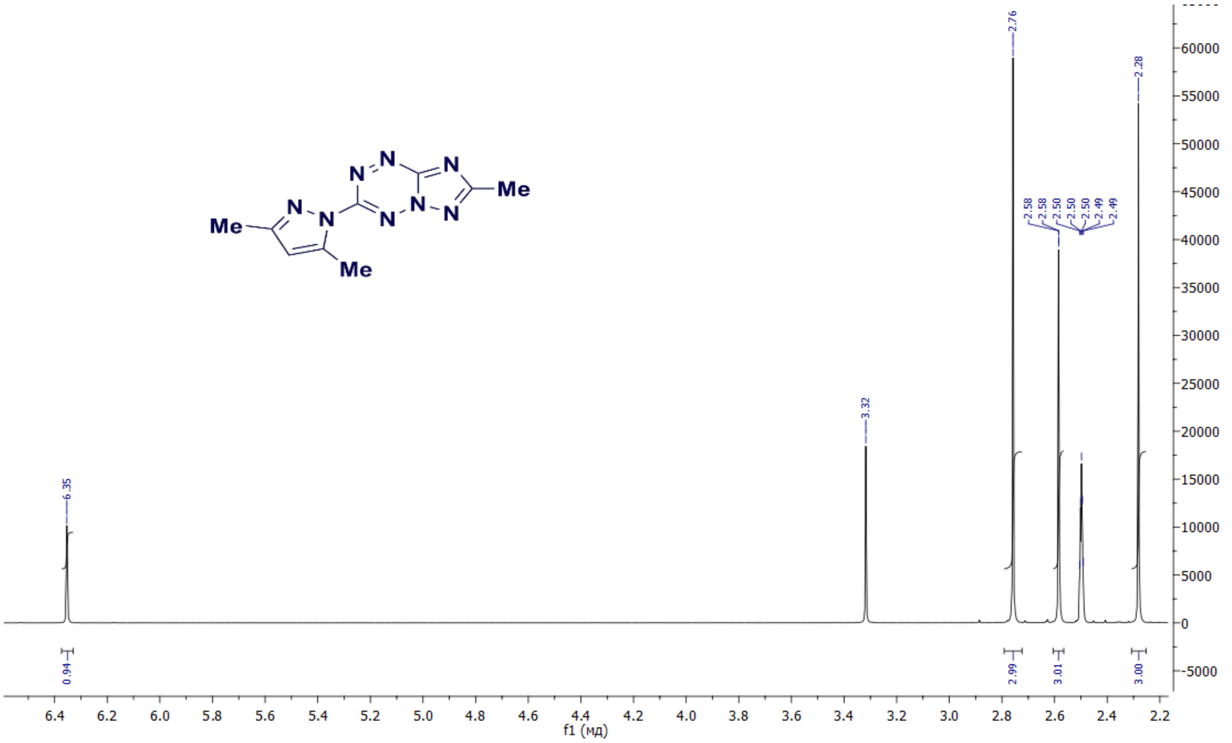

Figure S19. ^{1}H NMR spectrum of the 3-(3,5-dimethyl-1*H*-pyrazol-1-yl)-7-methyl-[1,2,4]triazolo[1,5-*b*][1,2,4,5]tetrazine (**3a**).

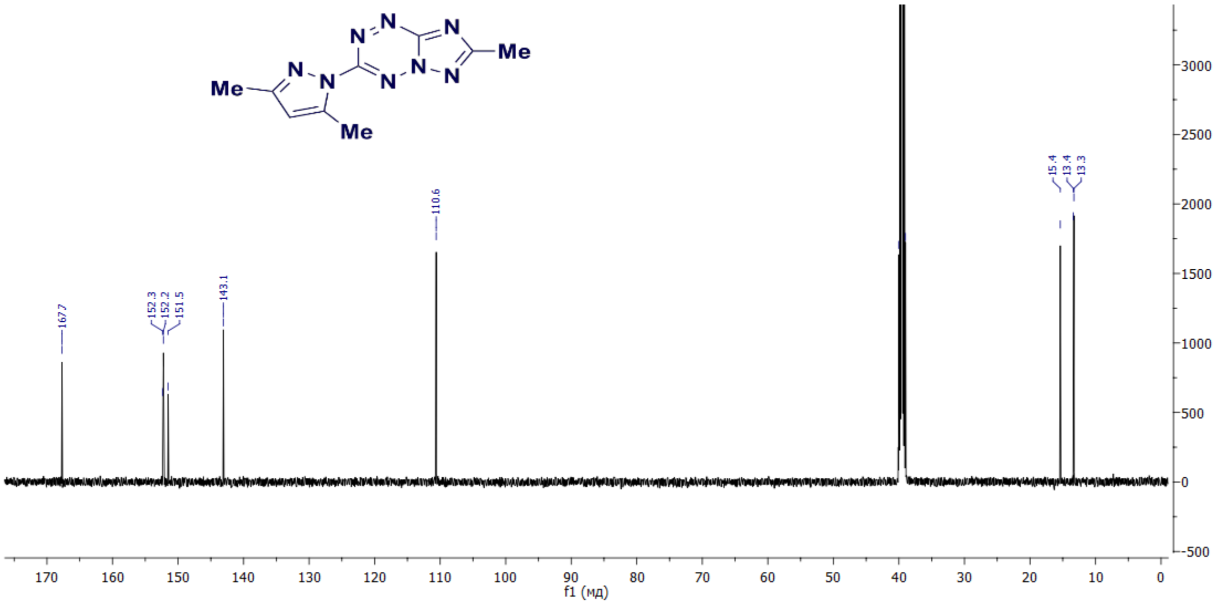

Figure S20. ^{13}C NMR spectrum of the 3-(3,5-dimethyl-1*H*-pyrazol-1-yl)-7-methyl-[1,2,4]triazolo[1,5-*b*][1,2,4,5]tetrazine (**3a**).

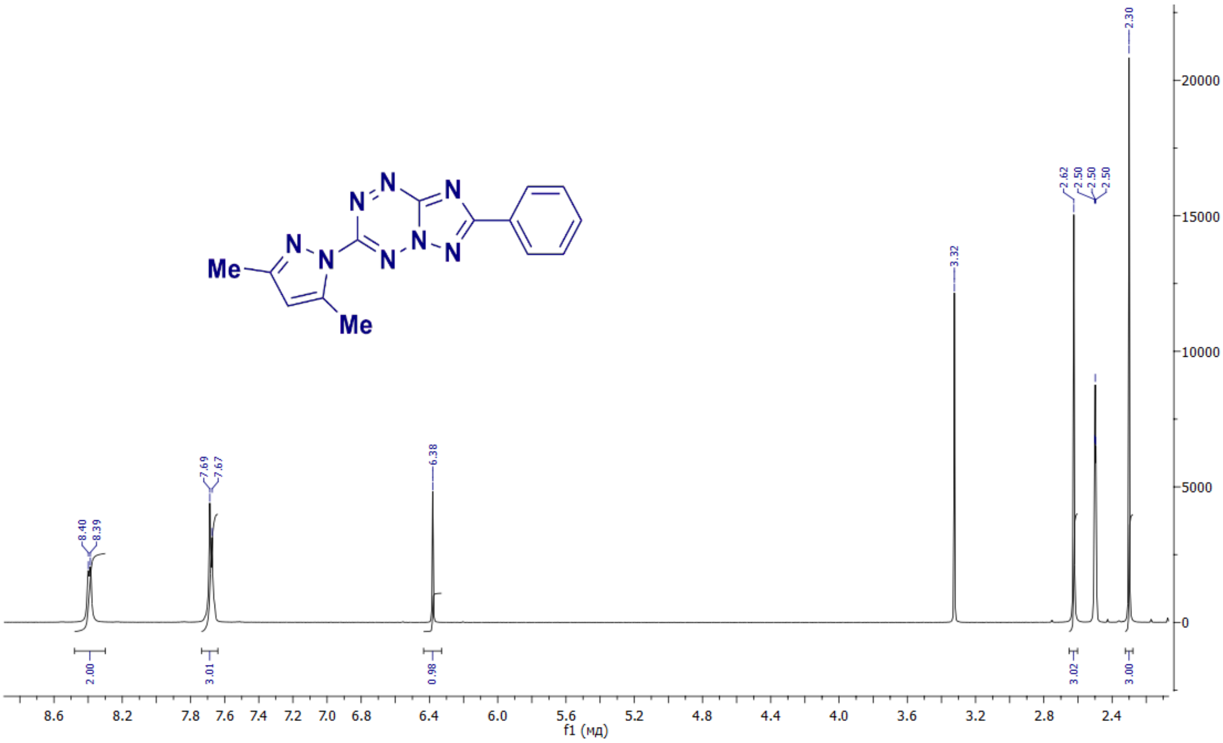

Figure S21. ^{1}H NMR spectrum of the 3-(3,5-dimethyl-1*H*-pyrazol-1-yl)-7-phenyl-[1,2,4]triazolo[1,5-*b*][1,2,4,5]tetrazine (**3b**).

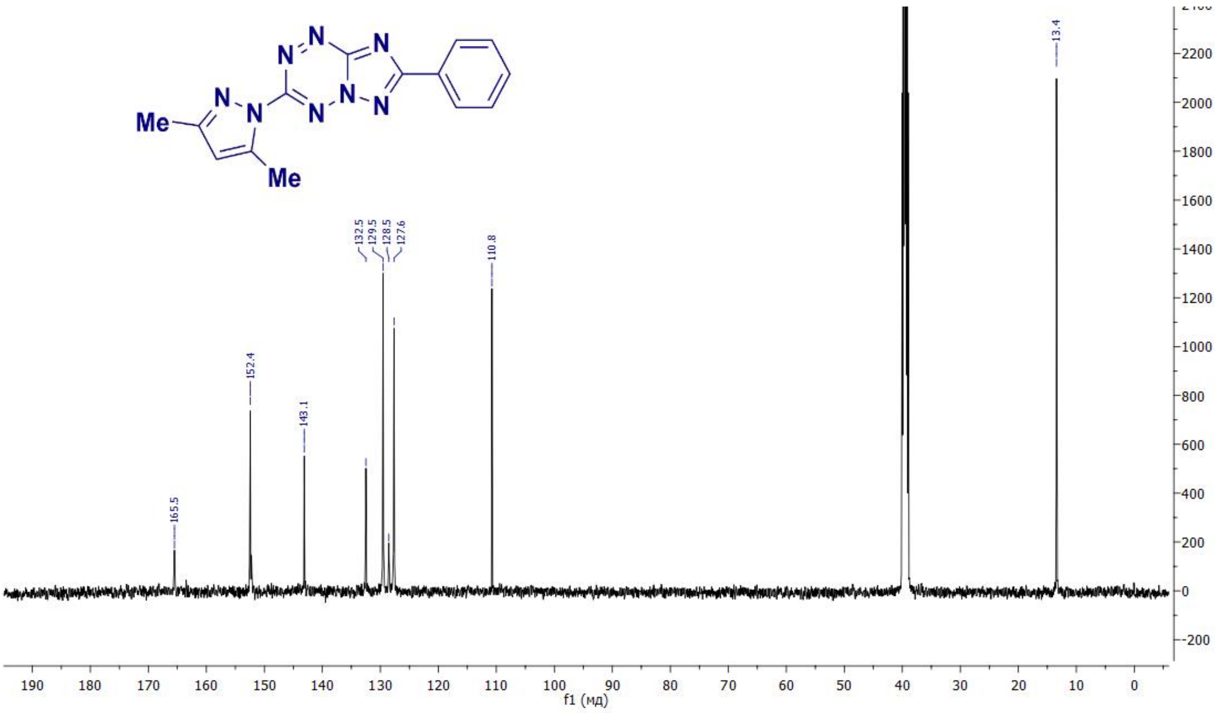

Figure S22. ^{13}C NMR spectrum of the 3-(3,5-dimethyl-1*H*-pyrazol-1-yl)-7-phenyl-[1,2,4]triazolo[1,5-*b*][1,2,4,5]tetrazine (**3b**).

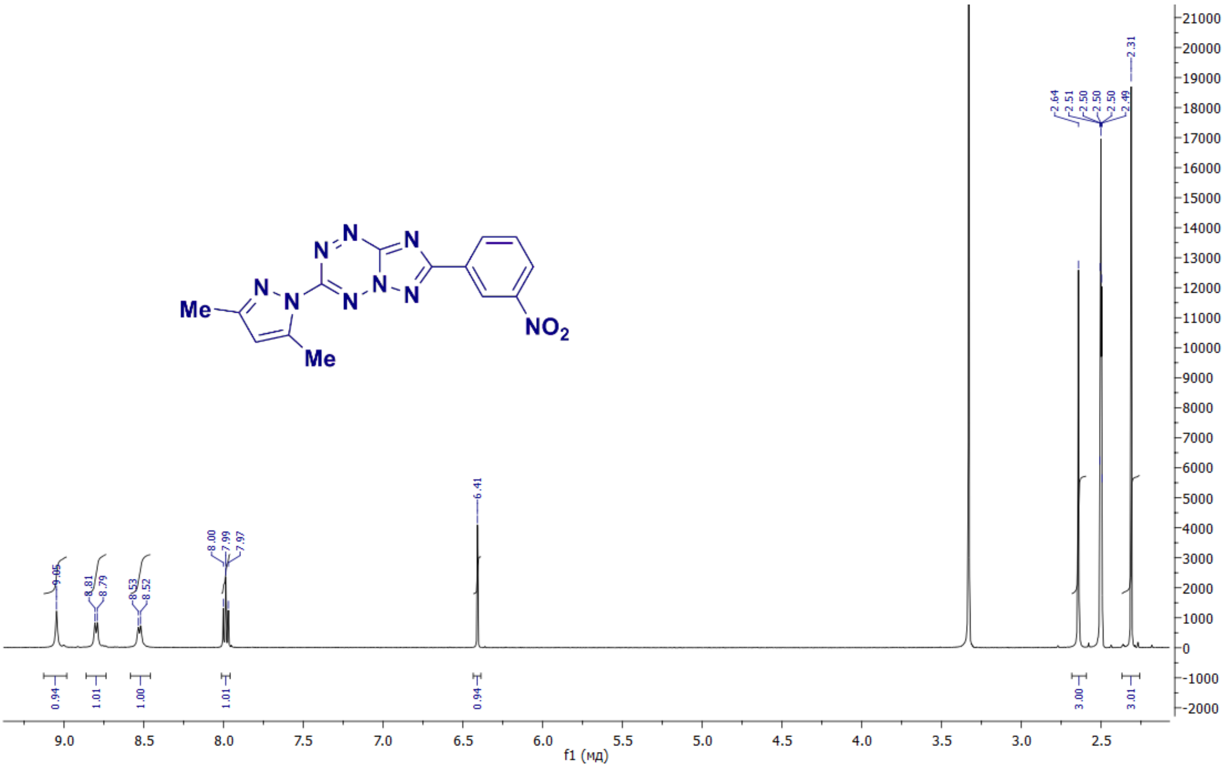

Figure S23. ^{1}H NMR spectrum of the 3-(3,5-dimethyl-1*H*-pyrazol-1-yl)-7-(3-nitrophenyl)[1,2,4]triazolo[1,5-*b*][1,2,4,5]tetrazine (**3c**).

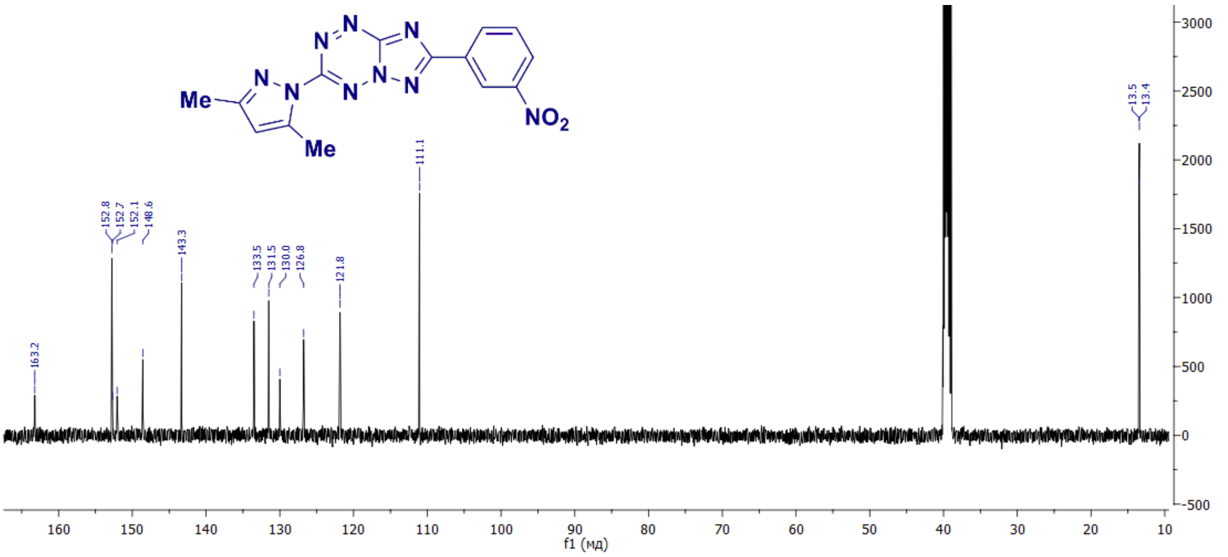

Figure S24. ^{13}C NMR spectrum of the 3-(3,5-dimethyl-1*H*-pyrazol-1-yl)-7-(3-nitrophenyl)-[1,2,4]triazolo[1,5-*b*][1,2,4,5]tetrazine (**3c**).

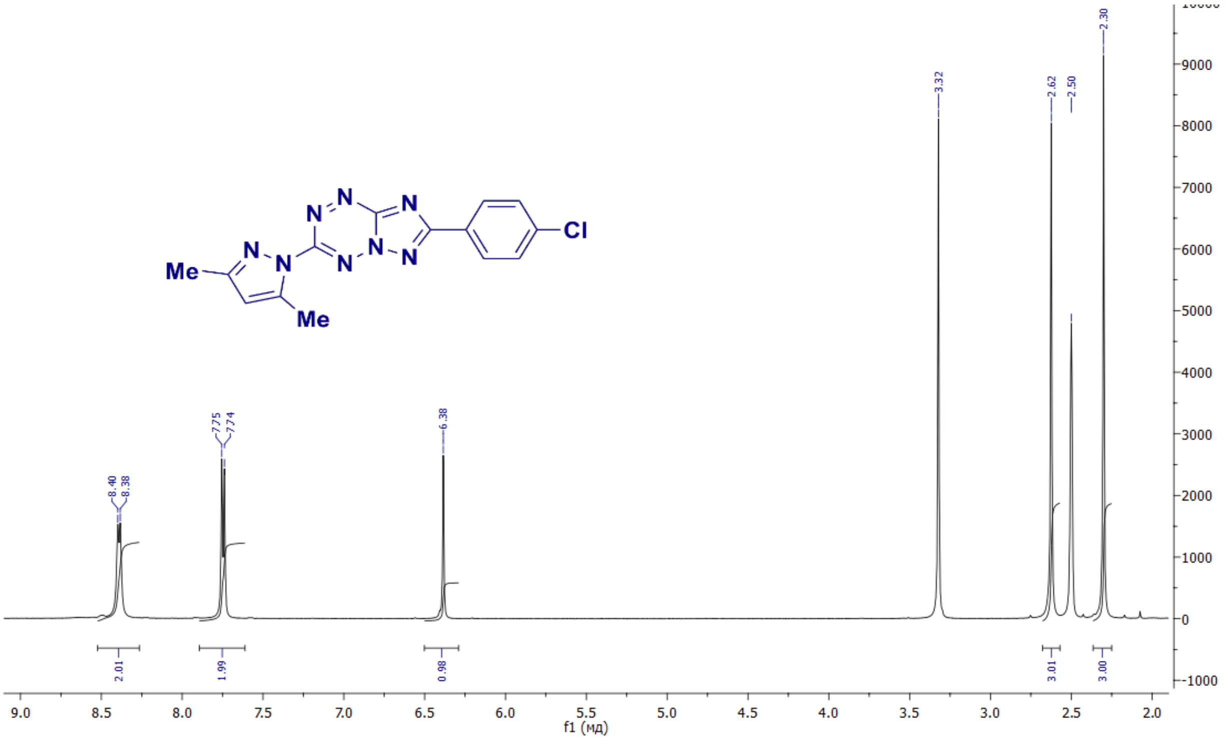

Figure S25. ^{1}H NMR spectrum of the 7-(4-chlorophenyl)-3-(3,5-dimethyl-1*H*-pyrazol-1-yl)-[1,2,4]triazolo[1,5-*b*][1,2,4,5]tetrazine (**3d**).

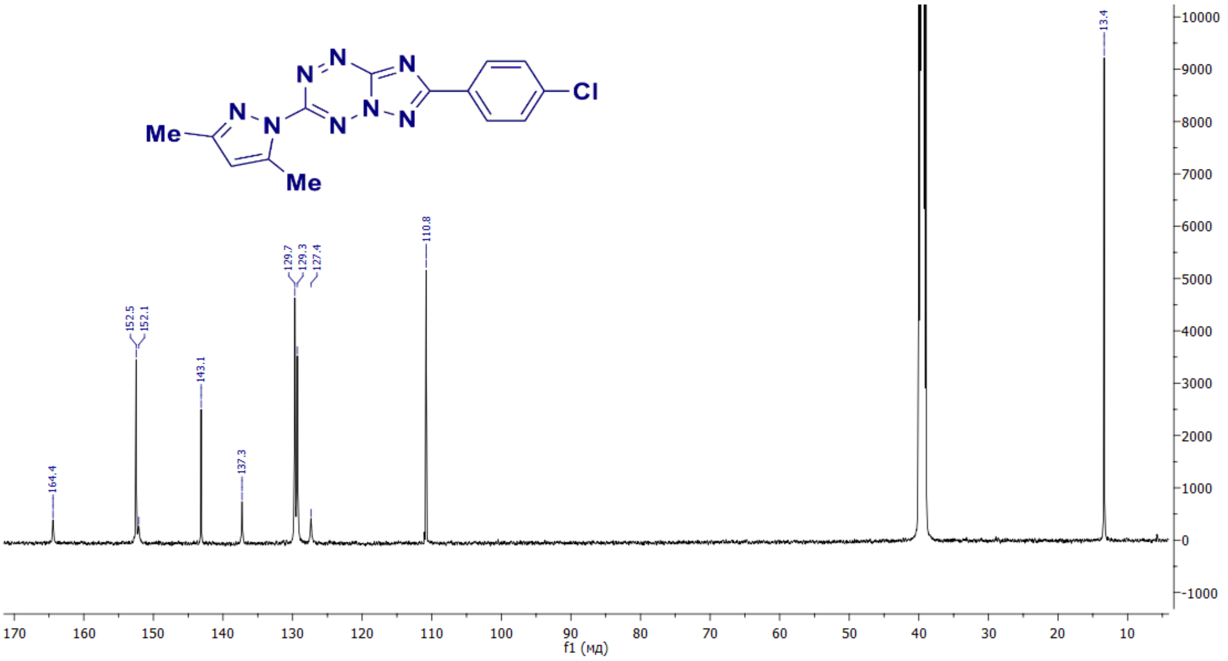

Figure S26. ^{13}C NMR spectrum of the 7-(4-chlorophenyl)-3-(3,5-dimethyl-1*H*-pyrazol-1-yl)-[1,2,4]triazolo[1,5-*b*][1,2,4,5]tetrazine (**3d**).

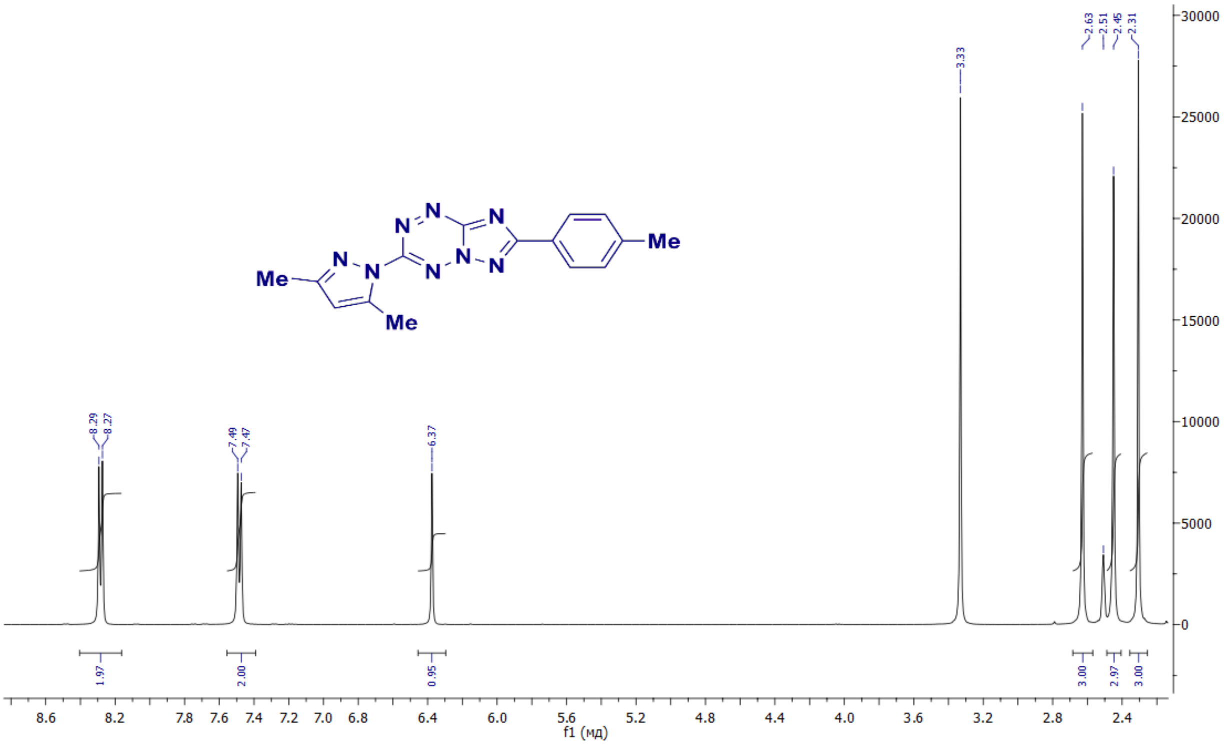

Figure S27. ^{1}H NMR spectrum of the 3-(3,5-dimethyl-1*H*-pyrazol-1-yl)-7-*p*-tolyl-[1,2,4]triazolo[1,5-*b*][1,2,4,5]tetrazine (**3e**).

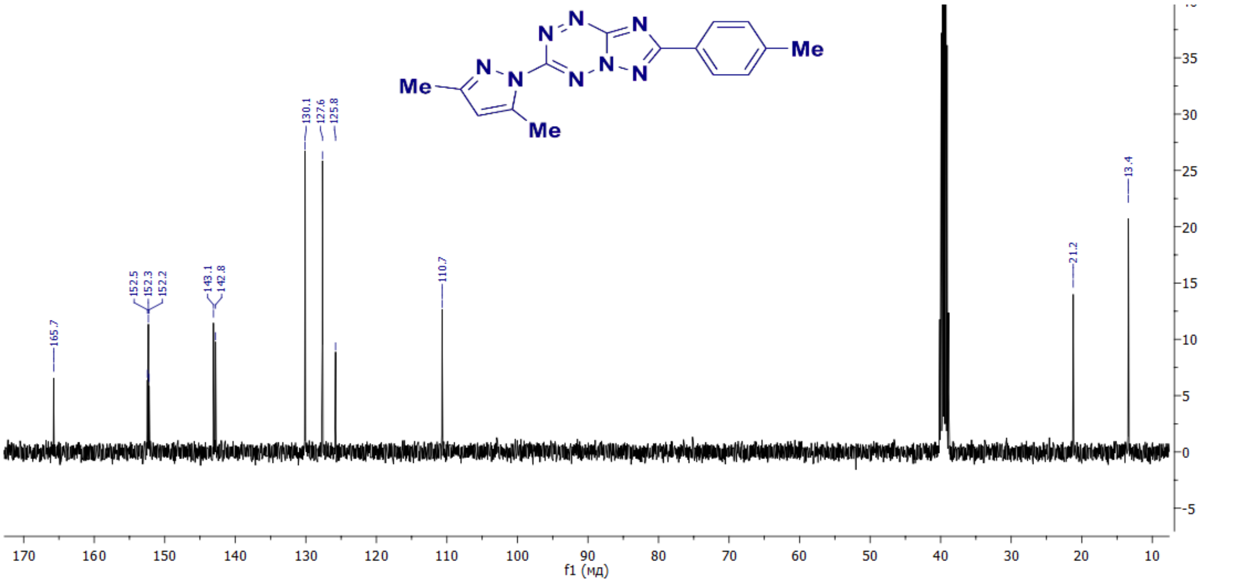

Figure S28. ^{13}C NMR spectrum of the 3-(3,5-dimethyl-1*H*-pyrazol-1-yl)-7-*p*-tolyl-[1,2,4]triazolo[1,5-*b*][1,2,4,5]tetrazine (**3e**).

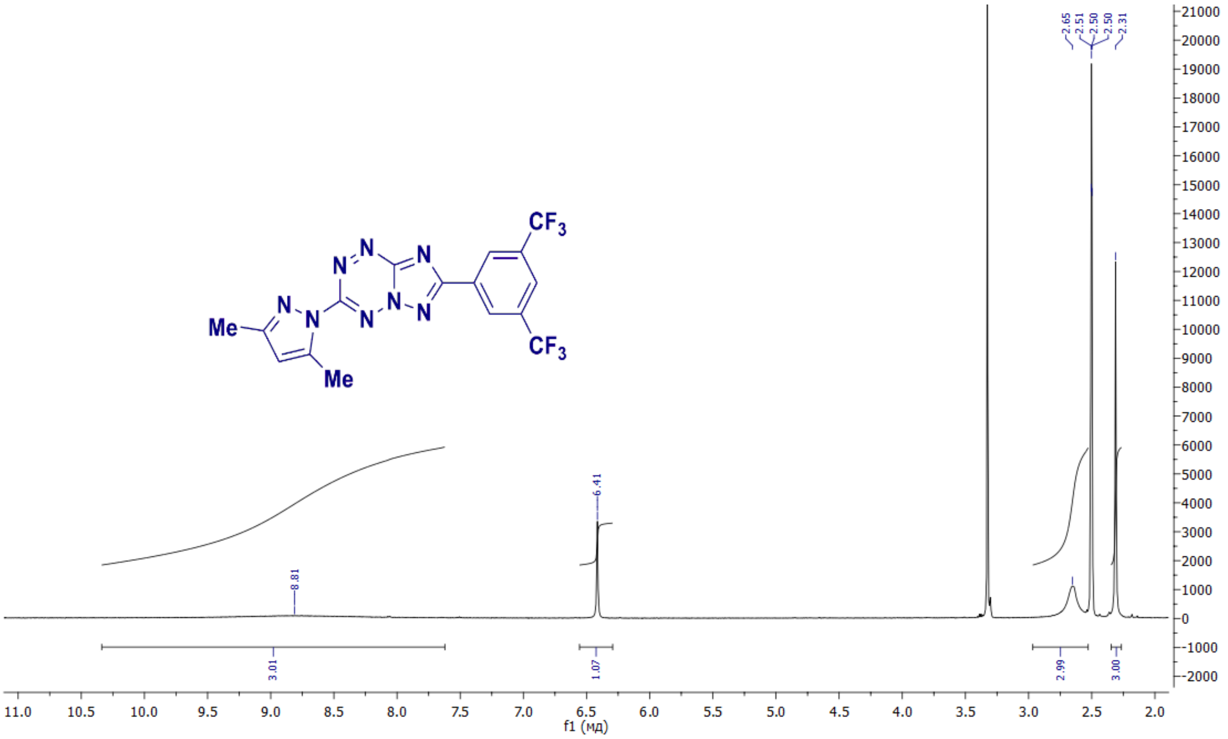

Figure S29. ^{1}H NMR spectrum of the 7-(3,5-bis(trifluoromethyl)phenyl)-3-(3,5-dimethyl-1*H*-pyrazol-1-yl)-[1,2,4]triazolo[1,5-*b*][1,2,4,5]tetrazine (**3f**).

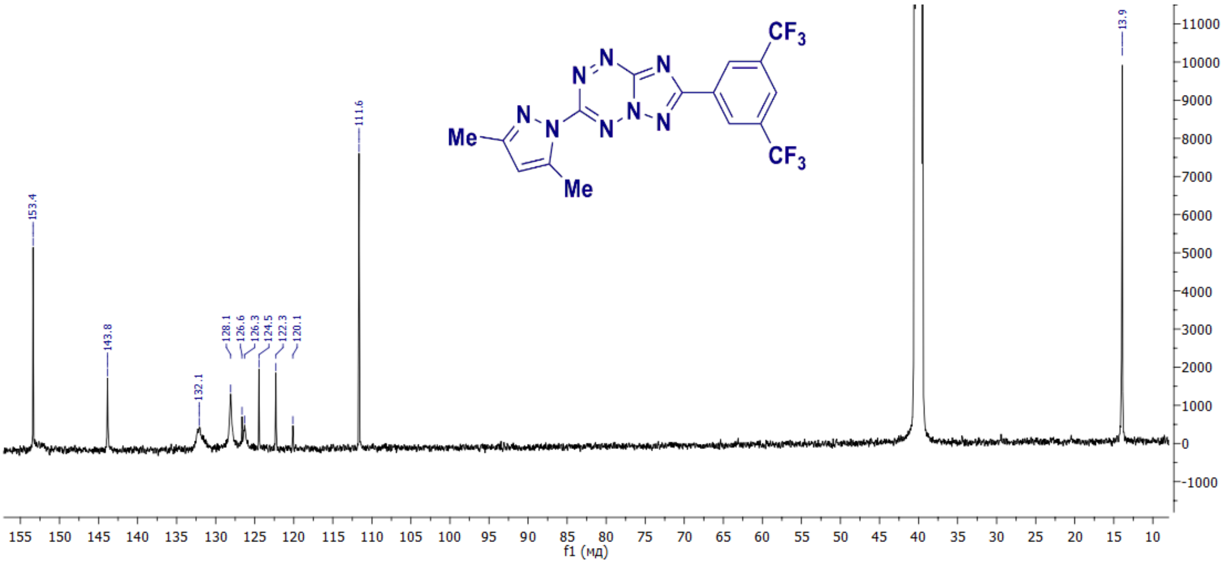

Figure S30. ^{13}C NMR spectrum of the 7-(3,5-bis(trifluoromethyl)phenyl)-3-(3,5-dimethyl-1*H*-pyrazol-1-yl)-[1,2,4]triazolo[1,5-*b*][1,2,4,5]tetrazine (**3f**).

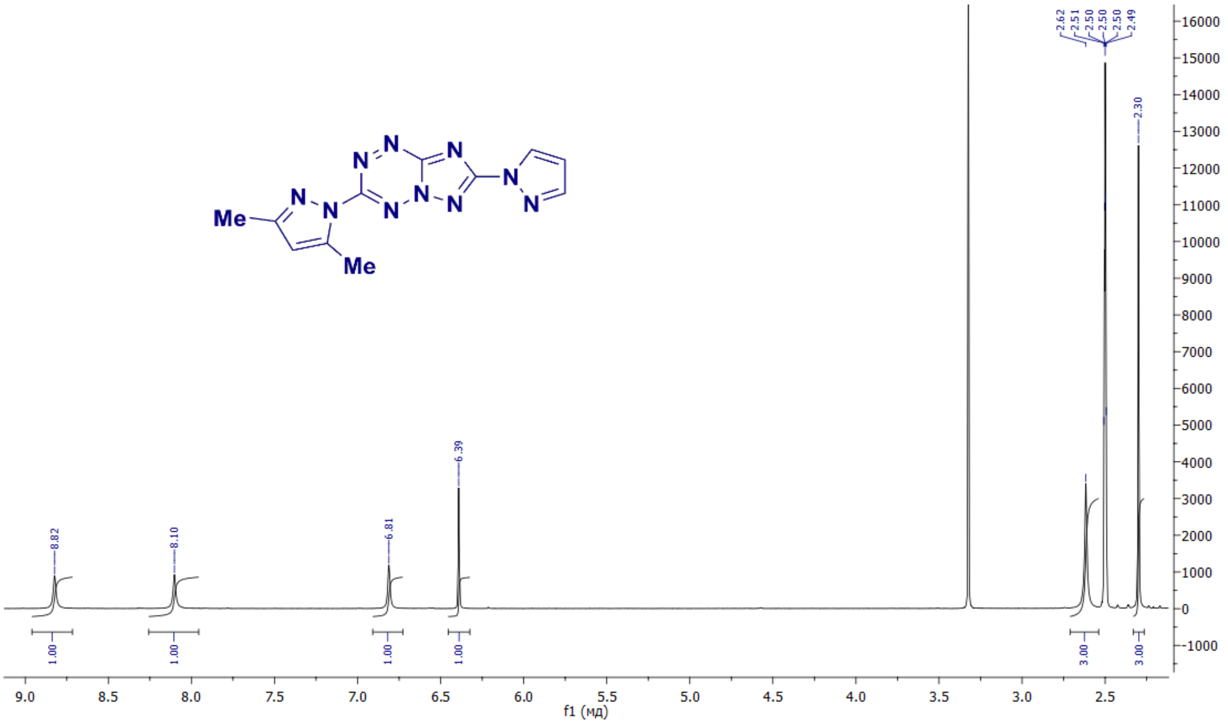

Figure S31. ^{1}H NMR spectrum of the 3-(3,5-dimethyl-1*H*-pyrazol-1-yl)-7-(1*H*-pyrazol-1-yl)-[1,2,4]triazolo[1,5-*b*][1,2,4,5]tetrazine (**3g**).

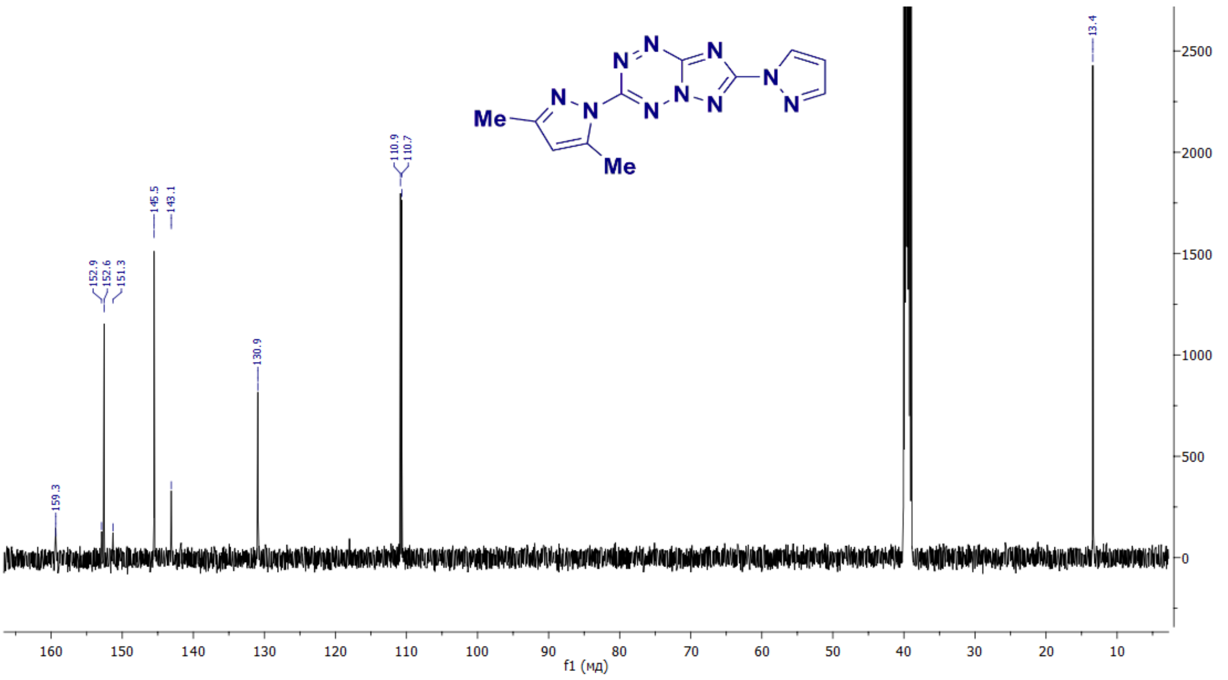

Figure S32. ^{13}C NMR spectrum of the 3-(3,5-dimethyl-1*H*-pyrazol-1-yl)-7-(1*H*-pyrazol-1-yl)-[1,2,4]triazolo[1,5-*b*][1,2,4,5]tetrazine (**3g**).

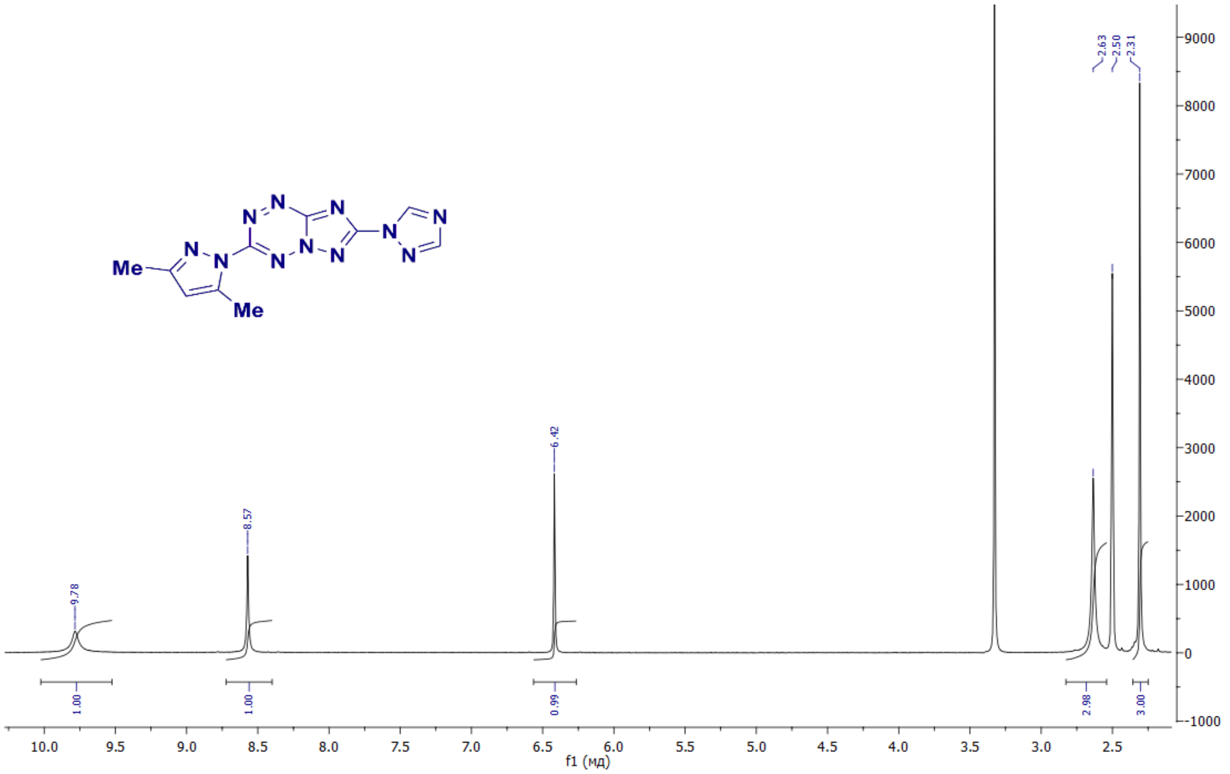

Figure S33. ^{1}H NMR spectrum of the 3-(3,5-dimethyl-1*H*-pyrazol-1-yl)-7-(1*H*-1,2,4-triazol-1-yl)-[1,2,4]triazolo[1,5-*b*][1,2,4,5]tetrazine (**3h**).

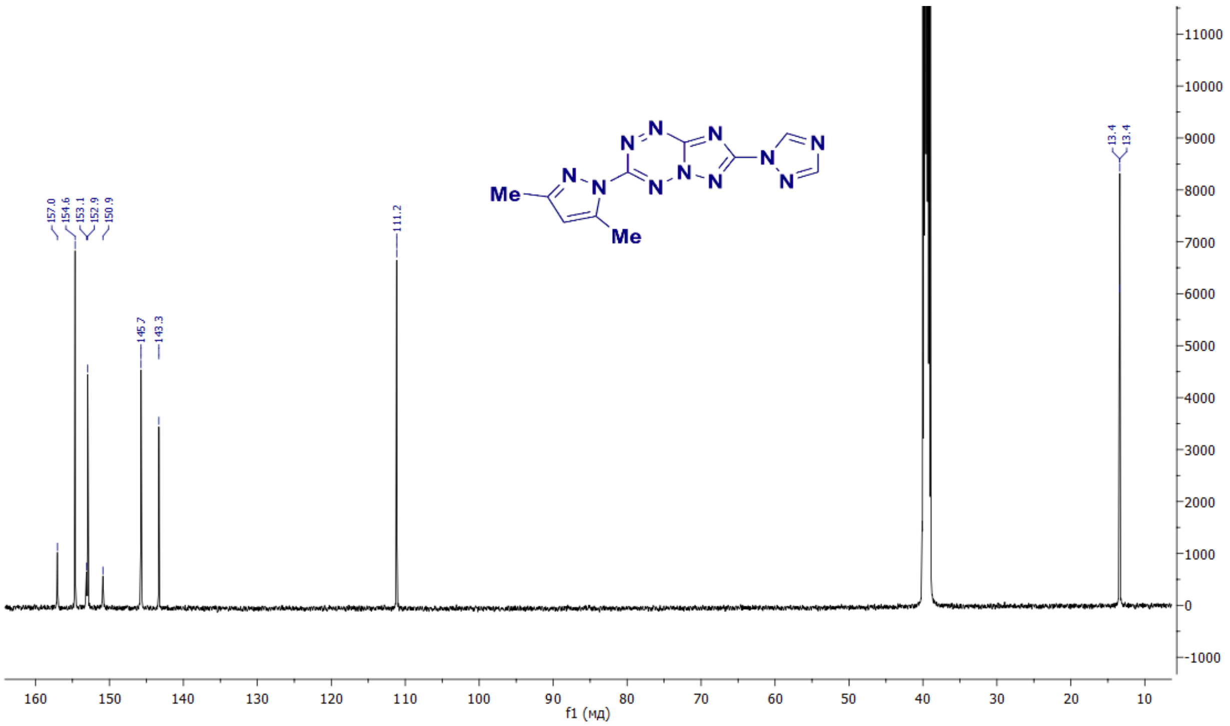

Figure S34. ^{13}C NMR spectrum of the 3-(3,5-dimethyl-1*H*-pyrazol-1-yl)-7-(1*H*-1,2,4-triazol-1-yl)-[1,2,4]triazolo[1,5-*b*][1,2,4,5]tetrazine (**3h**).

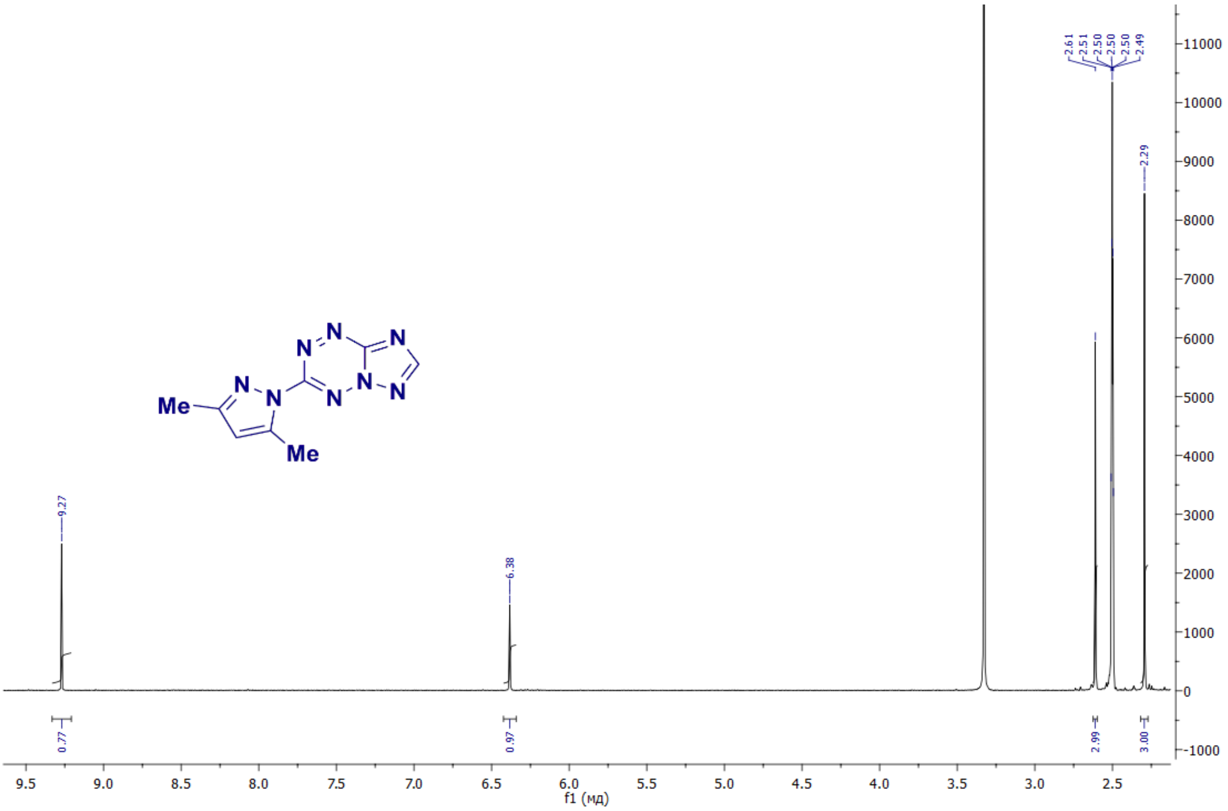

Figure S35. ^{1}H NMR spectrum of the 3-(3,5-dimethyl-1*H*-pyrazol-1-yl)-[1,2,4]triazolo[1,5-*b*][1,2,4,5]tetrazine (**3i**).

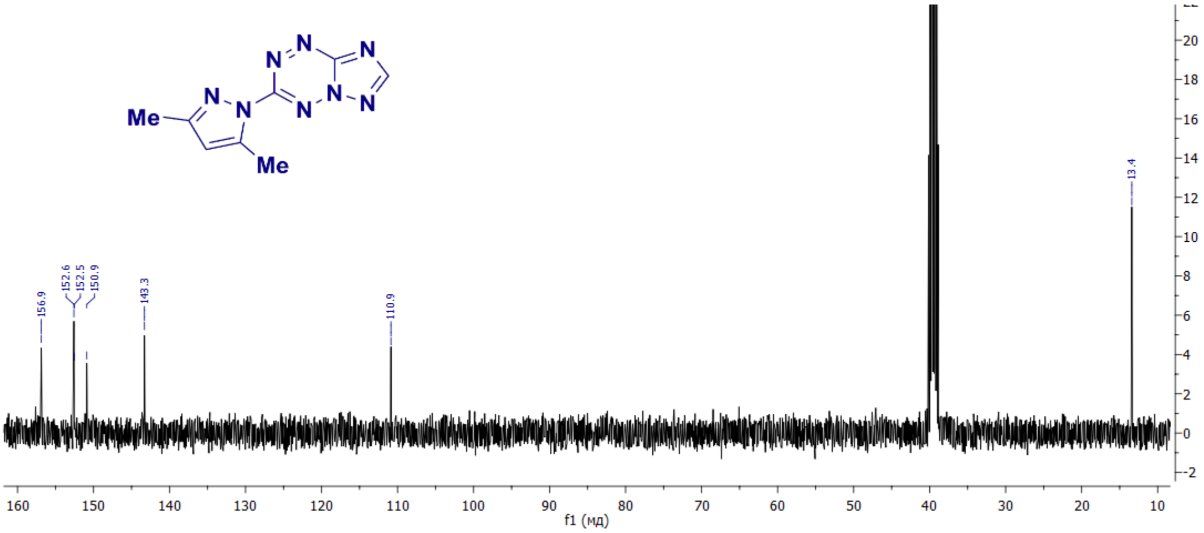

Figure S36. ^{13}C NMR spectrum of the 3-(3,5-dimethyl-1*H*-pyrazol-1-yl)-[1,2,4]triazolo[1,5-*b*][1,2,4,5]tetrazine (**3i**).

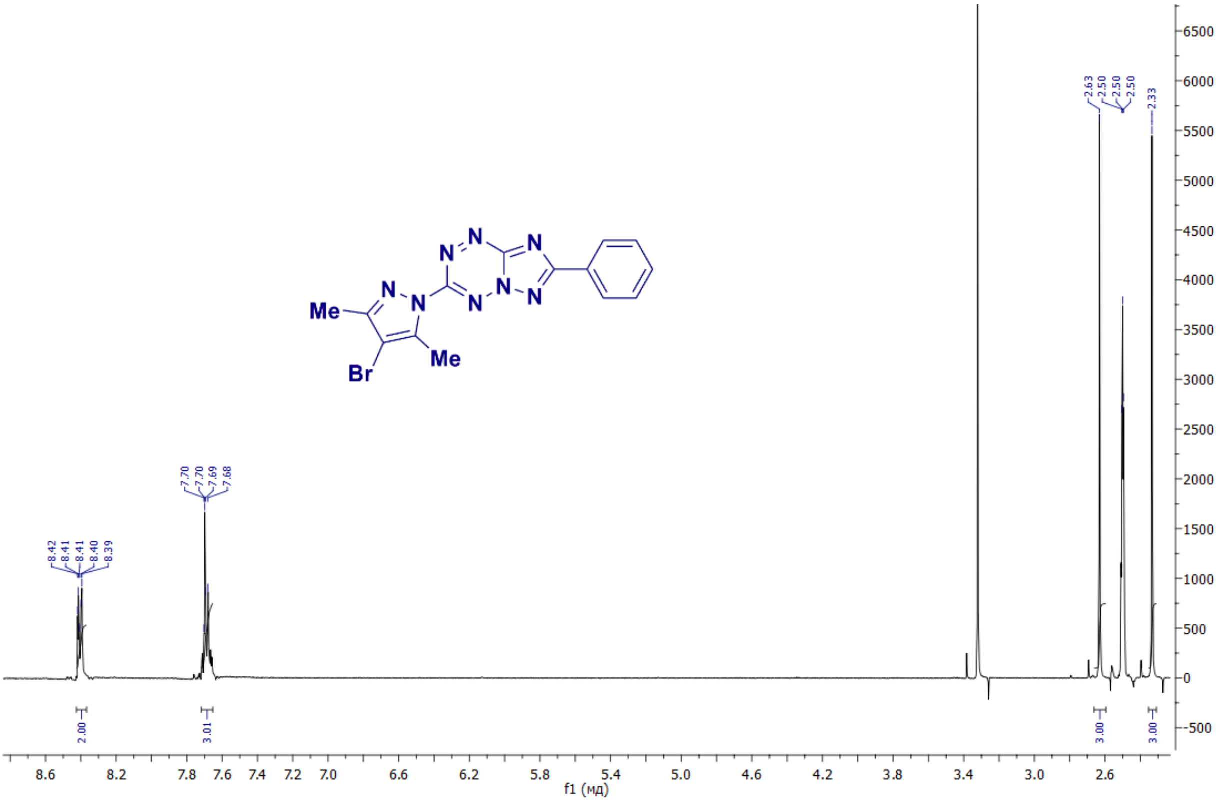

Figure S37. ^{1}H NMR spectrum of the 3-(4-bromo-3,5-dimethyl-1*H*-pyrazol-1-yl)-7-phenyl-[1,2,4]triazolo[1,5-*b*][1,2,4,5]tetrazine (**3j**).

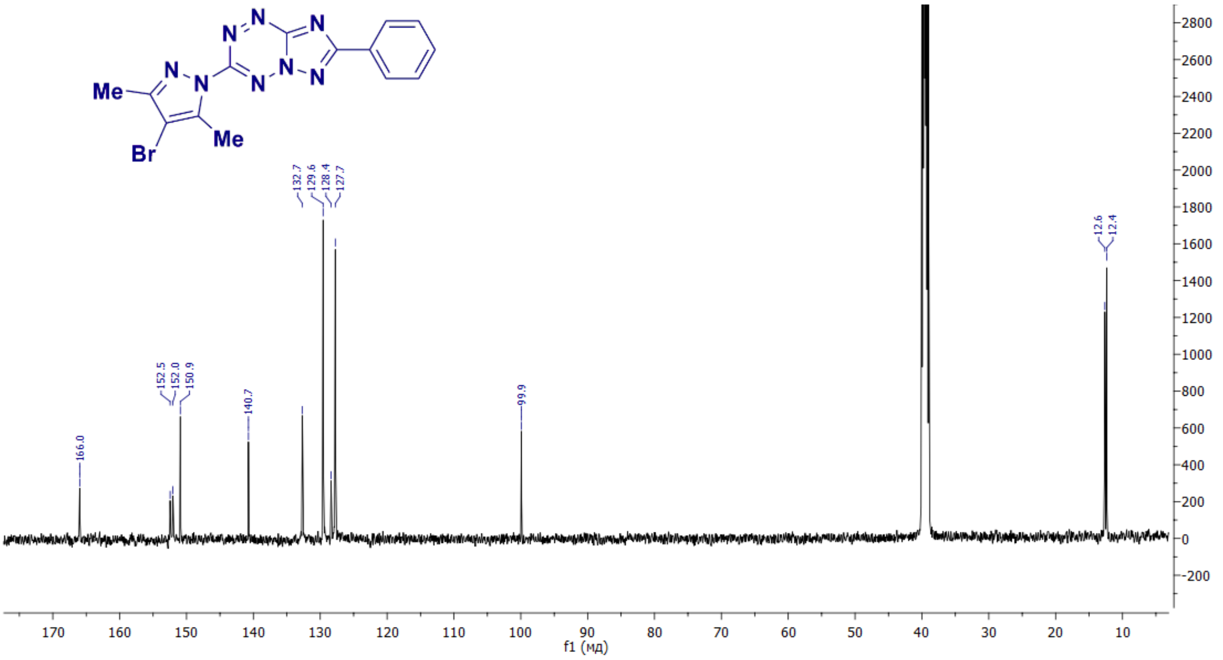

Figure S38. ^{13}C NMR spectrum of the 3-(4-bromo-3,5-dimethyl-1*H*-pyrazol-1-yl)-7-phenyl-[1,2,4]triazolo[1,5-*b*][1,2,4,5]tetrazine (**3j**).

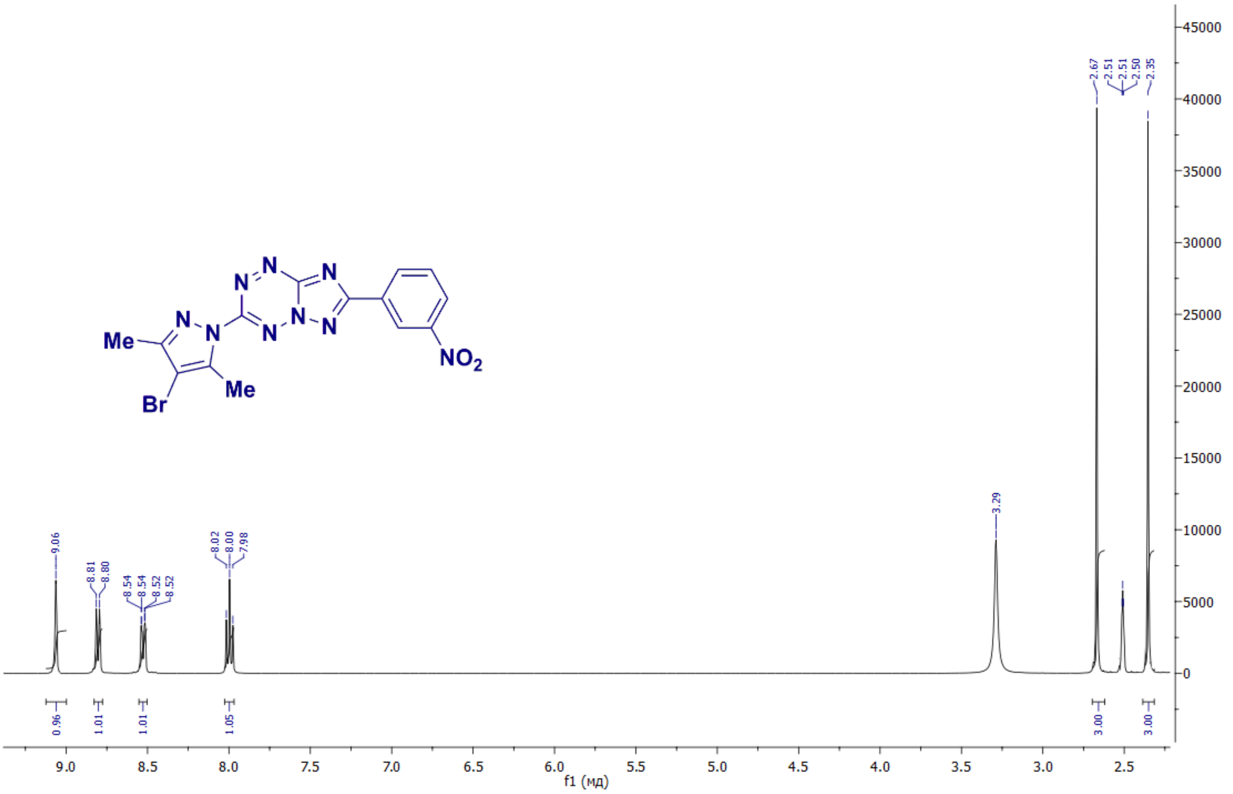

Figure S39. ^{1}H NMR spectrum of the 3-(4-bromo-3,5-dimethyl-1*H*-pyrazol-1-yl)-7-(3-nitrophenyl)-[1,2,4]triazolo[1,5-*b*][1,2,4,5]tetrazine (**3k**).

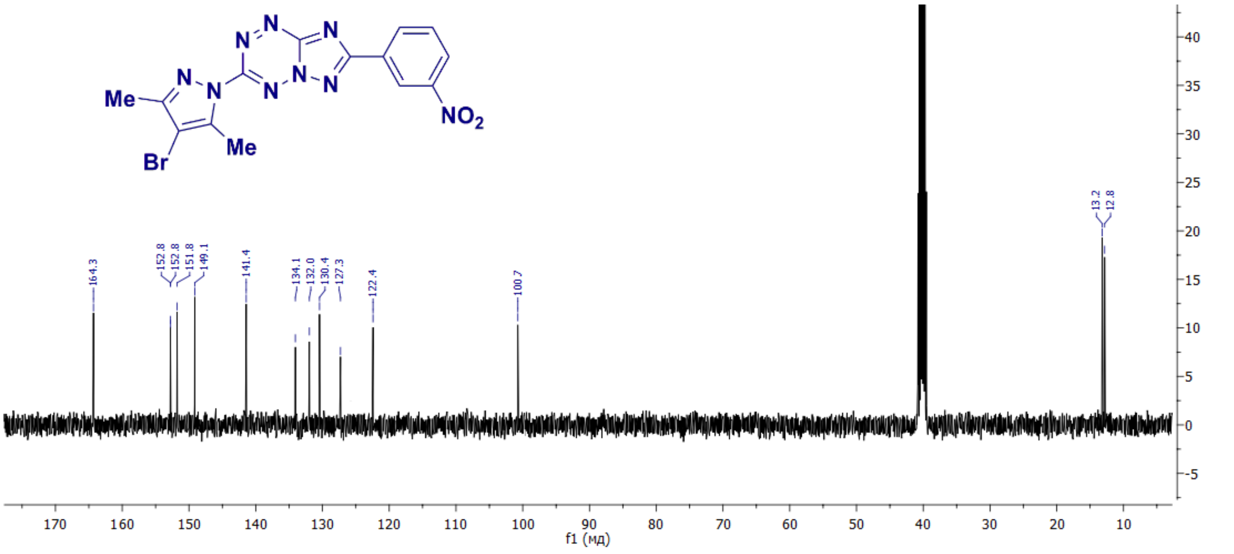

Figure S40. ^{13}C NMR spectrum of the 3-(4-bromo-3,5-dimethyl-1*H*-pyrazol-1-yl)-7-(3-nitrophenyl)-[1,2,4]triazolo[1,5-*b*][1,2,4,5]tetrazine (**3k**).

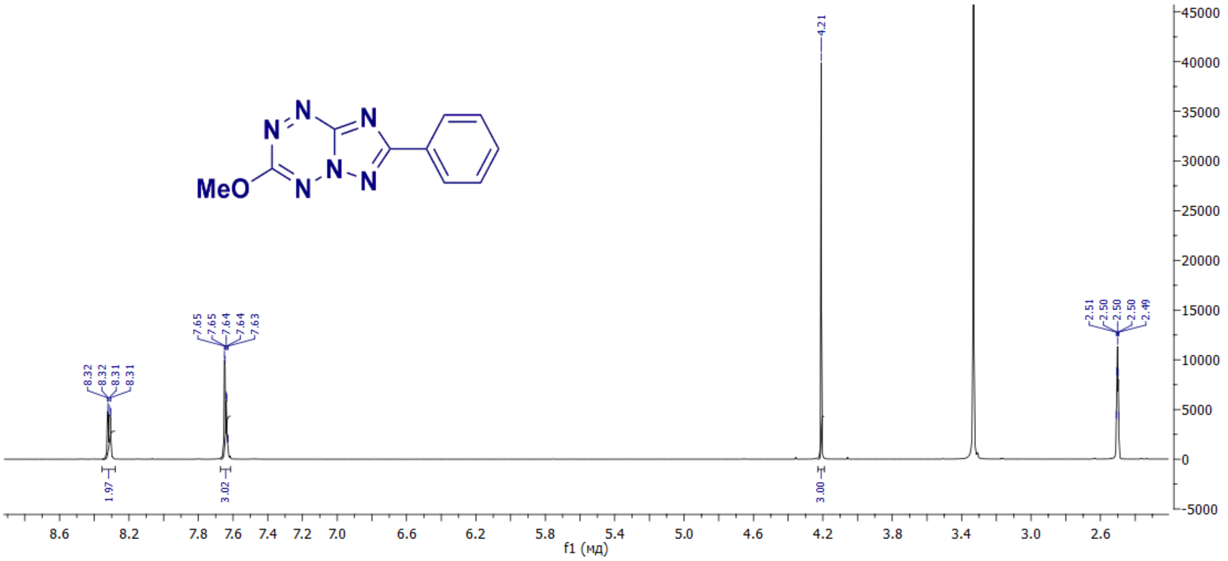

Figure S41. ^{1}H NMR spectrum of the 3-methoxy-7-phenyl-[1,2,4]triazolo[1,5-*b*][1,2,4,5]tetrazine (**4a**).

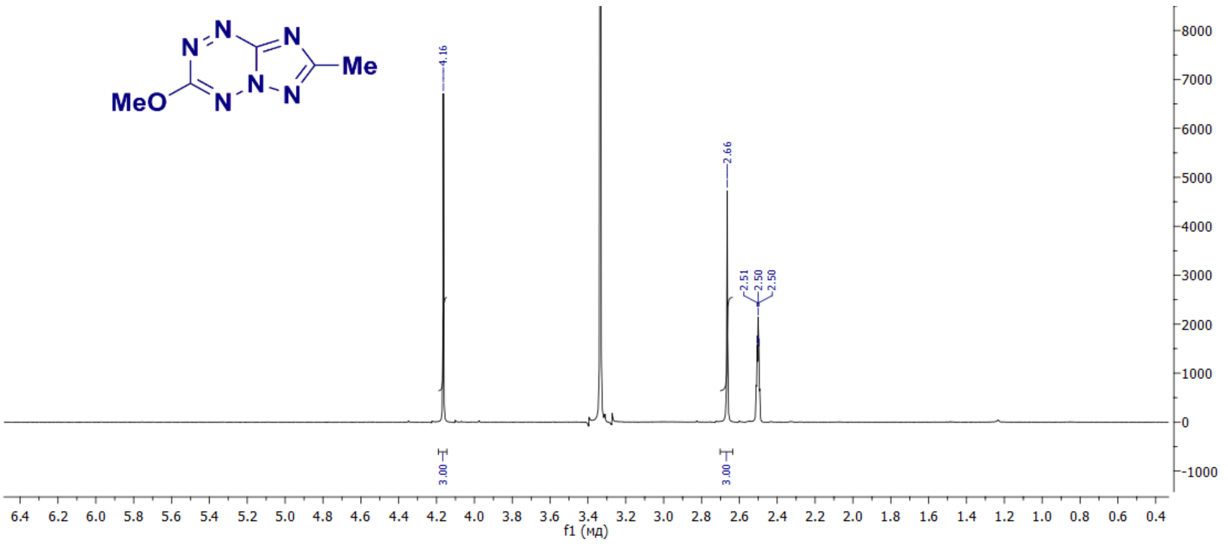

Figure S42. ^{1}H NMR spectrum of the 3-methoxy-7-methyl-[1,2,4]triazolo[1,5-*b*][1,2,4,5]tetrazine (**5a**).

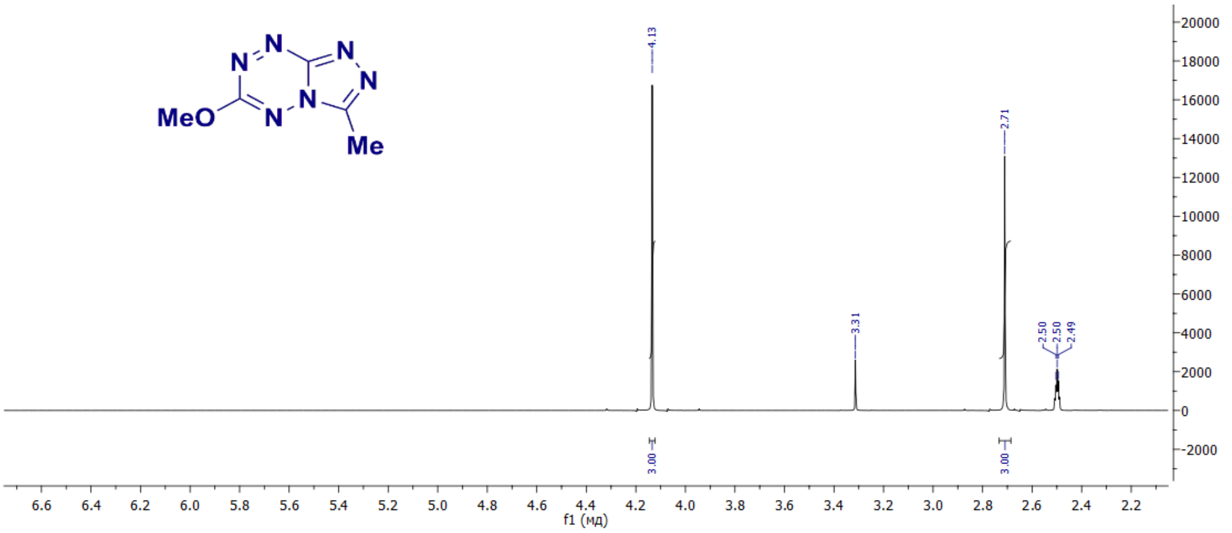

Figure S43. ^{1}H NMR spectrum of the 3-methoxy-6-methyl-[1,2,4]triazolo[4,3-*b*][1,2,4,5]tetrazine (**11a**).

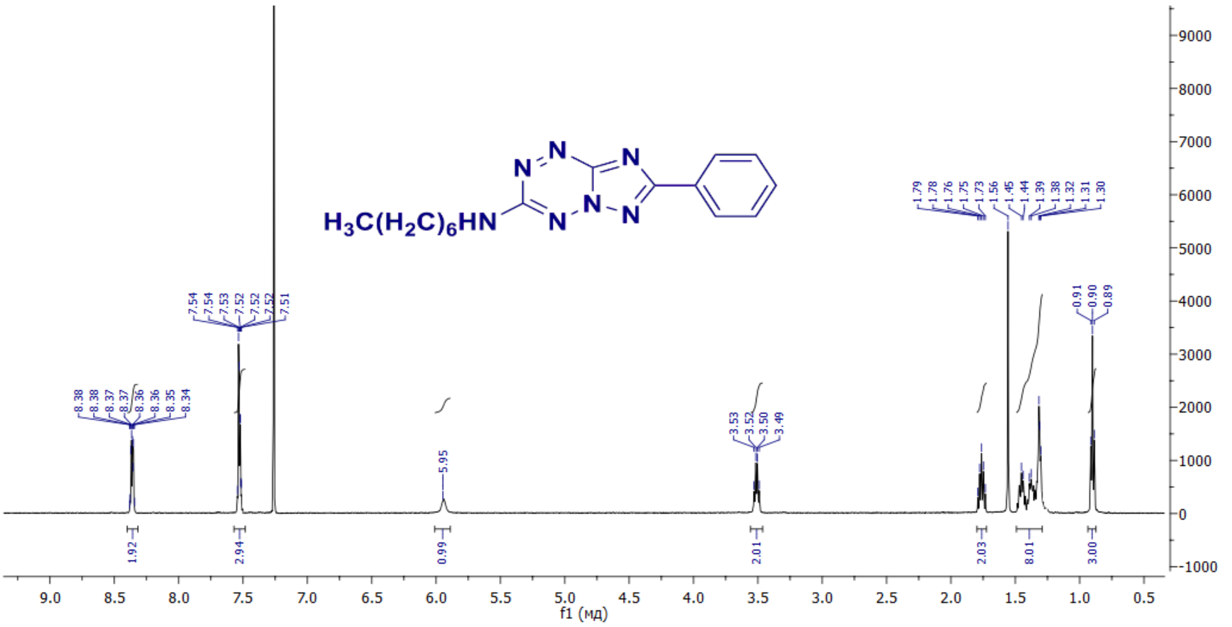

Figure S44. ^{1}H NMR spectrum of the 3-heptylamino-7-phenyl-[1,2,4]triazolo[1,5-*b*][1,2,4,5]tetrazine (**4b**).

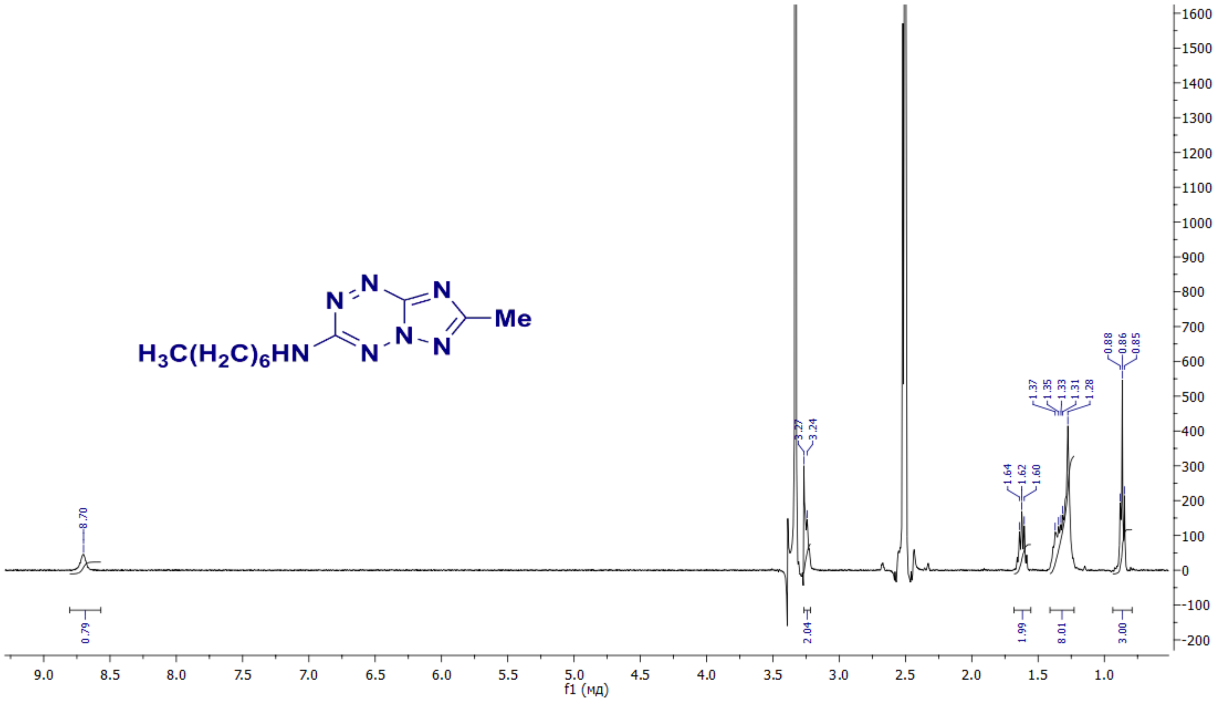

Figure S45. ^{1}H NMR spectrum of the 3-heptylamino-7-methyl-[1,2,4]triazolo[1,5-*b*][1,2,4,5]tetrazine (**5b**).

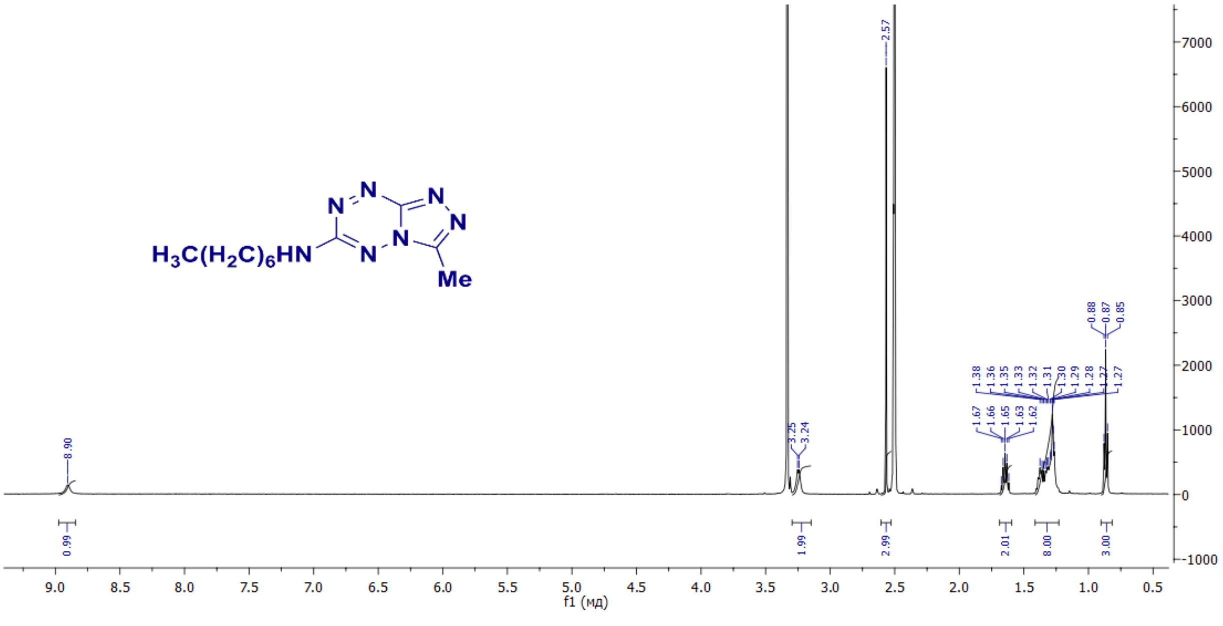

Figure S46. ^{1}H NMR spectrum of the 3-heptylamino-6-methyl-[1,2,4]triazolo[4,3-*b*][1,2,4,5]tetrazine (**11b**).

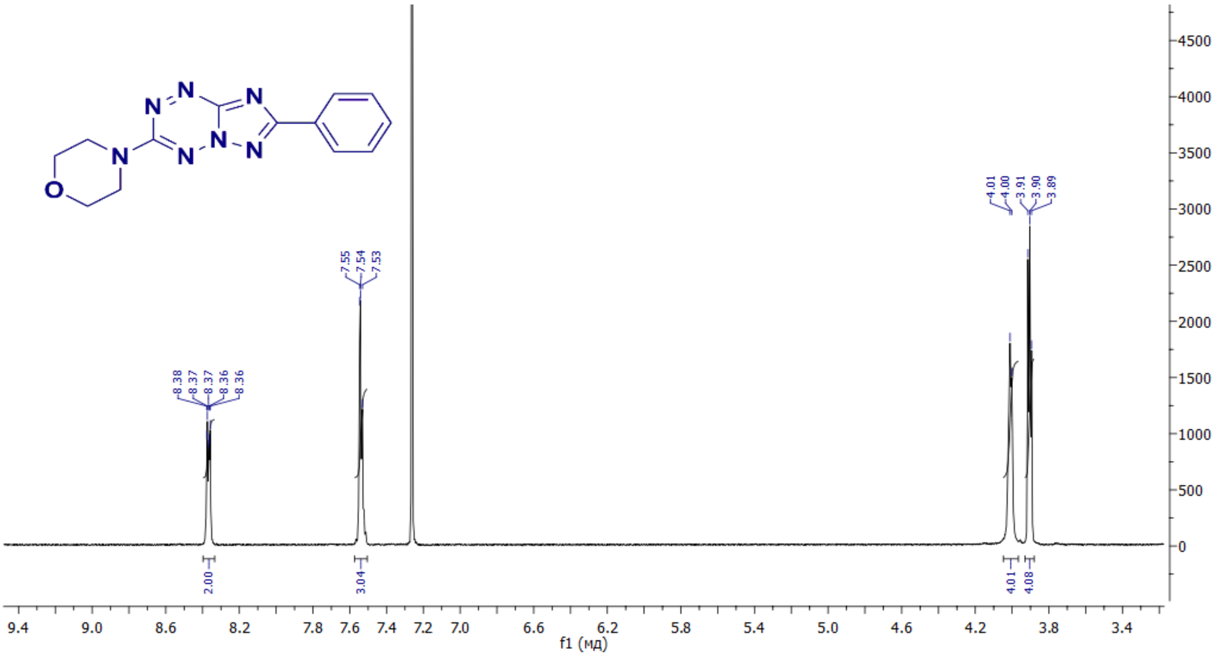

Figure S47. ^{1}H NMR spectrum of the 3-morpholino-7-phenyl-[1,2,4]triazolo[1,5-*b*][1,2,4,5]tetrazine (**4c**).

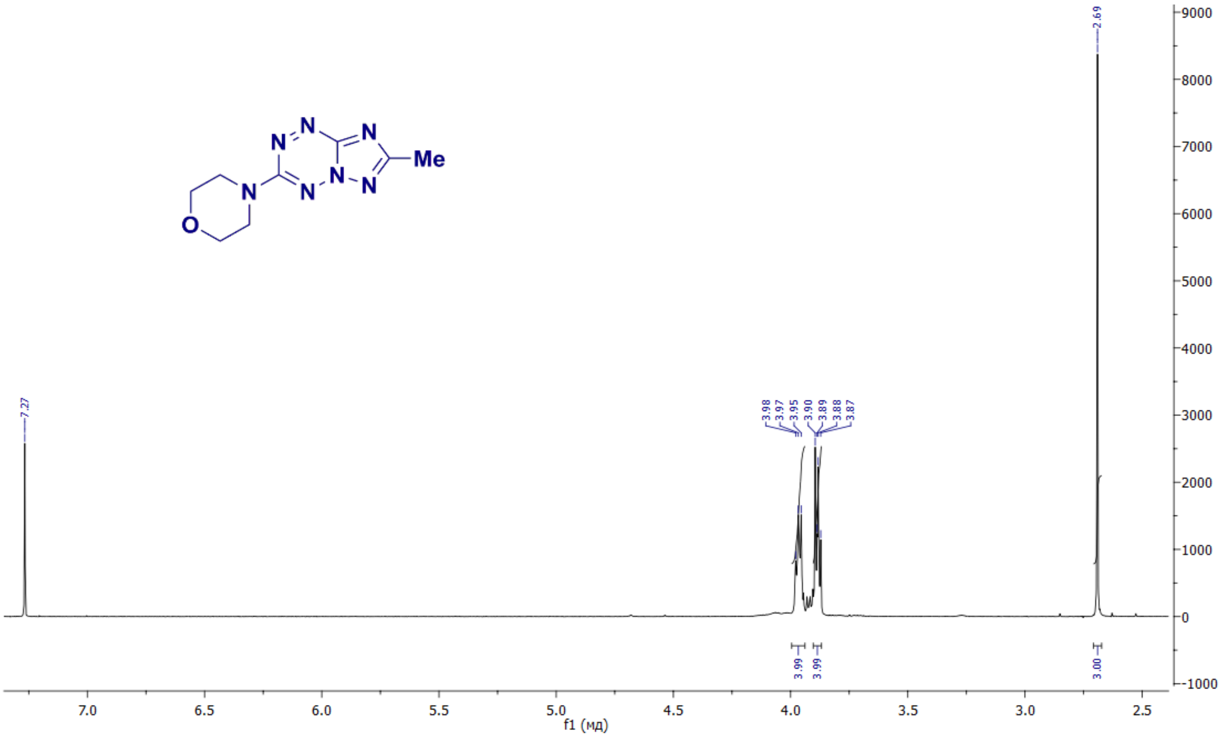

Figure S48. ^{1}H NMR spectrum of the 7-methyl-3-morpholino-[1,2,4]triazolo[1,5-*b*][1,2,4,5]tetrazine (**5c**).

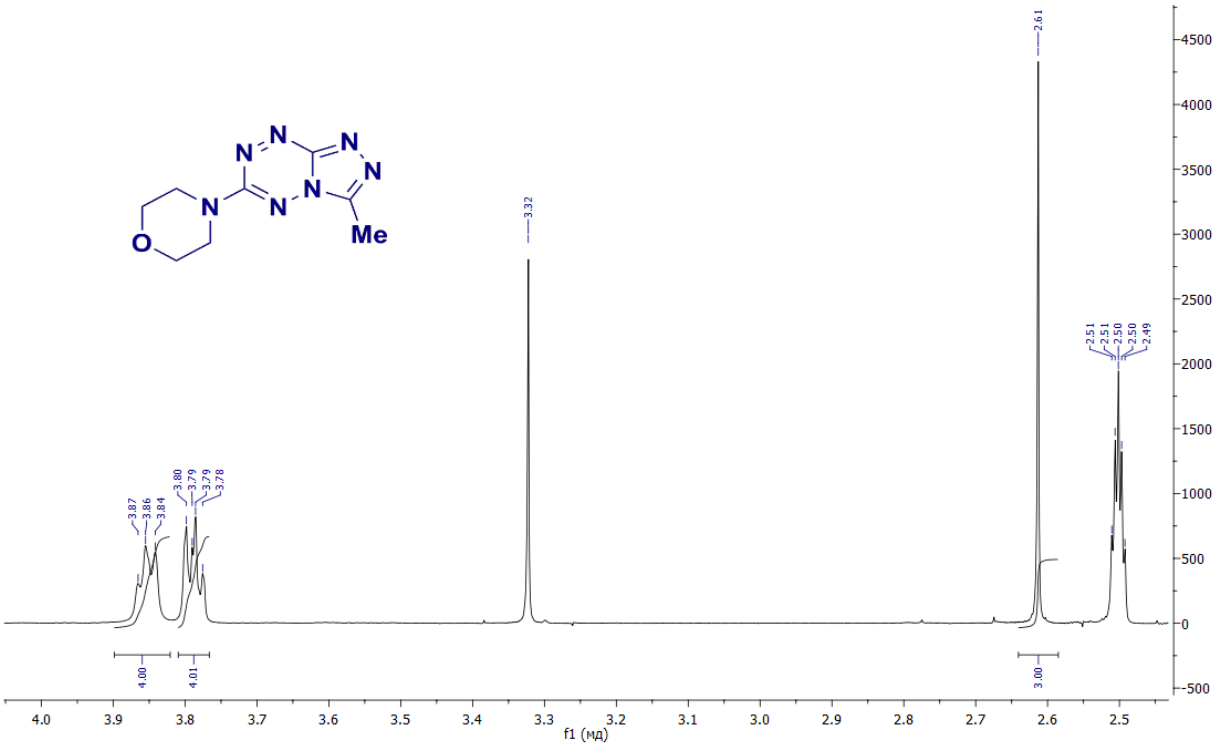

Figure S49. ^{1}H NMR spectrum of the 6-methyl-3-morpholino-[1,2,4]triazolo[4,3-*b*][1,2,4,5]tetrazine (**11c**).

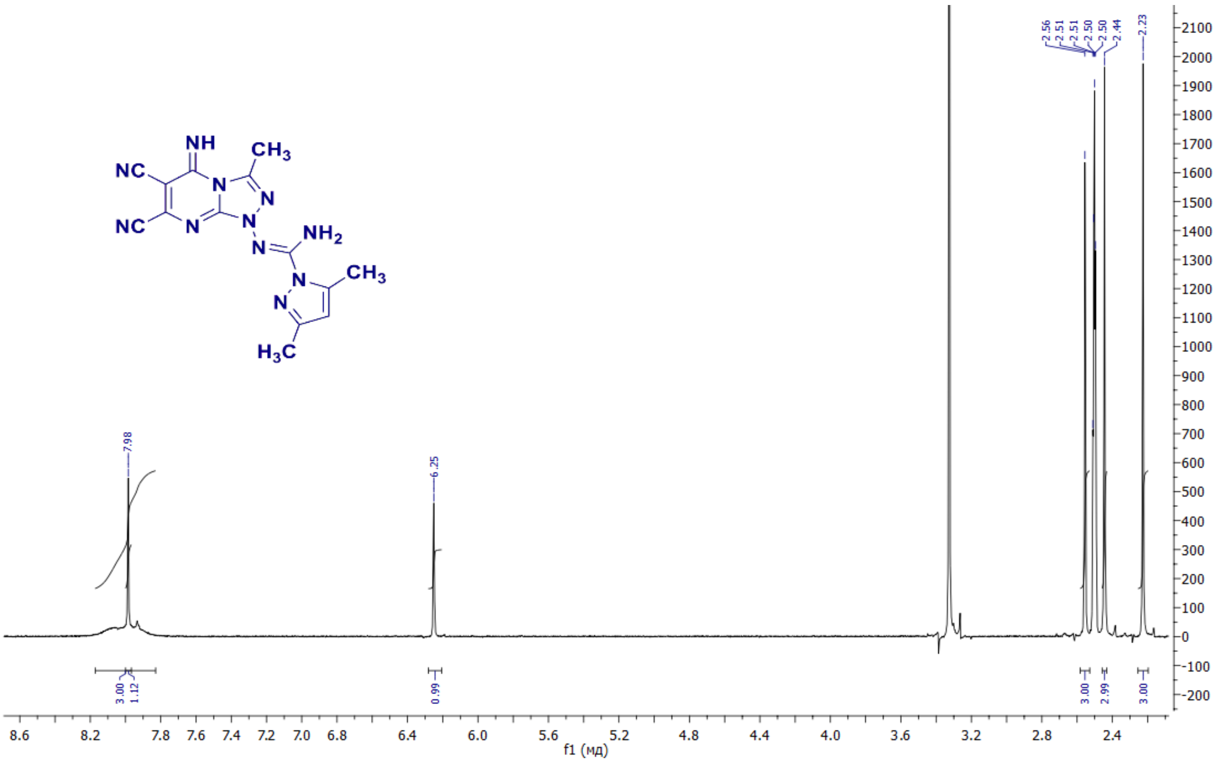

Figure S50. ^{1}H NMR spectrum of the *N'*-(6,7-dicyano-5-imino-3-methyl-[1,2,4]triazolo[4,3-*a*]pyrimidin-1(5*H*)-yl)-3,5-dimethyl-1*H*-pyrazole-1-carboximidamide (**6a**).

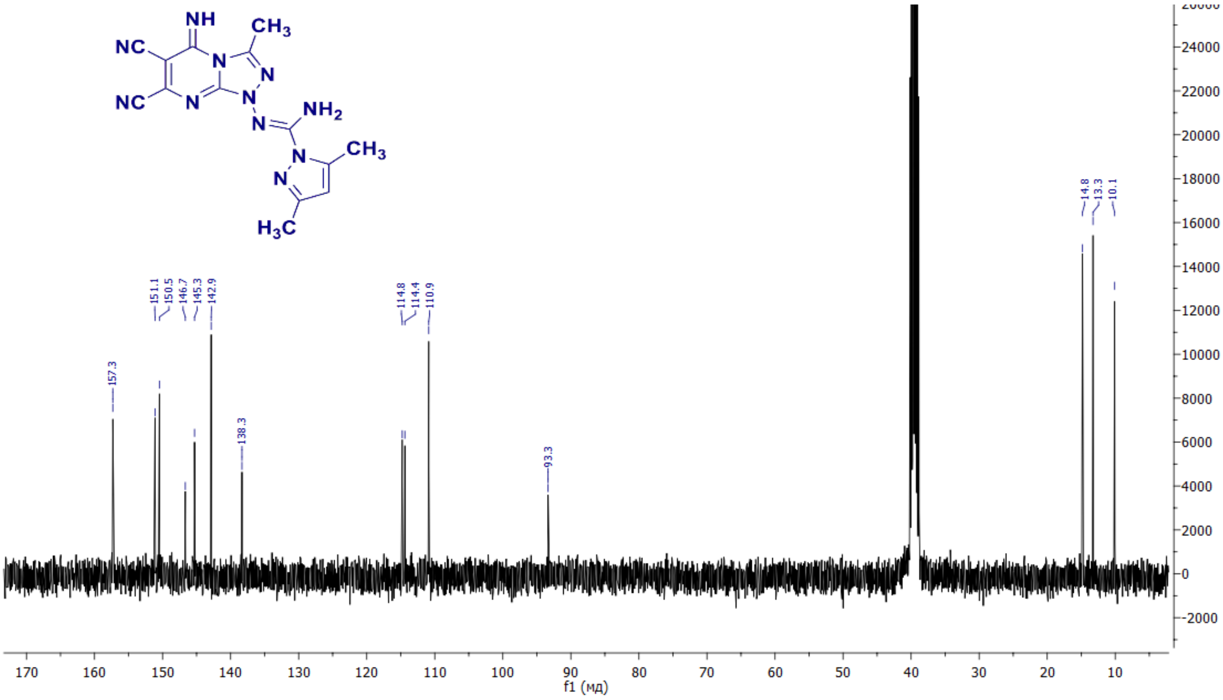

Figure S51. ^{13}C NMR spectrum of the *N'*-(6,7-dicyano-5-imino-3-methyl-[1,2,4]triazolo[4,3-*a*]pyrimidin-1(5*H*)-yl)-3,5-dimethyl-1*H*-pyrazole-1-carboximidamide (**6a**).

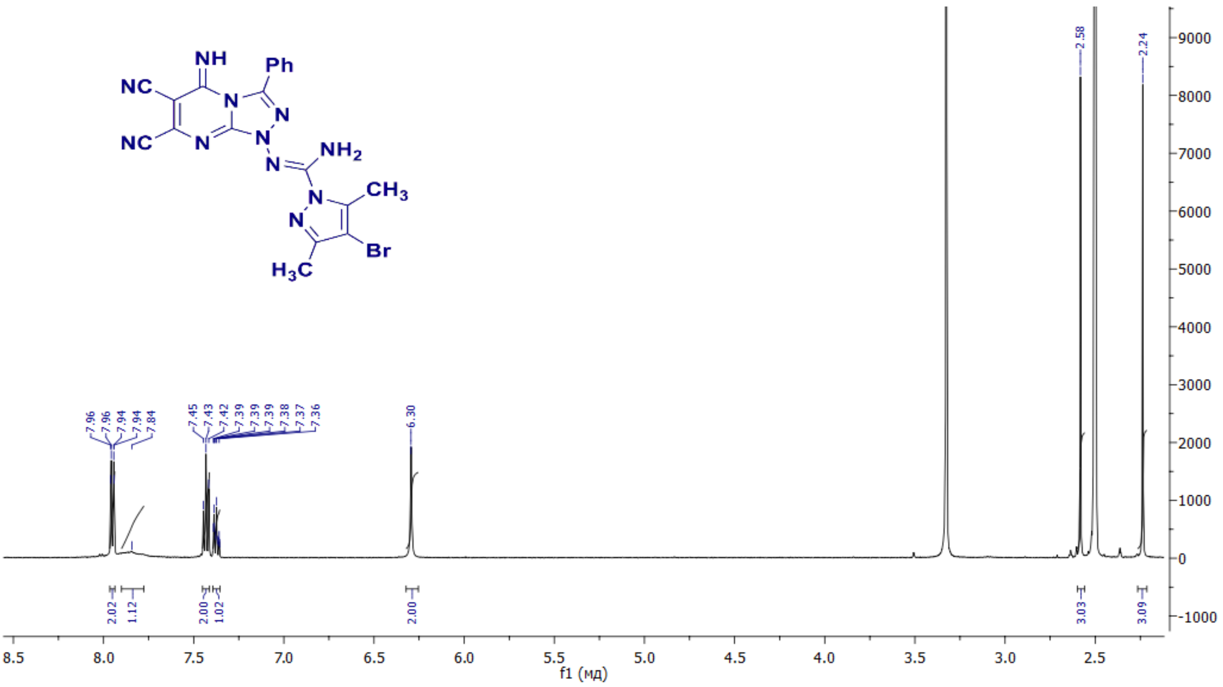

Figure S52. ^{1}H NMR spectrum of the 4-bromo-*N'*-(6,7-dicyano-5-imino-3-phenyl-[1,2,4]triazolo[4,3-*a*]pyrimidin-1(5*H*)-yl)-3,5-dimethyl-1*H*-pyrazole-1-carboximidamide (**6b**).

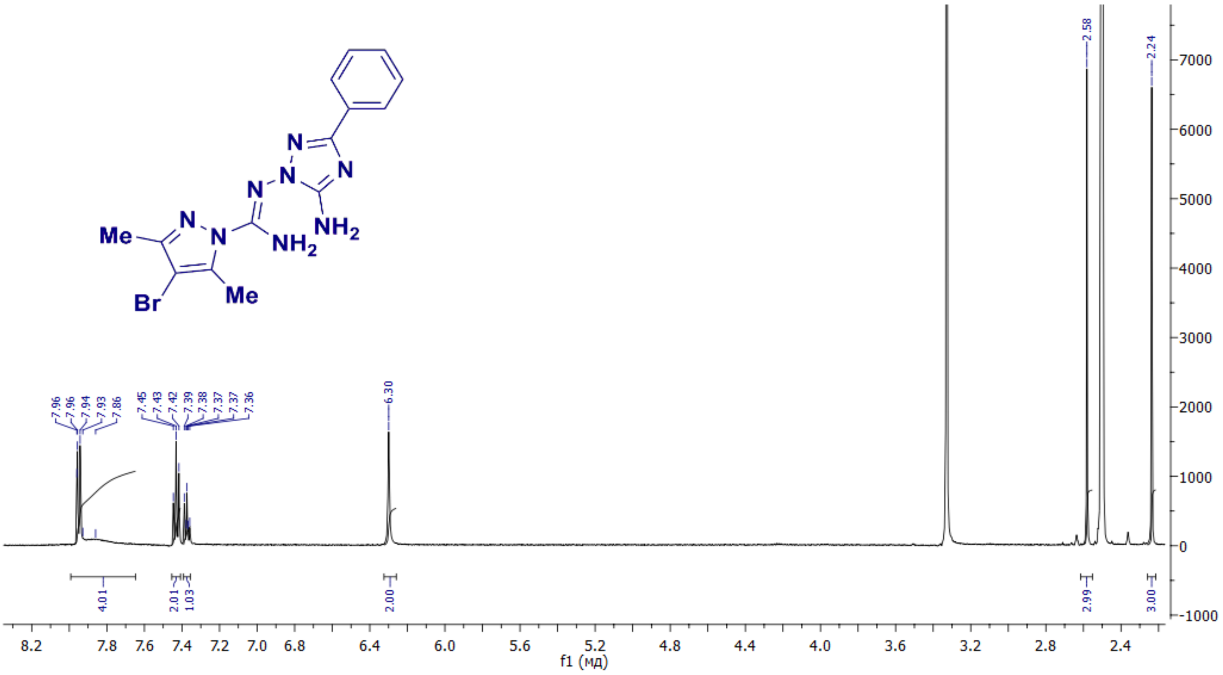

Figure S53. ^{1}H NMR spectrum of the *N'*-(5-amino-3-phenyl-1*H*-1,2,4-triazol-1-yl)-4-bromo-3,5-dimethyl-1*H*-pyrazole-1-carboximidamide (**7**).

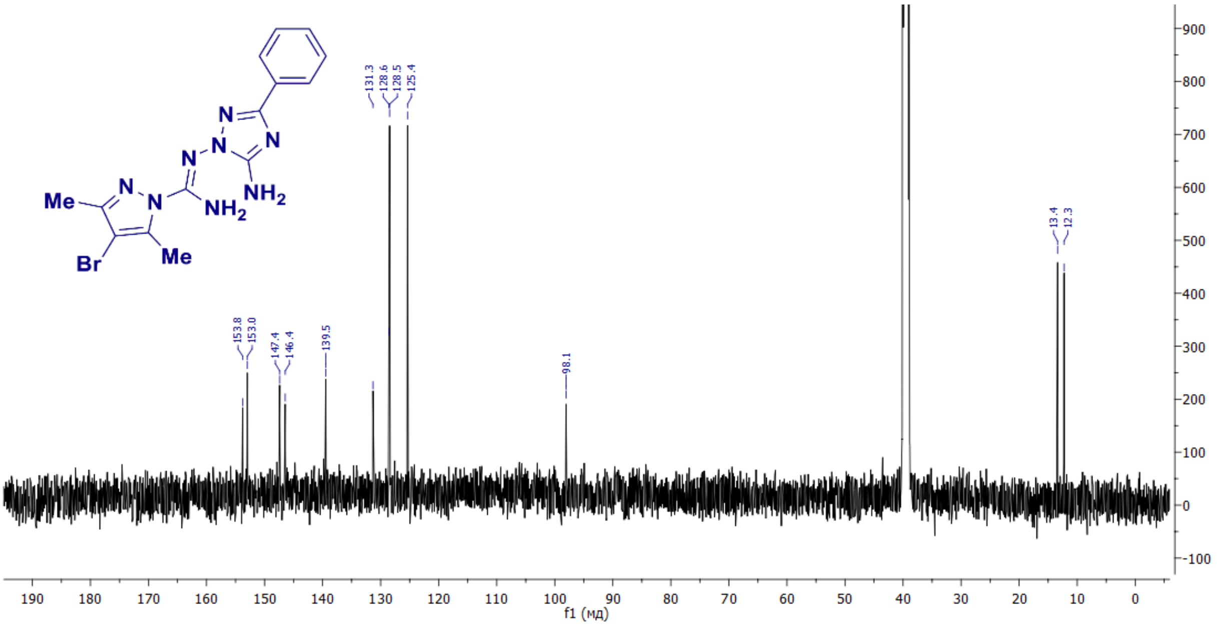

Figure S54. ^{13}C NMR spectrum of the *N'*-(5-amino-3-phenyl-1*H*-1,2,4-triazol-1-yl)-4-bromo-3,5-dimethyl-1*H*-pyrazole-1-carboximidamide (**7**).

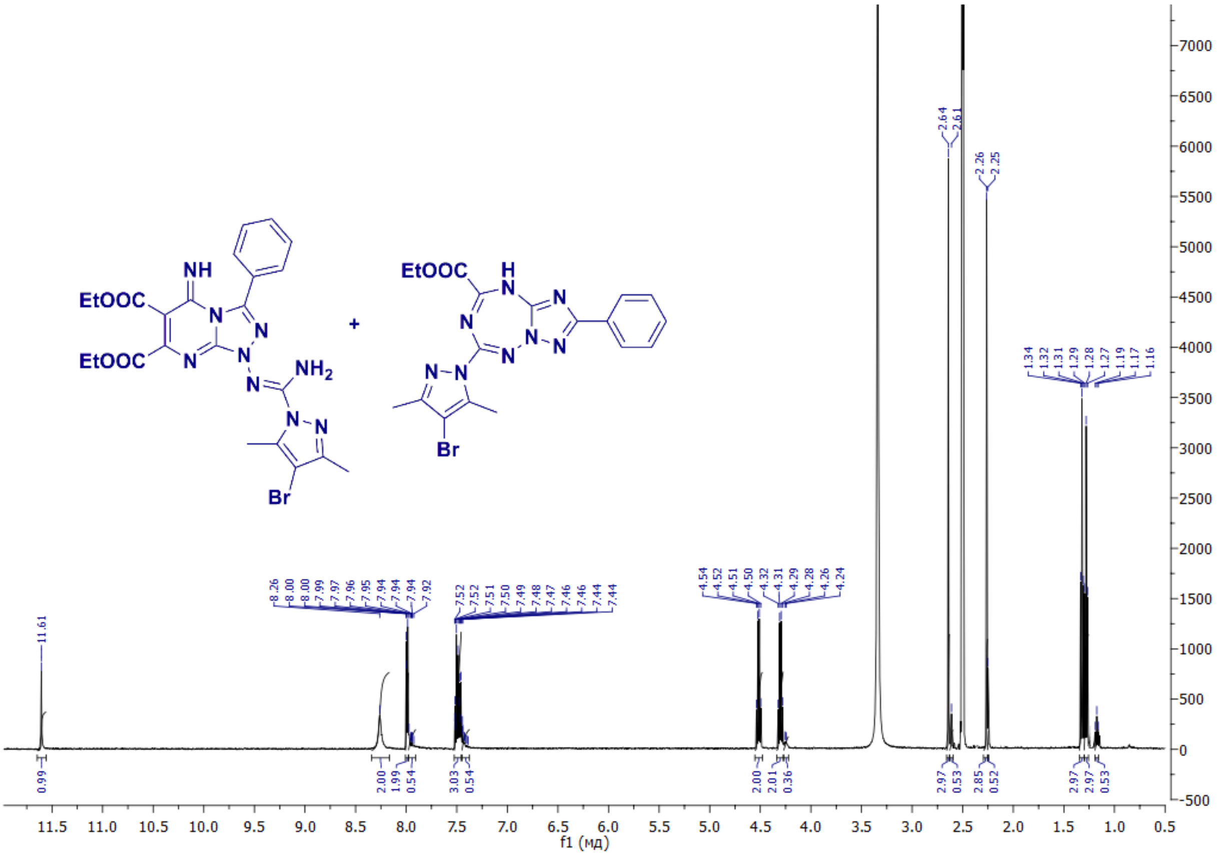

Figure S55. ^{1}H NMR spectrum of the 6-(4-bromo-3,5-dimethyl-1*H*-pyrazol-1-yl)-8-ethoxycarbonyl-2-phenyl-7*H*-[1,2,4]triazolo[1,5-*b*][1,2,4,6]tetrazepine (**8**) and of the 1-(amino(4-bromo-3,5-dimethyl-1*H*-pyrazol-1-yl)methyleneamino)-6,7-diethoxycarbonyl-5-imino-3-phenyl-1,5-dihydro-[1,2,4]triazolo[4,3-*a*]pyrimidine (**9**).
